# GBA3: a polymorphic pseudogene in humans that experienced repeated gene loss during mammalian evolution

**DOI:** 10.1038/s41598-020-68106-y

**Published:** 2020-07-14

**Authors:** Monica Lopes-Marques, Catarina Serrano, Ana R. Cardoso, Renato Salazar, Susana Seixas, António Amorim, Luisa Azevedo, Maria J. Prata

**Affiliations:** 1 i3S- Instituto de Investigação e Inovação em Saúde, Population Genetics and Evolution Group, Universidade do Porto, Rua Alfredo Allen 208, 4200-135 Porto, Portugal; 2 IPATIMUP-Institute of Molecular Pathology and Immunology, University of Porto, Rua Júlio Amaral de Carvalho 45, 4200-135 Porto, Portugal; 3 Department of Biology, Faculty of Sciences, University of Porto, Rua do Campo Alegre, s/n, 4169-007 Porto, Portugal

**Keywords:** Evolutionary genetics, Molecular evolution, Population genetics, Evolutionary biology, Population genetics, Evolution, Genetics

## Abstract

The gene encoding the cytosolic β-glucosidase GBA3 shows pseudogenization due to a truncated allele (rs358231) that is polymorphic in humans. Since this enzyme is involved in the transformation of many plant β-glycosides, this particular case of gene loss may have been influenced by dietary adaptations during evolution. In humans, apart from the inactivating allele, we found that *GBA3* accumulated additional damaging mutations, implying an extensive *GBA3* loss. The allelic distribution of loss-of-function alleles revealed significant differences between human populations which can be partially related with their staple diet. The analysis of mammalian orthologs disclosed that *GBA3* underwent at least nine pseudogenization events. Most events of pseudogenization occurred in carnivorous lineages, suggesting a possible link to a β-glycoside poor diet. However, *GBA3* was also lost in omnivorous and herbivorous species, hinting that the physiological role of GBA3 is not fully understood and other unknown causes may underlie *GBA3* pseudogenization. Such possibility relies upon a putative role in sialic acid biology, where GBA3 participates in a cellular network involving NEU2 and CMAH. Overall, our data shows that the recurrent loss of *GBA3* in mammals is likely to represent an evolutionary endpoint of the relaxation of selective constraints triggered by diet-related factors.

## Introduction

Gene loss is a common evolutionary event in vertebrate genomes and an inverse relationship between the likelihood of loss and the extent of a gene’s “essentiality” in the genome of a given species has been described^1,2^. Accordingly, gene loss often involves duplicated genes, as these are frequently functionally redundant at the time of their origin. More specifically, while one of the counterparts retains its primordial function, the other can experience different fates, including pseudogenization (i.e. gene silencing by the accumulation of disruptive mutations) which is the most common outcome for the redundant copy^2^. Under these circumstances, gene pseudogenization undergoes primarily a neutral evolutionary process, since the lack of one copy of a functionally redundant gene does not result in significant fitness impairment^1^. However, over time, the intrinsic “essentiality” of a gene might change due to, among other reasons, the emergence of compensatory mechanisms that release the gene of its “essentiality” driving it to loss. A classic example of neutral gene loss in humans and other mammals is the inactivation of the gene encoding L-gulonolactone oxidase (*GULO*), the enzyme catalysing the final step in vitamin C biosynthesis, whose loss has been attributed to dietary compensation in lineages where the diet provides a steady source of ascorbic acid^3,4^. In fact, dietary specializations have been proposed to have underlain the loss of several genes in mammals, including the loss of pancreatic genes *SYCN* and *PNLIPRP1* in herbivores^5^, the loss of glucose homeostatic regulating genes *INSL5* and *RXFP4* in carnivorous mammals^5^ and bitter taste receptors gene *TAS2R* in carnivores and omnivores^6^. On the other hand, gene loss also can result in a selective advantage^1,7–9^ as, for example, seems to be the case of a 32 bp inactivating deletion in *CCR5* that was shown to provide resistance to HIV infection in humans^10^. Also the loss of the gene encoding CMP-Neu5Ac hydroxylase (CMAH), a key enzyme in the biosynthesis of the N-glycolylneuraminic acid (Neu5Gc) from its precursor N-acetylneuraminic acid (Neu5Ac), was proposed to have provided resistance to pathogens that recognize Neu5Gc^11–13^.

Although most known human pseudogenes are fixed, some cases of polymorphic pseudogenes have been identified (including the previously mentioned *CCR5*), in which both functional and non-functional forms of a gene co-segregate in human populations^14^. Because polymorphic pseudogenes are in an early phase of loss, their study offers a unique opportunity to understand how gene loss proceeds and is modelled by environmental factors (sunshine, diet, pathogens, etc.). Among the known polymorphic single copy pseudogenes in humans is *GBA3,* a gene that encodes a cytosolic β-glucosidase, also known as cytosolic β-glucosidase-like protein-1 (cBGL1)^15^, Klotho related protein (KLrP)^16^, or broad-specificity β-glucosidase (EC 3.2.1.21)^17^. In humans, the most common allele of *GBA3* corresponds to an Open Reading Frame (ORF) of 1407 bp distributed in 5 exons that encode a protein with 469 amino acids. Conversely, the pseudogenized allele results from a T > A substitution at c.1368 position (rs358231), which creates a premature stop codon (p.Y456X) and consequently a non-functional enzyme due to the lack of the terminal α-helix^18,19^.

Based on protein sequence similarities, GBA3 is currently grouped in both family 1 and 3 of glycoside hydrolases^20^ (http://www.cazy.org). Within family 1, GBA3 is closely related to the mammalian lactose phlorizin hydrolase (LCT), the enzyme that hydrolyzes lactose present in milk and whose deficiency underlies congenital lactose intolerance (MIM 223000)^21^ and acquired lactose intolerance^22,23^, the latter associated to dietary adaptations. Family 3 comprises the β-glucosylceramidase (GBA), also known as lysosomal β-glucosidase, an enzyme catalyzing the hydrolysis of glucosylceramide (GlcCer) into glucose and ceramide^24^. Impairment of *GBA* function results in Gaucher disease (MIM230800; MIM230900), a rare autosomal recessive disorder caused by lysosomal accumulation of glucosylceramide^25,26^. In contrast to *LCT* and *GBA*, the loss of *GBA3* function has until this date no associated phenotype. Due to the similarity with *GBA*, *GBA3* was initially hypothesized to act as a modifier in Gaucher disease^16,18^, but yet a study focusing the issue did not succeed to find any evidence sustaining the relationship and even revealed that the GBA3 presented low activity towards the substrates of GBA^18^.

The precise cellular role of human GBA3 is still unclear, since its specific endogenous substrate(s) is (are) also unknown. Functional characterization of the enzyme revealed a broad substrate specificity^27^, being able to hydrolyze many plant β-glycosides present in human diet such as phenolic glycosides (L-picien, salicin, arbutin), cyanogenic glycosides (amygdalin, prunasin, neolinustatin and linamarin), isoflavones (Genistin and diadzin), flavonols (spiraeoside), and flavones (apigetrin)^17,28–30^. Also GBA3 was shown to play an essential role in the deglycosylation and absorption of dietary flavonoid glycosides in human small intestine^30^. Due to GBA3 activity towards dietary glycosides and its expression in metabolic tissues such as the intestine, liver, kidney and spleen^18,27,28,30^, it was suggested that GBA3 might play an important role in the detoxification and/or biotransformation of dietary xenobiotic plant β-glycosides^28,29^.

Recently, an unsuspected role for GBA3 came to light when GBA3 was shown to bind and stabilize neuraminidase 2 (NEU2) enhancing the degradation of cytosolic sialic free *N*-glycans^31^. NEU2 catalyzes the removal of sialic acids (N-acetylneuraminic acid-Neu5Ac and N-glycolylneuraminic acid-Neu5Gc) from glycoproteins, polysaccharides and glycolipids and can efficiently catabolize Neu5Gc and its precursor Neu5Ac^32^. In a simplistic way, the metabolism of free sialoglycans involves the synthesis of CMP-Neu5Gc catalyzed by CMAH, the enzyme before mentioned whose function was lost in humans, and the catabolism of CMP-Neu5Gc and CMP-Neu5Ac by NEU2 or NEU2-GBA3.

In this work we analyse the genetic diversity of *GBA3* to obtain a comprehensive view of the extent of pseudogenization in human populations, while addressing the distribution of *GBA3* polymorphisms in the context of main human dietary preferences. In addition, we also investigate the functional status of *GBA3* in major mammalian lineages with emphasis on those presenting very distinct specialized diets, namely carnivores, omnivores and herbivores. Lastly, taking into account the newly reported role of GBA3 in the sialic acid metabolism, we evaluate the distribution of pseudogenization events in two other genes coding for key enzymes of this metabolic pathway, namely *NEU2* and *CMAH*.

## Results

### GBA3 in human populations

To understand the process of *GBA3* pseudogenization in humans, we sought to analyze the pattern of mutation accumulation in different populations. Firstly, we investigated the distribution and frequency of the premature truncation mutation rs358231 (p.Y456X) which underlies the polymorphic pseudogene status of *GBA3*. Using the information available in the 1,000 genome project database (1KGP), we collected the mean allele frequency for all super-populations (as described in 1KGP) and corresponding populations. To assess if the mean allele frequency presented in each super-population is representative of all populations included within, a Bayesian one sample t-test was conducted using the super-population mean allele frequency as test value (H0)^33,34^. Although three populations (African Caribbeans in Barbados—ACB, Gambian in Western Divisions in the Gambia—GWD and Chinese Dai in Xishuangbanna—CDX) presented frequencies not included within the 95% confidence interval, t-test analysis favored the null hypothesis over the alternative hypothesis indicating that mean allele frequency constitute good representative values for all super-populations and corresponding populations (Supplementary Table 1 and 2). Thus, further analyses were conducted using the mean values reported of each super-population. Overall, we found that the truncated allele (rs358231) is significantly (*p* < 0.05) more frequent in East Asian (0.200, or 0.210 excluding CDX population), European (0.153), American (0.108), South Asian (0.076) in comparison to African populations (0.029, or 0.025 excluding ACB and GWD populations) (Table 1, Supplementary Table 3 and 4). Homozygous individuals for the minor allele (HMA) i.e. bearing only the pseudogenized *GBA3* allele, were found in all populations except African (Table 1). In non-African populations, the observed homozygote frequency was 1.8% as a whole, peaking at 4.4% in East Asians. Of note no significant departures from the genotype distributions expected under Hardy–Weinberg equilibrium (HWE) were detected in this data set, except for American populations where the non-conformity with HWE expectations might result from their history of admixture between native and non-native populations^35^. Given rs358231 high frequency in extant human populations we next investigated human ancient genomes available, namely of Neanderthals (Altai, Vindija), Denisova and Ust'-Ishim, to assess whether any carried the pseudogenized allele, but the analysis showed that only the coding allele was present in all the ancient genomes.

**Table 1:** Frequency distribution of the 1,000 genome project (Phase 3) variants with negative effects in GBA3 and GBA.

Variant ID	Mutation	African	American	East Asian	European	South Asian	HMA
**GBA3**							
**rs358231**	**p.Y456X**^**a**^	**0.029**	**0**.**108**	**0.2**	**0**.**153**	**0**.**076**	**0.018**
**rs17612341**	p.R213P	0.001	0.007		0.029	0.011	0.001
rs182102815	p.G182S		0.004		0.003		
**rs187070546**	p.D106N		0.001		0.006	0.039	0.001
rs533876334	p.A15P					0.001	
rs544339352	p.C53S			0.001			
rs187359066	p.R82C	0.001					
rs529839966	p.T88R			0.001			
rs571805473	p.P265S	0.001					
rs538886341	p.Y281C					0.001	
rs200660617	p.V306A		0.001				
rs371662599	p.Y347X	0.001					
rs200623163	p.R389C		0.001				
rs560225618	p.K402E				0.001		
rs186578587	p.L419V			0.001			
rs371075149	p.N422K	0.001					
rs191769903	p.F433L	0.001					
rs370728701	p.V438A				0.001		
**GBA**							
rs421016	p.L483P^b^	0.002		0.001	0.012	0.002	
rs76763715	p.N409S^b^		0.001		0.002		
rs149171124	p.E427X^b^				0.001		
rs146519305	p.R534C	0.01					
rs369068553	p.V499M^b^	0.001					

HMA-Frequency of homozygous individuals for the minor allele (all 1KGP populations combined) (**a**)-Protein variant mislabeled in databases as “loss of stop codon”. (**b**)-Variants associated to Gaucher disease in ClinVar.

The high frequency of homozygotes for a non-functional allele indicates that individuals lacking *GBA3* activity are not under serious selective constraints. If *GBA3* is evolving under relaxed constraints or even neutrally, expectedly it can sustain the accumulation of further disruptive mutations, either LoF (premature truncation and frameshifts variants) or deficiency alleles (non-synonymous variants affecting GBA3 enzyme). Thus, we extended the investigation to identify additional damaging mutations in *GBA3,* collecting frequencies for ORF-disrupting mutations and non-synonymous replacements that scored both as possibly damaging by PolyPhen (score 0.7–1)^36^ and as deleterious by SIFT (score 0–0.3)^37^. For comparative purposes, an equivalent analysis was performed in *GBA*, in which mutations impairing enzymatic function cause different forms of Gaucher disease^18^.

Whereas in *GBA*, a total of 5 potentially damaging mutations were identified in the global population, all in heterozygotes, in *GBA3* a total of 18 mutations were found, of which 3 appeared in homozygosity, namely the rs358231 that defines the pseudogenized allele, the rs17612341 and the rs187070546 (Table 1). The two latter variants were less frequent than rs358231 (p.Y456X), but likewise reached higher frequencies in Eurasian populations. Moreover, to discard the hypothesis of aggregation of LoF variants within rs358231 non-functional chromosomes we inspected *GBA3* coding haplotypes. This analysis showed that the majority of disrupting alleles were not in linkage disequilibrium with rs358231, denoting independent origins of multiple inactivating haplotypes and a global pattern of relaxed constraints across *GBA3* (Supplementary Material 1A). Taking into consideration the length of the coding region of *GBA,* 1611 bp (11 exons) and of *GBA3*, 1407 bp (5 exons), the density of ORF disrupting and non-favorable amino acid replacement mutations in *GBA3* gene was clearly higher in comparison to *GBA*.

This analysis was replicated using data from a larger dataset available through the genome aggregation database (gnomAD)^38^, which, contrarily to the 1KGP database that only affords data from healthy subjects, also compiles data from cohorts of individuals with several diseases. In the gnomAD database (v 2.1.1) we retrieved a total of 43 LoF mutations in *GBA3* against only 15 in *GBA* (See Supplementary Material 1B) evidencing once more the unusual accumulation of deleterious mutations in *GBA3*. We also used gnomAD database to assess *GBA3* and *GBA* selective constraints based on LoF mutations, built on a set of statistics designed to evaluate the degree of intolerance to inactivation mutations among coding genes^38,39^. Whereas for *GBA3* the elevated numbers of LoF prompted its classification as an outlier for which constraint levels were not calculated, for *GBA* the 14 observed LoF (pLoF) mutations (one mutation failed to pass strict variant filtering criteria) were far below the expected 27.3 pLoF mutations resulting in an observed/expected (o/e) ratio of 0.51 and a LOEUF (loss-of-function observed/expected upper bound) of 0.8. The values obtained for *GBA* are not only close to the estimated genome median (0.48) and mean LOEUF (0.936) after removing top extreme genes, as they are also consistent with postulated constraint levels of an autosomal recessive gene for which selection against LoF heterozygous tends to be weak^38^.

Finally, we also investigated *GBA3* population sequence variation patterns using nucleotide diversity and Tajima’s D statistics on *GBA3* locus from the 1KGP data, and no significant departure from wide genome distribution of these statistics was detected in any population (Supplementary Material 1C).

### *GBA3* in mammals

**Figure 1:**
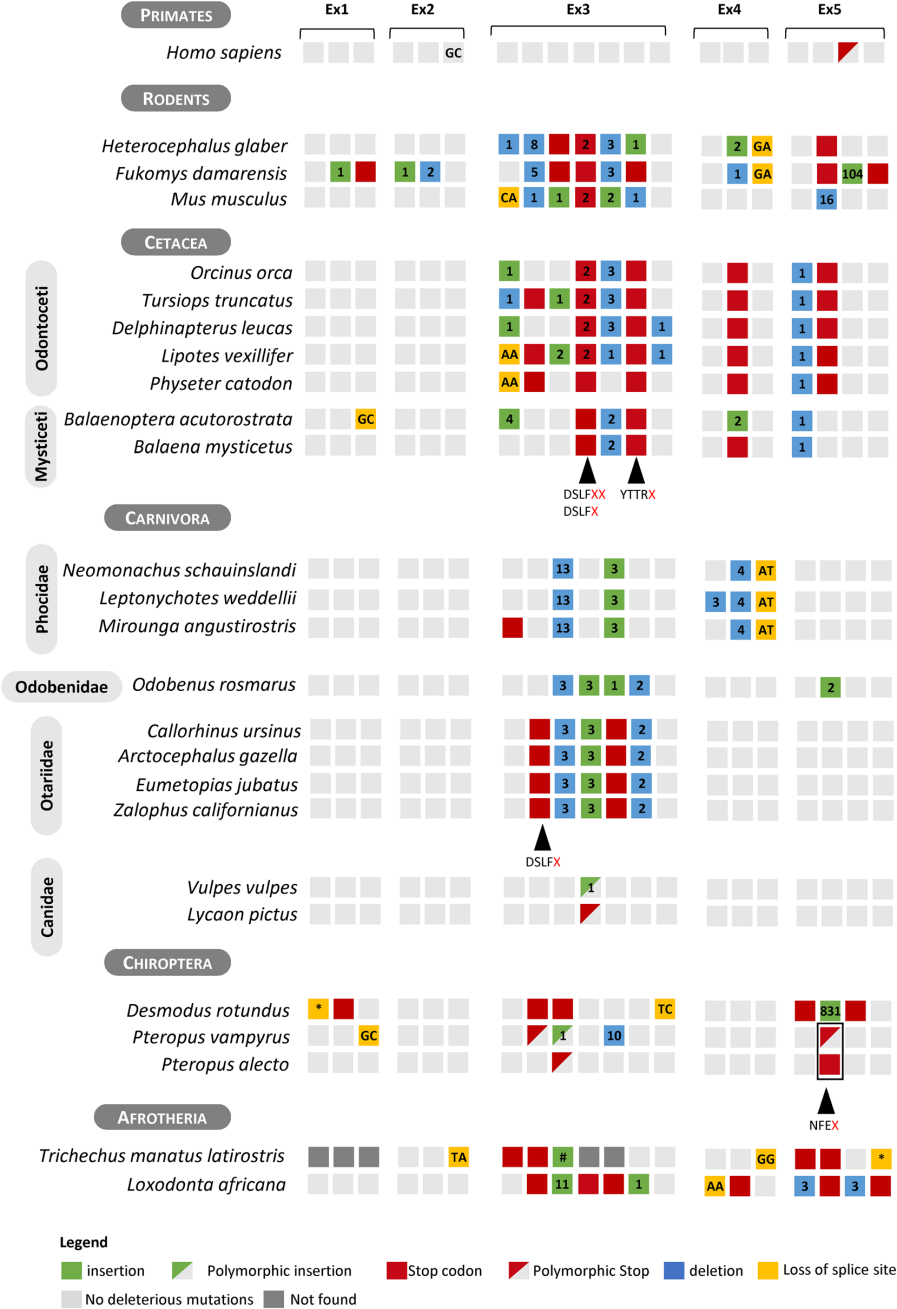
Gene annotation of *GBA3*. Schematic representation of *GBA3* identified mutations in different taxonomic groups, each cluster of grey squares represents one exon (total 5 exons). Square color code: red-stop codons; yellow-loss of splice site AG-GT or AG-GC (note: exon 2 presents conserved donor GC splice site in all species except in *Trichechus manatus latirostris*); blue—deletion and green—insertion. Number within each square indicates the number of nucleotides inserted or deleted and dark grey squares represent regions with missing data. Cross-species conserved mutations are highlighted by black arrow heads and below these 3 adjacent amino acids before the observed stop codon X are shown.

Database search in major mammalian lineages, revealed several species with: (1) *GBA3* annotations tagged as Low Quality (LQ); (2) partial *GBA3* sequences or; (3) without a *GBA3* gene annotation (Supplementary Table 5). A total of 99 mammalian species including humans were interrogated in this study. Despite a poor annotation of 4 primate species—*Aotus nancymaae*, *Callithrix jacchus, Papio anubis* and *Piliocolobus tephrosceles*, due to incomplete genome coverage, their manual annotation indicated that *GBA3* was likely to be functional in first 3 species. For *P. tephrosceles,* we were unable to validate two identified frameshift mutations and thus the pseudogeneization status of *GBA3* persisted as unconfirmed (see Supplementary Material 2 for details). In the remaining mammalian lineages, a number of LoF mutations rendering the gene inactive were detected. Herein we present the identified mutations with at least one mutation per species validated using Sequence Read Archive (SRA) data. Detailed information on mutational validation is provided in the corresponding supplementary material files. Briefly our analysis exposed numerous events of pseudogenization in multiple mammalian lineages. More specifically, in a total of 99 mammalian species investigated we identified and validated 24 species presenting *GBA3* pseudogenization. For example, in Rodentia, two mole rats (*Heterocephalus glaber* and *Fukomys damarensis*) and the mouse (*Mus musculus*), presented at least 2 premature stop codons in exon 3 in addition to other mutations (Fig. 1, and Supplementary Material 3). Gene annotation of *GBA3* in Cetacea identified various ORF damaging mutations across exons 3, 4 and 5 (Fig. 1). Interestingly, 2 premature stop codons in exon 3 (DSLF**X** and YTTR**X** Fig. 1) were found to be conserved in all cetaceans analyzed, a strong indication that *GBA3* inactivation possibly occurred in the cetacean ancestor (Supplementary Material 4).

The analysis of the *GBA3* in Carnivora revealed that species within Feliformia suborder presented an intact coding *GBA3* gene, while gene loss was observed in four Caniformia families; namely in Pinnipedia which includes two seals families (Phocidae and Otariidae) and the walrus (Odobenidae); and in Canidae (*Vulpes vulpes* and *Lycaon pictus*) (Fig. 1). All Phocidae shared a deletion of 13 nucleotides in exon 3 and all Otariidae presented a premature stop codon in exon 3 (DSLF**X** Fig. 1). As for the walrus *O. rosmarus divergens*, 3 frameshift mutations were identified and validated (Supplementary Material 5). Regarding Canidae *GBA3* annotation identified a 1 bp deletion frameshift in exon 3 in the red fox (*V. vulpes*) and a premature stop codon in exon 3 in the African wild dog (*L. pictus*). SRA validation of these mutations revealed that they were polymorphic in both species, with heterozygous specimens for the disrupted allele while the remaining samples were homozygous for an intact *GBA3* gene (Fig. 1 and Supplementary Material 5).

Still within the Carnivora families, Mustelidae and Mephitidae, we searched the unannotated genomes recently released of western spotted skunk (*Spilogale gracilis*), honey badger (*Mellivora capensis*) and giant otter (*Pteronura brasiliensis*), all presented several ORF disrupting mutations in the *GBA3* gene. However, validation of the findings was not possible given that no SRA projects were available for these species, thus evidence for the coding status of *GBA3* in these species needs to be further reinforced (Supplementary Material 5).

In Chiroptera, *GBA3* was found to be pseudogenized in the common vampire bat (*Desmodus rotundus*), in the large flying fox (*Pteropus vampyrus*) and in the black flying fox (*Pteropus alecto*)*.* Regarding the mutations identified in the common vampire bat, SRA confirmation was only possible for one vampire bat specimen. For the flying foxes SRA searches revealed that identified mutations in *P. vampyrus* were polymorphic with the exception of the 10 nucleotide deletion in exon 3, in *P. alecto* we identified a polymorphic premature stop codon in exon 3 followed by a non-polymorphic stop codon in exon 5 which is conserved with *P. vampyrus*. (Supplementary Material 6).

Finally, regarding the manatee (*Trichechus manatus latirostris*) and the African elephant (*Loxodonta africana*)*,* numerous ORF disrupting mutations were identified in both species. Although in manatee we found poor genome coverage not spanning the full *GBA3* ORF, we identified and validated two premature stop codons in exon 3 (Supplementary Material 7). Validation of the identified mutations in African elephant was performed using NCBI Trace archive data, were we confirmed the existence of 3 premature stop codons in exon 3, the loss of the canonical AG acceptor splice site in exon 4 and a premature stop codon in the same exon 4. (Supplementary Material 7).

### Selective pressures on mammalian GBA3 orthologues

**Figure 2:**
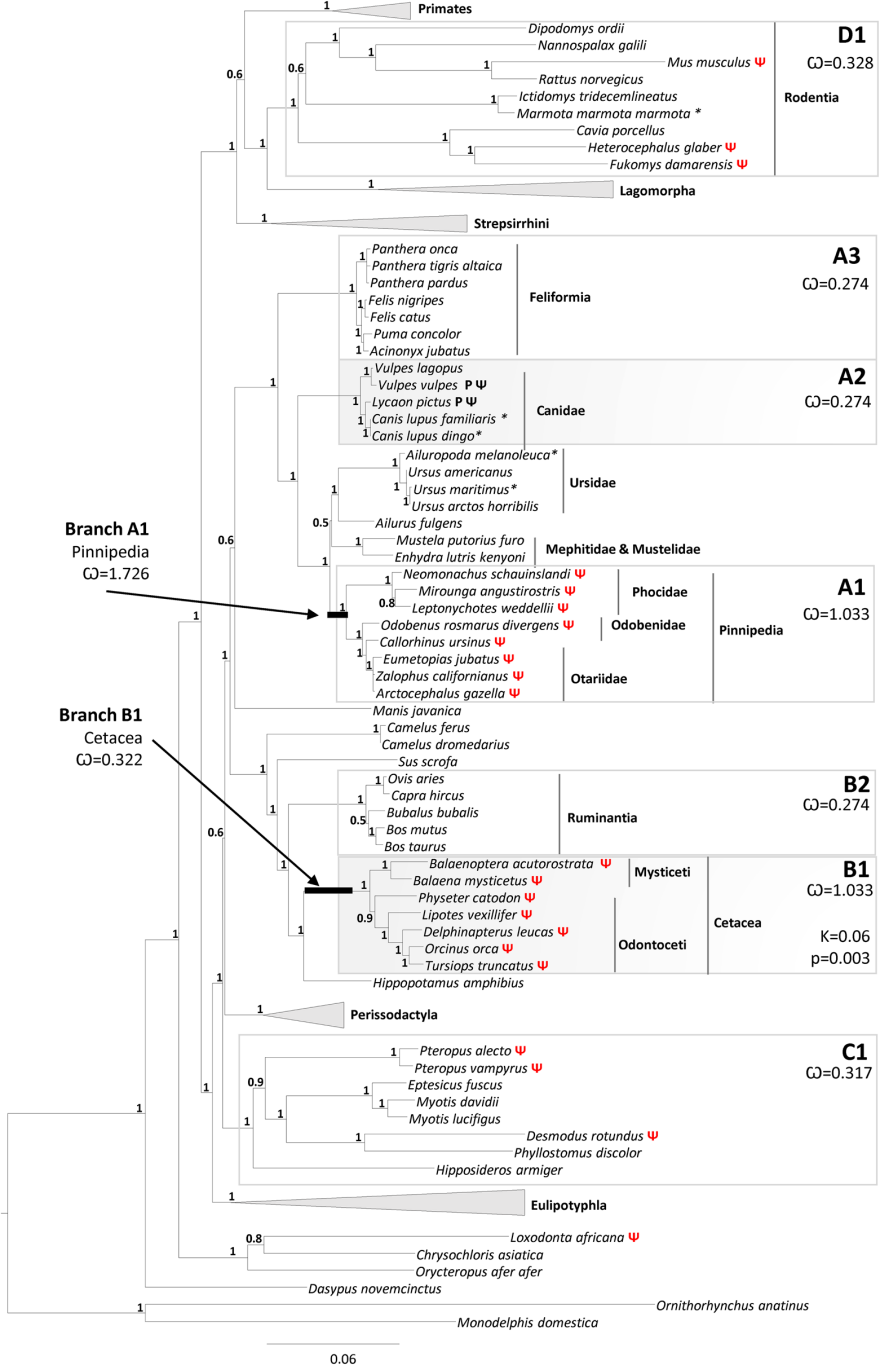
Selection and Phylogenetic analysis. Maximum likelihood phylogenetic analysis of *GBA3* nucleotide sequences, node values correspond to posterior probabilities (aBayes). Species contained in the collapsed clades are available in Supplementary Table 5. Clades analysed in CodeML are indicated by grey boxes containing corresponding clade letter A1, A2, A3, B1, B2, C1 and D1 and the omega values. In the case of Cetacea Relax analysis the K value is also indicated with the corresponding *p*-value; * indicates sequences predicted manually in unannotated genomes or poorly annotated genomes and Ѱ indicates pseudogenes.

Since shifts in selective pressures have previously been associated with events of gene loss or diversification^40–42^, we next investigated the extent of different selective pressures driving mammalian *GBA3* pseudogenization. Our investigation targeted species in which prior analysis predicted a non-coding status for *GBA3* as well as, closely related species with a coding *GBA3*, namely three Carnivora clades (Pinnipedia, Canidae and Feliformia) two Cetartiodactyla clades (Ruminantia and Cetacea), the Rodentia clade and also Chiroptera clade. A total of 14 evolutionary models were tested in CodeML that encompassed the previously mentioned seven clades (Pinnipedia-A1*,* Canidae-A2, Feliformia*-*A3*,* Cetacea*-*B1, Ruminantia-B2, Chiroptera-C1, Rodentia- D1)*,* and two branches (Pinnipedia Branch-A1, Cetacea Branch B1) (Fig. 2, and Supplementary Table 7). To identify the best fitting evolutionary model, nested likelihood tests were performed, where the alternative hypotheses was the most complex model and the simplest model was considered the null hypothesis. In the defined models, model A considers one general ω ratio for the entire phylogeny, which represents a null hypothesis for all the remaining models (Table 2). On the other hand, models B to M shift between two to five ω ratios across targeted branches and/or clades, thus allowing to test alternative scenarios of *GBA3* evolution (Table 2).

**Table 2:** Likelihood ratio test (LRT) and *p*-values.

Null hypothesis tested (H_0_)	Alternative hypothesis (H_A_)	df	LRT	*p* value
**A-one ratio** **ω**_**0**_ = **ω**_**A1**_ = **ω**_**BranchA1**_ = **ω**_**A2**_ = **ω**_**A3**_ = **ω**_**B1**_ = **ω**_**BranchB1**_ = **ω**_**C1**_ = **ω**_**D1**_	**B**-two ratio ω_0_ = ω_A2_ = ω_A3_ = ω_B1_ = ω_BranchB1_ = ω_B2_ = ω_C1_ = ω_D1;_ **ω**_**A1**_** = ω**_**BranchA1**_	1	30.4	< 0.05
	**C**-two ratio ω_0_ = ω_A1_ = ω_BranchA1_ = ω_A3_ = ω_B1_ = ω_BranchB1_ = ω_B2_ = ω_C1_ = ω_D1;_ **ω**_**A2**_	1	0.68	0.41
	**D**-two ratio ω_0_ = ω_A1_ = ω_BranchA1_ = ω_A2_ = ω_B1_ = ω_BranchB1_ = ω_B2_ = ω_C1_ = ω_D1,_ **ω**_**A3**_	1	0.36	0.55
	**E**-two ratio ω_0_ = ω_A1_ = ω_BranchA1_ = ω_A2_ = ω_A3_ = ω_B2_ = ω_C1_ = ω_D1,_ **ω**_**B1**_** = ω**_**BranchB1**_	1	35	< 0.05
	**F**-two ratio ω_0_ = ω_A1_ = ω_BranchA1_ = ω_A2_ = ω_A3_ = ω_B1_ = ω_BranchB1_ = ω_C1_ = ω_D1,_ **ω**_**B2**_	1	0.98	0.32
	**G**-two ratio ω_0_ = ω_A1_ = ω_A2_ = ω_A3_ = ω_B1_ = ω_BranchB1_ = ω_B2_ = ω_C1_ = ω_D1,_ **ω**_**BranchA1**_	1	3.56	0.06
	**H**-two ratio ω_0_ = ω_A1_ = ω_BranchA1_ = ω_A2_ = ω_A3_ = ω_B1_ = ω_B2_ = ω_C1_ = ω_D1,_ **ω**_**BranchB1**_	1	0.06	0.81
	**I-** two ratio ω_0_ = ω_A1_ = ω_BranchA1_ = ω_A2_ = ω_A3_ = ω_B1_ = ω_B2_ = ω_BranchB1_ = ω_D1,_ **ω**_**C1**_	1	0.47	0.49
	**J-** two ratio ω_0_ = ω_A1_ = ω_BranchA1_ = ω_A2_ = ω_A3_ = ω_B1_ = ω_B2_ = ω_BranchB1_ = ω_C1,_ **ω**_**D1**_	1	1.83	0.18
	**K**-two ratio ω_0_ = ω_A2_ = ω_A3_ = ω_B2_ = ω_C1_ = ω_D1,_ **ω**_**A1**_** = ω**_**BranchA1**_** = ω**_**B1**_** = ω**_**BranchB1**_	1	67.07	< 0.05
	**L**-three ratio ω_0_ = ω_A2_ = ω_A3_ = ω_B2_ = ω_C1_ = ω_D1,_ **ω**_**A1**_** = ω**_**BranchA1**,_ ω_B1_ = ω_BranchB1_	2	67.76	< 0.05
	**M**-four ratio ω_0_ = ω_A2_ = ω_A3_ = ω_B2_ = ω_C1_ = ω_D1,_ **ω**_**A1=**_**ω**_**B1**,_ ω_BranchB1**,**_** ω**_**BranchA1**_	**3**	**74.62**	** < 0.05**
	**N-**five ratio ω_0_ = ω_A2_ = ω_A3_ = ω_B2_ = ω_C1_ = ω_D1,_ **ω**_**A1**,_ ω_BranchA1,_ **ω**_**B1**,_ ω_BranchB1_	4	74.62	< 0.05
**K**-two ratio	**L** -three ratio	1	0.70	0.40
**K**-two ratio	**M** -four ratio	**2**	**7.56**	** < 0.05**
**K**-two ratio	**N -**five ratio	3	7.56	0.06
**M**-four ratio	**N -**five ratio	1	0.00	1.00

*P*-values < 0.05 were considered significant (Clades A1-Pinnipedia, A2-Canidae, A3 Feliformia, B1 Cetacea, B2 Cetacea, C1 Chiroptera, D1 Rodentia and ancestral branches Branch A1- Pinnipedia and Branch B1-Cetacea).

Considering the two ratio models, model K, which contrasts the evolution of Pinnipedia and Cetacea (ω_A1_ = ω_Branch A1_ = ω_B1_ = ω_Branch B1_) with the remaining 3 clades Canidae*,* Feliformia, Ruminantia, Chiroptera and Rodentia (ω_A2_ = ω_A3_ = ω_B2_ = ω_C1_ = ω_D1_), presented the highest LRT value (67.07). This result indicates model K is the most likely of the two ratio models tested. Next, more complex evolutionary hypothesis were evaluated aiming to distinguish the mode of selection pressure between the clades and branches. This set of analyses revealed that the evolutionary model M with four independent rates (ω_0_ = ω_A2_ = ω_A3_ = ω_B2_ = ω_C1_ = ω_D1,_ ω_A1=_ ω_B1,_ ω_BranchB1,_ ω_BranchA1_) was the best supported model, given it rejected all simpler models (A and L) but could not be rejected by a more complex five ratio model N (Supplementary Table 7 and 8). Model M considers that Canidae, Feliformia Ruminantia, Chiroptera and Rodentia clades (ω_A2_ = ω_A3_ = ω_B2_ = ω_C1_ = ω_D1_), all comprising species with coding *GBA3*, do not significant shift from each other and ω_0_, while the clades including species with pseudogenized *GBA3*, namely Cetacea (ω_B1_ = 1.033) and Pinnipedia (ω_A1_ = 1.033), shifted significantly from ω_0_. Additionally, Pinnipedia branch (ω_branch A1_ = 1.726) and Cetacea branch (ω_branch B1_ = 0.322) showed significant shifts from ω_0_, suggesting earlier changes in the selective pressure towards diversifying and/or relaxed selection in both their ancestors’ lineages (Fig. 2).

To further dissect these shifts in selective pressures, a RELAX analysis targeting both the Pinnipedia and Cetacea clades was performed under the assumption that relaxed selection is often associated with the pseudogenization process^42^. A significant (*p* = 0.003) relaxation (K = 0.06) of the selective pressures was identified in the Cetacea clade, while for the Pinnipedia clade no significant selection relaxation or intensification was found.

### The evolutionary trajectories of *NEU2 and CMAH*

**Figure 3:**
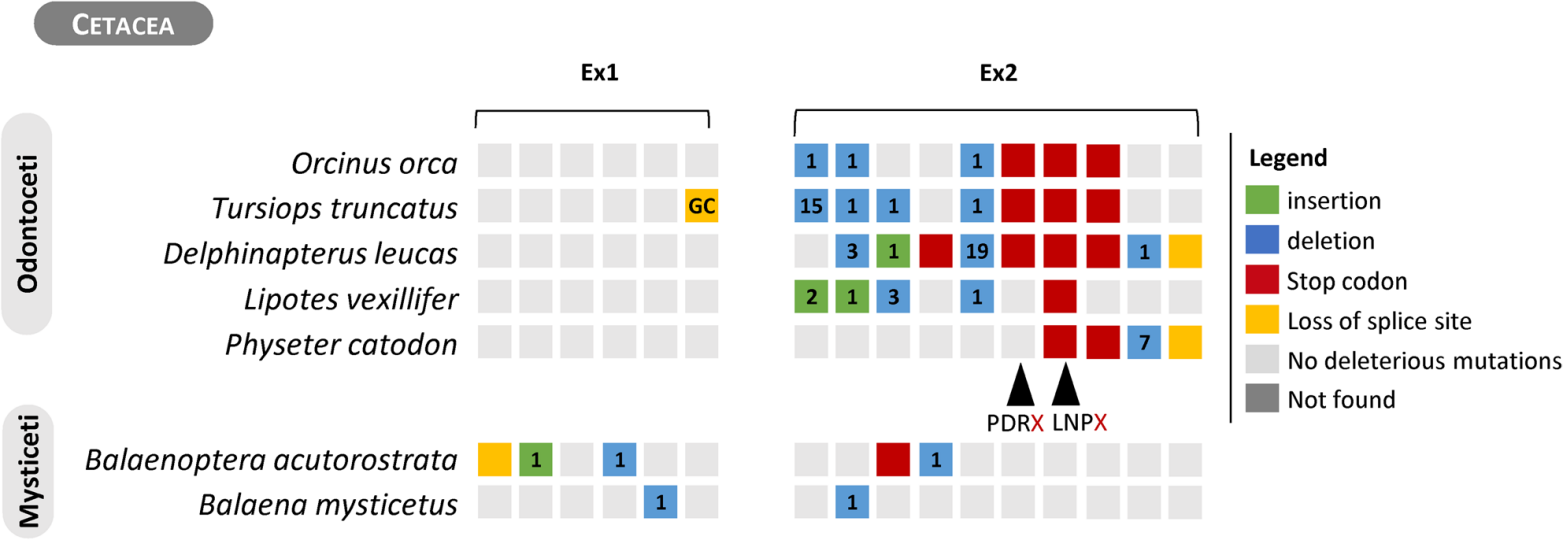
Gene annotation of *NEU2*. Schematic representation of the *NEU2* gene and identified mutations, each group of grey squares represents one exon (total 2 exons). Square color code: red—stop codons; yellow—loss of canonical AG-GT splice site; blue—deletion and green—insertion. Number within each square indicates the number of nucleotides inserted or deleted and dark grey squares represent regions with missing data. Cross-species conserved mutations are highlighted by black arrow heads and below these 3 three adjacent amino acids before the observed stop codon X are shown.

The recently documented involvement of GBA3 in the structural stabilization of the enzyme NEU2^43^ led us to investigate the status of *NEU2* in species where *GBA3* was predicted to have been lost. *NEU2* annotation revealed the presence of active genes in the majority of the mammalian species analyzed, yet, signs of pseudogenization were detected in the Cetacea and in the rodent *F. damarensis* (Supplementary Table 6). For these mammals the NCBI database search revealed several LQ annotation thus *NEU2* genomic sequences were collected and submitted to manual annotation revealing a number of ORF disrupting mutations (Fig. 3). More specifically, two stop codons were found in exon 2, PDR**X** and LNP**X** (both validated in SRA data, see Supplementary Material 8), with the first being shared by *O. orca, T. truncatus* and *D. leucas*, and the second being also present in the latter 3 species plus in *L. vexillifer* and *P. catadon*. ORF disrupting mutations were also identified in the Mysticeti species *B. acutorostrata* and *B. mysticetus*, which were also validated using independent SRA data (Supplementary Material 8). Regarding *F. damarensis* manual gene annotation revealed a 489 bp insertion in exon 2 followed by a premature stop codon. While SRA searches confirmed the presence of a polymorphic premature stop in exon 2, it did not validate the insertion, being most probably a result of poor genome assembly, thus NEU2 seemingly constitutes a polymorphic pseudogene in this species (Supplementary Material 9).

**Figure 4:**
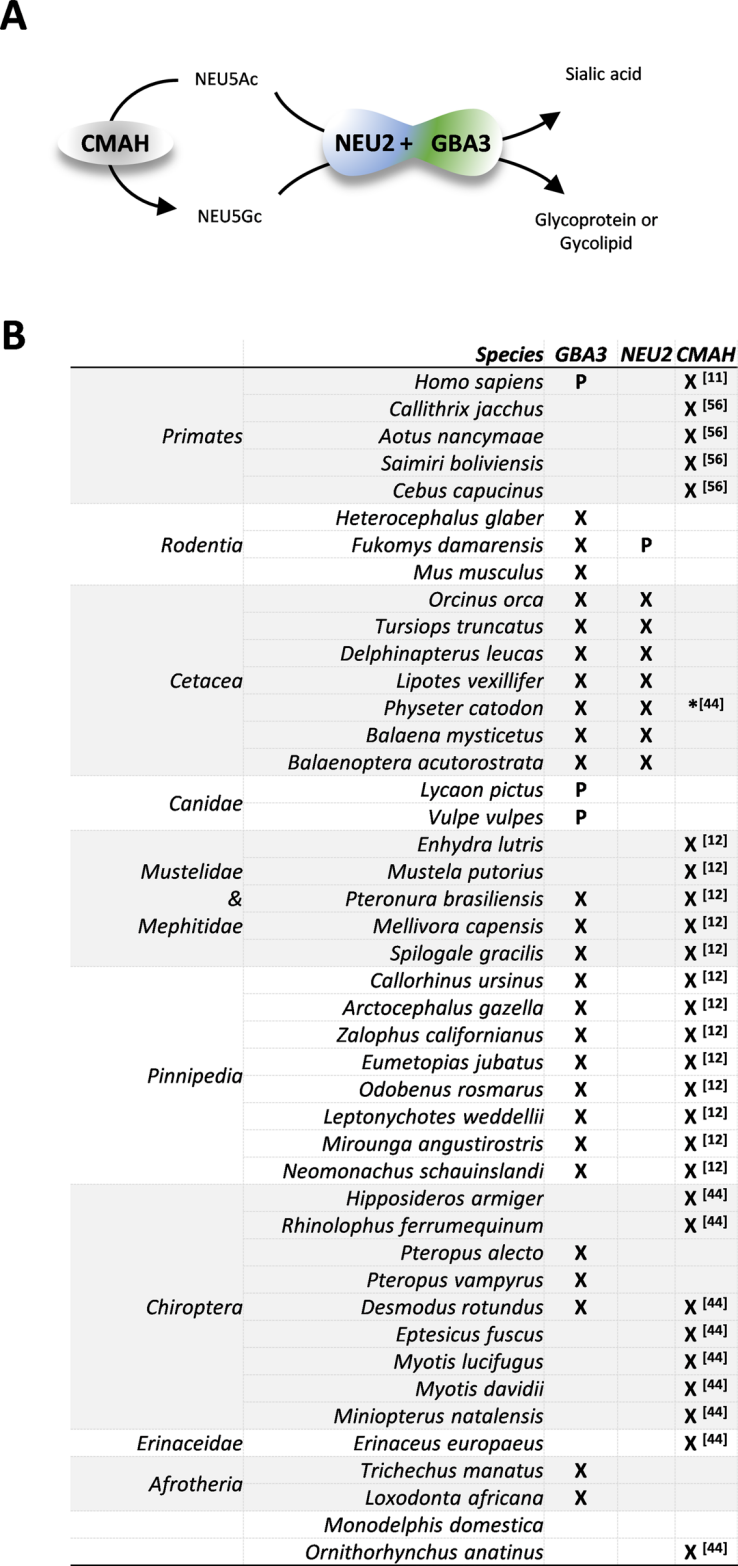
(**A**) Schematic illustration of the roles proposed for GBA3 and NEU2 in sialic metabolism. (**B**) Coding status of *GBA3*, *NEU2* and *CMAH* in mammals, P indicates polymorphic pseudogene, X specifies gene loss, * coding ORF previously reported as lost.

Given the involvement of both NEU2 and CMAH in the sialic acid metabolism (Fig. 4A), it appeared relevant to cross the *NEU2* and *GBA3* data obtained in this study with previously published information pertaining *CMAH*. In Fig. 4B is summarized the information concerning species where at least one of the 3 genes *GBA3, NEU2* or *CMAH* was predicted to be inactive. Concerning the coding status of *CMAH* in the sperm whale (*P. catodon*), Peri et al.(2017)^44^ predicted this gene as being pseudogenized based on the lack of exon 5 in the genomic sequence then available. Meanwhile, a new sperm whale genome assembly (GCF_002837175.2) was released, with an intact *CMAH* ORF (XM_028479288.1), containing thus exon 5, an observation that entailed the revision of the coding status of *CMAH* in *P. catodon* from putative pseudogene, to putative active gene or at least putative polymorphic pseudogene^44^.

## Discussion

The elucidation of the still obscure physiological function of *GBA3* has been greatly hindered by the lack of knowledge on any specific endogenous substrate of the enzyme, as well as by the fact that the loss of its functionality does not result in any phenotypic consequences recognized up to now^18^. In humans, *GBA3* is a polymorphic pseudogene due to variation at position rs358231 that leads to an inactivating allele rather widespread and common in most populations. Here we show that in addition to this, *GBA3* accumulates many other functionally disruptive variants that as a whole further increase the degree of *GBA3* inactivation in human populations. This means that *GBA3* pseudogenization in humans is more extensive and genetically heterogeneous than initially anticipated. Noteworthy, a previous study addressing human genetic variations that could be associated with adaptations to diet revealed a significant correlation between various *GBA3* variants and populations with diets rich in roots and tubers^45^. The finding seems reconcilable with the catalytic activity of GBA3 towards many xenobiotic plant glycosides present in human diet, such as the toxic cyanogens found in high amounts in the edible parts of cassava and cocoyam, two staple foods in African populations^46^, or the quercetin found in many plants and fruits^27,29^. The populations addressed in the work of Hancock et al.^45^, encompassed distinct subsistence modes, data that unfortunably is not available for the 1KGP populations for which only geographical origin is provided. However, according to the dietary habits available from the Food and Agriculture Organization of the United Nations (FAO, www.FAO.org), in Africa many populations rely on roots and tubers as a significant part of the staple diet together with cereals, fruits, nuts, pulses, vegetables whereas meat and fish represent a minority component of diet. By contrast, in Asian and European populations, roots and tubers are essentially absent from the main diet which instead is rich in cereals, meat, fish, milk, eggs and other dairy products (FAO)^47^. In this framework, it is striking having here observed an overlap in Eurasia between the highest frequency of *GBA3* loss and a main dietary source of meat or fish, while in Africa the lowest frequency of *GBA3* inactive alleles meets the general trend in diets higher in glycoside-rich foods and poorer in meat or fish. Hence, and similarly to the widely assumed for the loss of *GULO*^3^, the propensity to *GBA3* inactivation in humans may well have been related to dietary fluctuations during evolution of the human lineage, following the evolutionary tenet “use it or lose it”.

The hypothesis that *GBA3* evolution was associated with dietary preferences, was further explored by analyzing the coding status of *GBA3* and selection pressures in mammalian species covering herbivores, carnivores and omnivores. Evidence emerged that *GBA3* was recurrently inactivated during mammalian evolution, pointing to at least 9 independent events of *GBA3* loss, namely in the cetacean ancestral, in all Pinnipedia analyzed in the work (Phocidae, Odobenidae, Otariidae), and additionally in three rodents, three Chiroptera, in the African elephant (*L. africana*) and in the manatee (*T. latirostris*). A fine analysis of the selective pressures in the clades where *GBA3* was inactivated and in the corresponding sister clades, showed an accelerated/relaxed selection in the ancestral branch of Pinnipedia and Cetacea, indicating that *GBA3* was under less selective constraint in the ancestral of these lineages. This shift of selective pressure is further sustained in the extant cetaceans and pinnipeds, supporting the fixation of the identified LoF mutations in these lineages (Fig. 2). Interestingly both cetaceans and pinnipeds are aquatic mammals with a specialized carnivorous diet, ranging from krill to fish, cephalopods and crustaceans^48^. The identification of two fixed premature stop codons shared by all cetacean species analyzed provides a strong indication that *GBA3* inactivation took place in the cetacean ancestor approximately 50 Mya, a finding that is consistent with a major dietary change thought to have occurred during the ancestral cetacean transition to aquatic environments^49^. Furthermore, the loss of xenobiotic glycosidase *GBA3* in Cetacea is in line with a previous study reporting that cetaceans have lost key players involved in xenobiotic metabolism, namely xenobiotic receptors NR1I3 and NR1I2, which was also associated to the low or absent content of plant derived xenobiotics in their diet^5^.

*GBA3* was also found to be pseudogenized in three Chiroptera species namely the in large flying fox (*P. vampyrus*) the black flying fox (*P. alecto*) and in the vampire bat (*D. rotundus*) the latter species with a very special diet feeding solely of blood, hematophagy^50^. The exclusively blood-based diet in the vampire bat has already been related to the loss of genes involved in taste and olfactory perception as well as genes involved in xenobiotic and immune response such as *UGT2B17, CTSG, CCL2* and *KLRB1*^51^. Likewise, the flying foxes are also known to have a highly specialized diet consisting essentially of fruit and fruit juices, which has been associated with the loss of several genes^7^. Similarly, the loss of *GBA3* in these species may have also arose as another evolutionary response to their extreme diet.

Lastly, the few remaining species predicted to have lost *GBA3-*Rodentia *F. damarensis, H. glaber* and *M. musculus;* the manatee *T. latirostris*, and the African elephant *L. africana* all have a plant based or omnivorous diet. The two mole rats (*F. damarensis, H. glaber*) are noteworthy given that the main dietary components of these subterranean species are tubers, roots and other underground plant storage organs containing cyanogenic glucosides, alkaloids and phenols^52,53^. Interestingly a previous study identified numerous positively selected genes, some of which associated with enhanced response to xenobiotic *stimulus* and immune response^52^. Furthermore, mole rats were shown to possess a highly specialized gut microbiome^54,55^. Possibly, those and other still unknown mechanisms were essential to allow mole rats to deal with a xenobiotic-rich diet.

The discovery of a combined role between GBA3 and NEU2 in the catabolism of sialoglycans^43^ (Fig. 4A), prompted us to analyze the phylogenetic distribution of *NEU2* in mammals. Manual gene annotation of *NEU2* revealed that only cetaceans presented fixed ORF disrupting mutations in this gene (Fig. 3), while the rodent *F. damarensis* showed a polymorphic pseudogene status. *GBA3* was also found to be non-functional in Cetacea, but it seems that the two genes have not undergone a process of co-elimination. Indeed, the loss of *GBA3* arose from a single event in the cetacean ancestor, whereas the inactivation of *NEU2* was apparently more recent, resulting from at least 3 independent events, one in the Odontoceti ancestral and the others in the two Mysticeti lineages. Still, the occurrence of such concomitant loss of the two genes in all cetacean lineages is remarkable, particularly when no similar signature emerged from the remaining mammalian groups. So, other mechanisms might exist in Cetacea to compensate the compromised catabolism of cytosolic free sialoglycans due to the loss of *NEU2*.

Conversely and more similarly to the *GBA3* inactivation pattern*, CMAH* appears to have been disrupted several times during mammalian evolution namely in humans, platyrrhines^56^, in three Chiroptera families, in the ancestors of Pinnipedia and mustelids, among others^12^ (Fig. 4B), indicating that both genes were often dispensed during mammalian evolution. At present, the role of *GBA3* in sialic acid biology is far from being deciphered. Even assuming a minor role, taking into account the gene repertoire involving *GBA3*, *NEU2* and *CMAH* in different mammals, our findings suggest that many evolutionary solutions can cope with the specificities of the sialic acid biology typical from each lineage, specificities that possibly might influence the mammalian interactions with pathogens.

Altogether our findings suggest that the evolution of *GBA3* in mammals may have been shaped by the dietary preferences in different lineages, turning this gene more prone to a relaxed evolution when its role was less constrained by dietary xenobiotic β-glycosides. We showed that *GBA3* inactivation recurred at least nine independent times during mammalian evolution, most pseudogenization events took place in lineages with a carnivorous diet, in keeping with the assumption that *GBA3* plays an important role in the biotransformation and/or detoxification of plant β-glycosides^28^. On the other hand *GBA3* loss was also observed in lineages with an omnivorous or strict herbivorous diet which may be underlined by unknown species specific adaptations. In humans, the pseudogenization of *GBA3* seems to be in route. In line with the diet model, the distribution of inactivating alleles in extant human populations may have been connected with a dietary transition from roots and tubers in Africa, to another regime enriched in meat, fish and dairy products in Eurasia. It can be argued that such pattern of allele distribution could well have derived from the dispersal out of Africa of modern humans, during which non-African populations experienced severe bottlenecks^57^. Nonetheless, the explanation is difficult to reconcile with the fact that not only one but instead distinct *GBA3* loss of function alleles, all display the highest frequencies in Eurasian populations. Regardless of the underlying pressures, the disruption of *GBA3* function is seemingly very well tolerated in humans and other mammals pinpointing an ongoing neutral process of pseudogenization.

## Methods

### Sequence retrieval

*GBA3* and *NEU2* coding nucleotide sequences from all major mammalian lineages were collected from Ensembl release 96^58^ and Genbank^59^. Sequence collection included all major mammalian lineages and was performed with blastn and blastp searches using human coding *GBA3* (NM_020973.4) and *NEU2* (NM_005383.2) nucleotide sequences as query (accession numbers available in Supplementary Table 5 and Supplementary Table 6). The collected nucleotide sequences were uploaded into Geneious R7.1.9 (https://www.geneious.com), aligned with MAFFT plug in^60^ and manually curated by removing the 5´and 3´untranslated regions. Whenever only partial or poorly aligned *GBA3* sequences were available, the corresponding genomic sequences were collected from the matching genome assemblies available at NCBI and manually annotated.

### Analysis of GBA3 heterogeneity

Intraspecific variation analysis was performed using data from the 1,000 Genome Project (1KGP) Phase 3 on the GRCh38^35^ including the following populations: African (African Caribbean in Barbados, African ancestry in the Southwest US, Esan in Nigeria, Gambian in Western Division, Luhya in Webuye, Mende in Sierra Leone, Yoruba in Ibadan); American (Colombian in Medellin, Mexican ancestry in Los Angeles, Peruvian in Lima, Puerto Rican in Puerto Rico); East Asian (Chinese Dai in Xishuangbabba, Han Chinese in Beijing, Southern Han Chinese, Japanese in Tokyo, Kinh in Ho Chi Minh City); European (Utah residents with Northern and western European ancestry, Finnish in Finland, British in England and Scotland, Iberian populations in Spain, Tuscany in Italy) and South Asian (Bengali in Bangladesh, Gujarati Indian in Houston, Indian Telugu in the UK, Punjabi in Lahore, Sri Lankan Tamil in the UK). This data was collected in Ensembl release 96 using the variation resource pipeline^58,61^ and information was retrieved only for exonic loss-of-function (LoF) variants namely missense, frameshift and truncation mutations. Frequency of biallelic variants that simultaneously showed a PolyPhen score between 0.7–1^36^ and a SIFT score within the range of 0–0.3^37^ (most likely to be deleterious) were collected for *GBA3* and *GBA*.

Previously identified LoF variants were also investigated in ancient genome data in Neanderthals individuals (Altai, Vindija), Denisova and Ust'-Ishim, this data was collected using the JBrowse available at https://bioinf.eva.mpg.de/jbrowse. The diversity of the genomic *locus* containing *GBA3* was evaluated in all populations using Tajima's D statistics^62^ and nucleotide diversity (Pi)^63^ available in POPHUMAN genome browser^64^. Tajima's D statistics and Pi values were calculated in sliding windows of 100 kb covering the genomic region of *GBA3* in all populations from the 1KGP phase 3. Hardy and Weinberg Equilibrium (HWE) P-values for genotypic distribution of the collected data was determined via the Court lab-HW calculator^65^. Bayesian one sample test of population mean allele frequencies was conducted in JASP V0.12.2 (https://jasp-stats.org/)^33,34^. Multiple comparison one way ANOVA was calculated in GraphPad Prism version 7.00 for windows (GraphPad Software, La Jolla California USA, www.graphpad.com).

### *GBA3* and *NEU2* gene annotation

*GBA3* and *NEU2* manual gene annotation was performed for species presenting partial or poorly aligned sequences, and/or for annotations tagged as Low Quality (LQ) or no gene annotation. In these cases, the corresponding genomic region flanked by the neighbouring genes *ADGRA3* and *PPARGC1* in the case of *GBA3*, and *INPP5D* and *NGEF* in the case of *NEU2* were collected for manual gene prediction. The collected genomic sequences were uploaded into Geneious R7.1.9 and the gene sequence was manually predicted as described in^40^ using human *GBA3* and *NEU2* coding sequences as reference. Briefly, human *GBA3* and *NEU2* exons were mapped to the corresponding genomic sequences, next aligned regions were manually inspected to identify ORF disrupting mutations (frameshifts, premature stop codon, loss of canonical splice sites). The identified ORF disrupting mutations (one per species) were validated by searching at least two independent SRA projects (when available) of the corresponding species. When no ORF mutation was identified, the predicted coding sequence was extracted and included in the phylogenetic and selection analysis.

### Phylogenetic and selection analysis

For the phylogenetic and selection analysis, coding and predicted non-coding sequences of *GBA3* were uploaded into Geneious R7.1.9 and aligned using translation align option. Sequence alignment was inspected to remove columns containing 90% gaps, as well as the final stop codon. Additionally, premature stop codons and frameshift mutations were removed in the predicted non-coding *GBA3* sequences with the deletion of the corresponding codon. Final sequence alignment contained 97 sequences and 1,410 positions. To establish orthology of the collected *GBA3* sequences (coding and non-coding) phylogenetic tree was calculated by submitting sequence alignment to PhyML3.0 server^66^ and the maximum likelihood phylogenetic analysis was performed with best sequence evolutionary model GTR + G + I determined using smart model selection^67^ and branch support calculated using aBayes algorithm^68^. The resulting phylogenetic tree was visualized using FigTreev1.3.1 (https://tree.bio.ed.ac.uk/software/figtree/).

For the selection analysis, a phylogenetic tree was calculated using the RAxML^69^, which was run using the GTR model with the remaining parameters set to default. Both multiple sequence alignment and tree were next submitted to CodeML for selection analysis, selective pressures were estimated in seven mammalian clades (Pinnipedia-A1*,* Canidae-A2*,* Feliformia*-*A3*,* Cetacea*-*B1 Ruminantia-B2, Rodentia-C1 and Chiroptera D1) and two branches (Pinnipedia Branch- A1 and Cetacea Branch B1) using the branch models from CodeML implemented in PAML v4.9i^70^. Tested models considered one or more Ѡ (dN/dS) rates, in the selected clades and/or branches (Supplementary Table 7).

The log Likelihood values (lnL) estimated by CodeML were used to perform a likelihood ratio test (LRT = 2 × (lnL_1 _− lnL_0_), statistical significance of the P value was obtained by comparing the LRT value against X^2^ distribution, where the degree of freedom was the difference of number of parameters between the null and alternative hypothesis. P values below 0.05 were considered significant.

Clades showing significant shifts in Ѡ rates in CodeML were further analyzed in RELAX^42^ to determine the direction of natural selection (relaxed or intensified). RELAX analysis was conducted on the Datamonkey server 2.0^71^, using the previous sequence alignment. Clades presenting significant shifts in Ѡ rates in CodeML were selected as foreground in RELAX and compared against the remaining sequences which were defined as background.

## Supplementary information

Supplementary Tables

Supplementary Material
