# Supplementary material for: GBA3: a polymorphic pseudogene in humans that experienced repeated gene loss during mammalian evolution
Source: Sci Rep. 2020 Jul 14;10:11565. doi: 10.1038/s41598-020-68106-y (PMC7360587; doi:10.1038/s41598-020-68106-y)
Supplement: Supplementary file 1 — Supplementary Tables [file 41598_2020_68106_MOESM1_ESM.pdf]

SUPPLEMENTARY TABLES

GBA3: a polymorphic pseudogene in humans that experienced repeated gene loss during mammalian evolution

Lopes-Marques, Monica[1,2,3ł]; Serrano, Catarina [1,2,3]; Cardoso, Ana R. [1,2,3]; Salazar, Renato[1,2]; Seixas, Susana[1,2], Amorim, António[1,2,3]; Azevedo, Luisa[1,2,3*] Prata, Maria J[1,2,3*]

[1]i3S- Instituto de Investigação e Inovação em Saúde, Universidade do Porto, Population Genetics and Evolution Group, Rua Alfredo Allen 208, 4200-135 Porto, Portugal

[2]IPATIMUP-Institute of Molecular Pathology and Immunology, University of Porto, Rua Júlio Amaral de Carvalho 45, 4200-135 Porto, Portugal

[3]Department of Biology, Faculty of Sciences, University of Porto, Rua do Campo Alegre, s/n, 4169-007 Porto, Portugal

Supplementary Table 1: rs358231 minor allele frequency distribution in all populations analyzed. ACB- African Caribbeans in Barbados; ASW- Americans of African Ancestry in SW USA; ESN-Esan in Nigeria; GWD- Gambian in Western Divisions in the Gambia; LWK-Luhya in Webuye, Kenya; MSL-Mende in Sierra Leone; YRI-Yoruba in Ibadan, Nigeria; CLM-Colombians from Medellin, Colombia; MXL-Mexican Ancestry from Los Angeles USA; PEL-Peruvians from Lima, Peru; PUR-Puerto Ricans from Puerto Rico; CDX-Chinese Dai in Xishuangbanna, China; CHB-Han Chinese in Beijing, China; CHS-Southern Han Chinese; JPT-Japanese in Tokyo, Japan; KHV-Kinh in Ho Chi Minh City, Vietnam; CEU- Utah Residents (CEPH) with Northern and Western European Ancestry; FIN-Finnish in Finland; GBR-British in England and Scotland; IBS-Iberian Population in Spain; TSI- Tuscany in Italia; BEB-Bengali from Bangladesh; GIH-Gujarati Indian from Houston, Texas; ITU-Indian Telugu from the UK; PJL-Punjabi from Lahore, Pakistan; STU-Sri Lankan Tamil from the UK.

**African**		**American**		**East Asian**		**European**		**South Asian**	
**ACB**	0,062	**CLM**	0,144	**CDX**	0,145	**CEU**	0,136	**BEB**	0,105
**ASW**	0,033	**MXL**	0,055	**CHB**	0,194	**FIN**	0,162	**GIH**	0,053
**ESN**	0,02	**PEL**	0,129	**CHS**	0,219	**GBR**	0,126	**ITU**	0,083
**GWD**	0,013	**PUR**	0,091	**JPT**	0,221	**IBS**	0,173	**PJL**	0,089
**LWK**	0,03			**KHV**	0,217	**TSI**	0,164	**STU**	0,054
**MSL**	0,018								
**YRI**	0,028								
**Mean**	0,029		0,105		0,199		0,152		0,077

Supplementary Table 2: Bayesian One Sample T-Test, using the Two-sided alternative hypothesis that the super-population mean is not equal to the test value (H0). **a**- Average frequency of all populations from 1KGP, **b**-Average frequency of all populations within the 95% confidence interval, N- Number of populations included in the test, SD standard deviation, SE standard error of the mean, BF_{01} and BF_{10} Bayes factors of likelihood of H0 and H1 respectively, ACB- African Caribbeans in Barbados GWD- Gambian in Western Divisions in the Gambia and CDX Chinese Dai in Xishuangbanna.

								**95% Confidence Interval**		
**Population**	**N**	**Mean** (H0)	**SD**	**SE**	**BF_{01}**	**BF_{10}**	**error %**	**Lower**	**Upper**	Outlier sub populations
**African**	7[a]	0.029[a]	0.016[a]	0.006[a]	2.831[a]	0.353[a]	6.219e -6[a]	0.014[a]	0.044[a]	ACB - 0.062<br>GWD - 0.013
	5[b]	0.026[b]	0.006[b]	0.003[b]	2.423[b]	0.410[b]	1.146 e-5[b]	0.018[b]	0.34[b]	-
**American**	4	0.105	0.040	0.020	2.338	0.428	2.606e -7	0.041	0.168	-
**East Asian**	5[a]	0.199[a]	0.032[a]	0.014[a]	2.516[a]	0.397[a]	6.962e -6[a]	0.159[a]	0.239[a]	CDX - 0.0145
	4[b]	0.213[b]	0.013[b]	0.006[b]	2.162[b]	0.462[b]	5.213e -5[b]	0.193[b]	0.233[b]	-
**European**	5	0.152	0.020	0.009	2.516	0.397	6.916e -6	0.127	0.177	-
**South Asian**	5	0.077	0.023	0.010	2.516	0.397	6.942e -6	0.049	0.105	-

Supplementary Table 3: Multiple comparison One Way ANOVA of rs358231 minor allele frequency in all super-populations

Tukeys multiple comparision test	Mean Diff.	95% CI of Diff	p-value
**AFR vs. AMR**	-0.07561	-0.1241 to - 0.02707	0.012 **
**AFR vs. EAS**	-0.1701	-0.2154 to -0.1247	<0.0001 ****
**AFR vs. EUR**	-0.1231	-0.1684 to -0.07771	<0.0001 ****
**AFR vs. SAS**	-0.04766	-0.093 to -0.002311	0.0364 *
**AMR vs. EAS**	-0.09445	-0.1464 to -0.045	0.0002 ***
**AMR vs. EUR**	-0.04745	-0.0994 to 0.004501	0.0843 ns
**AMR vs. SAS**	0.02795	-0.024 to 0.0799	0.5119 ns
**EAS vs. EUR**	0.047	-0.00198 to 0.09598	0.0640 ns
**EAS vs. SAS**	0.1224	0.07342 to 0.1714	<0.0001 ****
**EUR vs. SAS**	0.0754	0.02642 to 0.1244	0.0014 **

Supplementary Table 4: Multiple comparison One Way ANOVA of rs358231 minor allele frequency in all super-populations, except populations ACB, GWD, and CDX.

Tukeys multiple comparision test	Mean Diff.	95% CI of Diff	p-value
**AFR vs. AMR**	-0.07895	-0.1313 to -0.0266	0.0019 **
**AFR vs. EAS**	-0.1734	-0.2228 to -0.124	<0.0001 ****
**AFR vs. EUR**	-0.1264	-0.1758 to -0.07704	<0.0001 ****
**AFR vs. SAS**	-0.051	-0.1004 to -0.001643	0.0408 *
**AMR vs. EAS**	-0.09445	-0.1468 to -0.0421	0.0003 ***
**AMR vs. EUR**	-0.04745	-0.0998 to 0.004901	0.0870 ns
**AMR vs. SAS**	0.02795	-0.0244 to 0.0803	0.5118 ns
**EAS vs. EUR**	0.047	-0.002357 to 0.09636	0.0666 ns
**EAS vs. SAS**	0.1224	0.07304 to 0.1718	<0.0001 ****
**EUR vs. SAS**	0.0754	0.02604 to 0.1248	0.0016 **

Graph plots from supplementary table 3 and 4 - A- Multiple comparison One Way ANOVA of rs358231 minor allele frequency in all super-populations **B**-Multiple comparison One Way ANOVA of rs358231 minor allele frequency in all super-populations, except populations ACB, GWD, and CDX.

A

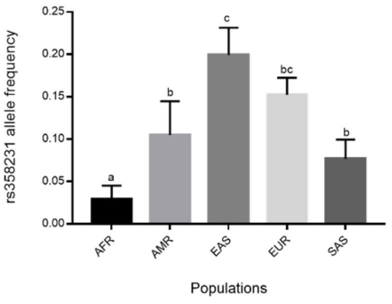

B

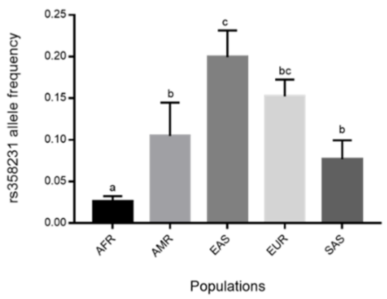

Supplementary Table 5: Accession numbers of sampled GBA3 sequences.

**1**		**Species**	**Order**	**Accession number GBA3**
**2**	HSA	*Homo sapiens*	Primate-Hominoidae	NM_020973.4 / NR_102355.1Ψ
**3**	NLE	*Nomascus leucogenys*	Primate-Hominoidae	XM_003258535.3
**4**	GGO	*Gorilla gorilla*	Primate-Hominoidae	XM_004038495.2
**5**	PPA	*Pan paniscus*	Primate-Hominoidae	XM_003826830.2
**6**	PAB	*Pongo abelii*	Primate-Hominoidae	NM_001131233.1
**7**	PTR	*Pan troglodytes*	Primate-Hominoidae	XM_517125.7
**8**	MMUL	*Macaca mulatta*	Primate- Haplorrhini-Cercopithecoidea	XM_001105060.3
**9**	MNE	*Macaca nemestrina*	Primate- Haplorrhini-Cercopithecoidea	XM_011751477.1
**10**	CAT	*Cercocebus atys*	Primate- Haplorrhini-Cercopithecoidea	XM_012045319.1
**11**	TGE	*Theropithecus gelada*	Primate- Haplorrhini-Cercopithecoidea	XM_025385498.1
**12**	CAN	*Colobus angolensis palliatus*	Primate- Haplorrhini-Cercopithecoidea	XM_011935661.1
**13**	PAN	*Papio anubis*	Primate- Haplorrhini-Cercopithecoidea	XM_021938915.1 partial sequence see supplementary material 2 for full CDS
**14**	MLE	*Mandrillus leucophaeus*	Primate- Haplorrhini-Cercopithecoidea	XM_011991304.1
**15**	CSA	*Chlorocebus sabaeus*	Primate- Haplorrhini-Cercopithecoidea	XM_008017751.1
**16**	RBI	*Rhinopithecus bieti*	Primate- Haplorrhini-Cercopithecoidea	XM_017866791.1
**17**	RRO	*Rhinopithecus roxellana*	Primate- Haplorrhini-Cercopithecoidea	XM_010377215.1
**18**	PTE	*Piliocolobus tephrosceles*	Primate-Haplorrhini-Cercopithecoidea	XM_023223045.2**?** coding status undetermined see supplementary material 2 for details
**19**	ANA	*Aotus nancymaae*	Primate- Haplorrhini-Platyrrhini	XM_012472455.2 poor annotation see supplementary material 2 for full CDS
**20**	SBO	*Saimiri boliviensis boliviensis*	Primate- Haplorrhini-Platyrrhini	XM_003927481.2
**21**	CCA	*Cebus capucinus imitator*	Primate- Haplorrhini-Platyrrhini	XM_017509816.1
**22**	CJA	*Callithrix jacchus*	Primate- Haplorrhini-Platyrrhini	XM_017970075.1 poor annotation see supplementary material 2 for full CDS
**23**	OGA	*Otolemur garnettii*	Primate- Strepsirrhini- Lorisiformes	XM_023515325.1
**24**	MMUR	*Microcebus murinus*	Primate- Strepsirrhini- Lemuriformes	XM_012737486.2
**25**	NGA	*Nannospalax galili*	Rodentia-Myomorpha	XM_029565547.1
**26**	MMU	*Mus musculus*	Rodentia-Myomorpha	No annotation predicted as pseudogene see supplementary material 3 for predicted CDS
**27**	RNO	*Rattus norvegicus*	Rodentia-Myomorpha	XM_006251045.2
**28**	MMA	*Marmota marmota marmota*	Rodentia-Sciuromorpha	XM_015482000.1
**29**	ITR	*Ictidomys tridecemlineatus*	Rodentia-Sciuromorpha	XM_005318922.2
**30**	DOR	*Dipodomys ordii*	Rodentia-Castorimorpha	XM_013009201.1
**31**	CPO	*Cavia porcellus*	Rodentia-Hystricomorpha	NM_001173119.1
**32**	HGL	*Heterocephalus glaber*	Rodentia-Hystricomorpha	XM_021260221.1 Ψ
**33**	FDA	*Fukomys damarensis*	Rodentia-Hystricomorpha	LOC104852682 Ψ
**34**	OCU	*Oryctolagus cuniculus*	Lagomorpha	XM_002709257.3
**35**	OPR	*Ochotona princeps*	Lagomorpha	XM_004579340.1
**36**	SSC	*Sus scrofa*	Cetartiodactyla- Suina	XM_021101070.1
**37**	CDR	*Camelus dromedarius*	Cetartiodactyla- Camelidae	XM_010991046.1
**38**	CFE	*Camelus ferus*	Cetartiodactyla- Camelidae	XM_006185590.2
**39**	BTA	*Bos taurus*	Cetartiodactyla- Ruminantia-Bovinae	NM_001191277.2
**40**		*Bos mutus*	Cetartiodactyla- Ruminantia-Bovinae	XM_005897074.2
**41**		*Bubalis bubalis*	Cetartiodactyla- Ruminantia-Bovinae	XM_006063273.2
**42**	OAR	*Ovis aries*	Cetartiodactyla- Ruminantia-Caprinae	XM_004009737.4
**43**	CHI	*Capra hircus*	Cetartiodactyla- Ruminantia-Caprinae	XM_005681471.3
**44**	OOR	*Orcinus orca*	Cetartiodactyla-Cetacea-Odontoceti	XM_004266259.1 Ψ
**45**	TTR	*Tursiops truncatus*	Cetartiodactyla-Cetacea-Odontoceti	LOC101326621 Ψ
**46**	DLE	*Delphinapterus leucas*	Cetartiodactyla-Cetacea-Odontoceti	LOC111171607 Ψ
**47**	LVE	*Lipotes vexillifer*	Cetartiodactyla-Cetacea-Odontoceti	LOC103080649 Ψ
**48**	PCA	*Physeter catodon*	Cetartiodactyla-Cetacea-Mysticeti	LOC102973494 Ψ
**49**	BAC	*Balaenoptera acutorostrata*	Cetartiodactyla-Cetacea-Mysticeti	LOC103004921 Ψ
**50**	BMY	*Balaena mysticetus*	Cetartiodactyla-Cetacea-Mysticeti	BL_ORD_ID|966 Ψ (genomic scaffold)
**51**	HAM	*Hippopotamus amphibius*	Cetartiodactyla- Hippopotamidae	PVJP01030293.1 (genomic scaffold) PVJP01003924.1 (genomic scaffold)
**52**	CSI	*Ceratotherium simum simum*	Perissodactyla-Rhinoceratidae	XM_004432315.2
**53**	ECA	*Equus caballus*	Perissodactyla-Equidea	XM_001497897.4
**54**	EPR	*Equus przewalskii*	Perissodactyla-Equidea	XM_008541503.1

**55**	ORO	*Odobenus rosmarus divergens*	Carnivora -Caniformia	XM_004402882.2 Ψ
**56**	NSC	*Neomonachus schauinslandi*	Carnivora -Caniformia	LOC110571335 Ψ
**57**	LWE	*Leptonychotes weddellii*	Carnivora -Caniformia	XM_006731631.1 Ψ
**58**	CUR	*Callorhinus ursinus*	Carnivora -Caniformia	XM_025887524.1 Ψ
**59**	EJU	*Eumetopias jubatus*	Carnivora -Caniformia	XM_028087323.1 Ψ
**60**	ZCA	*Zalophus californianus*	Carnivora -Caniformia	XM_027600122.1 Ψ
**61**	AGA	*Arctocephalus gazella*	Carnivora -Caniformia	UIRR01000237.1 Ψ (genomic scaffold)
**62**	MAN	*Mirounga angustirostris*	Carnivora -Caniformia	PITE01004996.1 Ψ (genomic scaffold)<br>PITE01008600.1 Ψ (genomic scaffold)
**63**	AFU	*Ailurus fulgens*	Carnivora -Caniformia	LNAC01000011.1 (genomic scaffold)
**64**	VVU	*Vulpes vulpes*	Carnivora -Caniformia	XM_025997289.1 Ψ
**65**	CLU	*Canis lupus familiaris*	Carnivora -Caniformia	XM_014112662.2
**66**	CLUD	*Canis lupus dingo*	Carnivora -Caniformia	XM_025437935.1
**67**	LPI	*Lycaon pictus*	Carnivora -Caniformia	CM007567.1 Ψ (genomic scaffold) female<br>CM007414.1 Ψ (genomic scaffold) male
**68**	VLA	*Vulpes lagopus*	Carnivora -Caniformia	PISU010009032.1 (genomic scaffold)<br>PISU010000078.1(genomic scaffold)
**69**	MFU	*Mustela putorius furo*	Carnivora -Caniformia	XM_004762687.2
**70**	ELU	*Enhydra lutris kenyoni*	Carnivora -Caniformia	XM_022506284.1
**71**	UMA	*Ursus maritimus*	Carnivora -Caniformia	XM_008685917.1
**72**	UAR	*Ursus arctos horribilis*	Carnivora -Caniformia	XM_026514522.1
**73**	AME	*Ailuropoda melanoleuca*	Carnivora -Caniformia	XM_019796531.1
**74**	UAM	*Ursos americanus*	Carnivora -Caniformia	LZNR01004097.1 (genomic scaffold)
**75**	AJU	*Acinonyx jubatus*	Carnivora -Feliformia	XM_015085052.2
**76**	PPAR	*Panthera pardus*	Carnivora -Feliformia	XM_019453597.1
**77**	PCON	*Puma concolor*	Carnivora -Feliformia	XM_025921973.1
**78**	PALT	*Panthera tigris altaica*	Carnivora -Feliformia	XM_015542724.1
**79**	FCA	*Felis catus*	Carnivora -Feliformia	XM_019829687.1
**80**	FNI	*Felis nigripes*	Carnivora -Feliformia	PISY01000073.1 (genomic scaffold)<br>PISY01044328.1 (genomic scaffold)
**81**	PON	*Panthera onca*	Carnivora -Feliformia	PISV01017856.1 (genomic scaffold)<br>PISV01004743.1 (genomic scaffold)<br>PISV01000042.1 (genomic scaffold)
**82**	PVA	*Pteropus vampyrus*	Chiroptera	XM_023534119.1 Ψ
**83**	PAL	*Pteropus alecto*	Chiroptera	XM_025051697.1 Ψ
**84**	DRO	*Desmodus rotundus*	Chiroptera	XM_024573544.1 Ψ
**85**	MDA	*Myotis davidii*	Chiroptera	XM_006771043.2
**86**	MLU	*Myotis lucifugus*	Chiroptera	XM_006093276.2
**87**	PDI	*Phyllostomus discolor*	Chiroptera	XM_028505603.1
**88**	EFU	*Eptesicus fuscus*	Chiroptera	XM_008140188.2
**89**	HAR	*Hipposideros armiger*	Chiroptera	XM_019642393.1
**90**	CAS	*Chrysochloris asiatica*	Afrotheria-Afrosoricida	XM_006866292.1
**91**	OAF	*Orycteropus afer afer*	Afrotheria-Tubulidentata	XM_007954826.1
**92**	LAF	*Loxodonta africana*	Afrotheria-Proboscidea	LOC100664259 Ψ
**93**	MJA	*Manis javanica*	Pholidota	XM_017647708.1
**94**	CCR	*Condylura cristata*	Insectivora-Talpidae	XM_012724085.1
**95**	SAR	*Sorex araneus*	Insectivora-Eulipotyphla	XM_004616552.1
**96**	EEU	*Erinaceus europaeus*	Insectivora- Erinaceomorpha	XM_007523878.2
**97**	DNO	*Dasypus novemcinctus*	Xenartha-Cingulata	XM_004477758.2
**98**	MDO	*Monodelphis domestica*	Marsupialia	XM_007496658.2
**99**	OAN	*Ornithorhynchus anatinus*	Monotremata	XM_007673226.1

Supplementary Table 6: Accession numbers of sampled NEU2 sequences.

1		Species	Order	Accession number NEU2
2	HSA	*Homo sapiens*	Primate-Hominoidae	NM_005383.2
3	NLE	*Nomascus leucogenys*	Primate-Hominoidae	XM_003274757.1
4	GGO	*Gorilla gorilla*	Primate-Hominoidae	XM_004033375.1
5	PPA	*Pan paniscus*	Primate-Hominoidae	XM_003813133.1
6	PAB	*Pongo abelii*	Primate-Hominoidae	XM_003776111.2
7	PTR	*Pan troglodytes*	Primate-Hominoidae	XM_003309526.3
8	MMUL	*Macaca mulatta*	Primate-Haplorrhini-Cercopithecoidea	XM_001114628.2
9	MNE	*Macaca nemestrina*	Primate-Haplorrhini-Cercopithecoidea	XM_011728084.1
10	CAT	*Cercocebus atys*	Primate Haplorrhini-Cercopithecoidea	XM_012072697.1
11	TGE	*Theropithecus gelada*	Primate-Haplorrhini-Cercopithecoidea	XM_025405520.1
12	CAN	*Colobus angolensis palliatus*	Primate-Haplorrhini-Cercopithecoidea	XM_011952023.1
13	PAN	*Papio anubis*	Primate-Haplorrhini-Cercopithecoidea	XM_003908102.2
14	MLE	*Mandrillus leucophaeus*	Primate-Haplorrhini-Cercopithecoidea	XM_011980247.1
15	CSA	*Chlorocebus sabaeus*	Primate-Haplorrhini-Cercopithecoidea	XM_007966683.1
16	RBI	*Rhinopithecus bieti*	Primate-Haplorrhini-Cercopithecoidea	XM_017859367.1
17	RRO	*Rhinopithecus roxellana*	Primate-Haplorrhini-Cercopithecoidea	XM_010353137.1
18	PTE	*Piliocolobus tephrosceles*	Primate-Haplorrhini-Cercopithecoidea	XM_023188580.1
19	ANA	*Aotus nancymaae*	Primate-Haplorrhini-Platyrrhini	XM_021676892.1
20	SBO	*Saimiri boliviensis boliviensis*	Primate-Haplorrhini-Platyrrhini	XM_003936729.1
21	CCA	*Cebus capucinus imitator*	Primate-Haplorrhini-Platyrrhini	XM_017537831.1
22	CJA	*Callithrix jacchus*	Primate-Haplorrhini-Platyrrhini	XM_002749910.1
23	OGA	*Otolemur garnettii*	Primate-Strepsirrhini- Lorisiformes	XM_003785056.3
24	MMUR	*Microcebus murinus*	Primate-Strepsirrhini- Lemuriformes	XM_012791567.1
25	NGA	*Nannospalax galili*	Rodentia-Myomorpha	XM_029565924.1
26	MMU	*Mus musculus*	Rodentia-Myomorpha	NM_001160163.1
27	RNO	*Rattus norvegicus*	Rodentia-Myomorpha	NM_017130.2
28	MMA	*Marmota marmota marmota*	Rodentia-Sciuromorpha	XM_015481578.1
29	ITR	*Ictidomys tridecemlineatus*	Rodentia-Sciuromorpha	XM_005326486.1
30	DOR	*Dipodomys ordii*	Rodentia-Castorimorpha	XM_013022524.1
31	CPO	*Cavia porcellus*	Rodentia-Hystricomorpha	XM_003474409.2
32	HGL	*Heterocephalus glaber*	Rodentia-Hystricomorpha	XM_021247102.1
33	FDA	*Fukomys damarensis*	Rodentia-Hystricomorpha	XM_010631791.1 Ψ
34	OCU	*Oryctolagus cuniculus*	Lagomorpha	XM_017337538.1
35	OPR	*Ochotona princeps*	Lagomorpha	XM_004577193.1
36	SSC	*Sus scrofa*	Cetartiodactyla-Suina	XM_013984592.2
37	CDR	*Camelus dromedarius*	Cetartiodactyla-Camelidae	XM_010999029.1
38	CFE	*Camelus ferus*	Cetartiodactyla-Camelidae	XM_014564719.1
39	BTA	*Bos taurus*	Cetartiodactyla-Ruminantia-Bovinae	XM_024989842.1
40	BMU	*Bos mutus*	Cetartiodactyla-Ruminantia-Bovinae	XM_005895025.1
41	BBU	*Bubalis bubalis*	Cetartiodactyla-Ruminantia-Bovinae	XM_006042655.2
42	OAR	*Ovis aries*	Cetartiodactyla-Ruminantia-Caprinae	XM_027964281.1
43	CHI	*Capra hircus*	Cetartiodactyla-Ruminantia-Caprinae	XM_005678741.3
44	OOR	*Orcinus orca*	Cetartiodactyla-Cetacea-Odontoceti	XM_004262913.1 Ψ
45	TTR	*Tursiops truncatus*	Cetartiodactyla-Cetacea-Odontoceti	XM_019923440.1 Ψ
46	DLE	*Delphinapterus leucas*	Cetartiodactyla-Cetacea-Odontoceti	XM_022568338.1 Ψ
47	LVE	*Lipotes vexillifer*	Cetartiodactyla-Cetacea-Odontoceti	XM_007445735.1 Ψ
48	PCA	*Physeter catodon*	Cetartiodactyla-Cetacea-Mysticeti	XM_024124749.1 Ψ
49	BAC	*Balaenoptera acutorostrata*	Cetartiodactyla-Cetacea-Mysticeti	XM_007184894.1 Ψ
50	BMY	*Balaena mysticetus*	Cetartiodactyla-Cetacea-Mysticeti	BL_ORD_ID|1272 Ψ (genomic scaffold)
51	HAM	*Hippopotamus amphibius*	Cetartiodactyla- Hippopotamidae	NKPW01001218.1 (genomic scaffold)
52	CSI	*Ceratotherium simum simum*	Perissodactyla-Rhinoceratidae	XM_004427693.2
53	ECA	*Equus caballus*	Perissodactyla-Equidea	XM_023642592.1
54	EPR	*Equus przewalskii*	Perissodactyla-Equidea	XM_008527835.1
55	ORO	*Odobenus rosmarus divergens*	Carnivora -Caniformia	XM_004396087.1
56	NSC	*Neomonachus schauinslandi*	Carnivora -Caniformia	XM_021693827.1
57	LWE	*Leptonychotes weddellii*	Carnivora -Caniformia	XM_006751012.1
58	CUR	*Callorhinus ursinus*	Carnivora -Caniformia	XM_025886671.1
59	EJU	*Eumetopias jubatus*	Carnivora -Caniformia	XM_028105060.1
60	ZCA	*Zalophus californianus*	Carnivora -Caniformia	XM_027591235.1

**61**	AGA	*Arctocephalus gazella*	Carnivora -Caniformia	UIRR01000070.1 (genomic scaffold)
**62**	MAN	*Mirounga angustirostris*	Carnivora -Caniformia	PITE01011843.1 (genomic scaffold)
**63**	AFU	*Ailurus fulgens*	Carnivora -Caniformia	LNAC01000115.1 (genomic scaffold)
**64**	VVU	*Vulpes vulpes*	Carnivora -Caniformia	XM_026006232.1
**65**	CLU	*Canis lupus familiaris*	Carnivora -Caniformia	XM_005635862.3
**66**	CLUD	*Canis lupus dingo*	Carnivora -Caniformia	XM_025463474.1
**67**	LPI	*Lycaon pictus*	Carnivora -Caniformia	CM007437.1 (genomic scaffold)
**68**	VLA	*Vulpes lagopus*	Carnivora -Caniformia	PISU010007680.1 (genomic scaffold)
**69**	MFU	*Mustela putorius furo*	Carnivora -Caniformia	XM_004773496.2
**70**	ELU	*Enhydra lutris kenyoni*	Carnivora -Caniformia	XM_022498075.1
**71**	UMA	*Ursus maritimus*	Carnivora -Caniformia	XM_008690548.1
**72**	UAR	*Ursus arctos horribilis*	Carnivora -Caniformia	XM_026491680.1
**73**	AME	*Ailuropoda melanoleuca*	Carnivora -Caniformia	XM_019798290.1
**74**	UAM	*Ursus americanus*	Carnivora -Caniformia	LZNR01005881.1 (genomic scaffold)
**75**	AJU	*Acinonyx jubatus*	Carnivora -Feliformia	XM_027047457.1
**76**	PPAR	*Panthera pardus*	Carnivora -Feliformia	XM_019445537.1
**77**	PCON	*Puma concolor*	Carnivora -Feliformia	XM_025912617.1
**78**	PALT	*Panthera tigris altaica*	Carnivora -Feliformia	XM_007082978.2
**79**	FCA	*Felis catus*	Carnivora -Feliformia	XM_003991289.5
**80**	FNI	*Felis nigripes*	Carnivora -Feliformia	PISY01109988.1 (genomic scaffold)
**81**	PON	*Panthera onca*	Carnivora -Feliformia	PISV01015656.1 (genomic scaffold)
**82**	PVA	*Pteropus vampyrus*	Chiroptera	XM_011380874.1
**83**	PAL	*Pteropus alecto*	Chiroptera	XM_006906623.1
**84**	DRO	*Desmodus rotundus*	Chiroptera	XM_024564056.1
**85**	MDA	*Myotis davidii*	Chiroptera	XM_015561998.1
**86**	MLU	*Myotis lucifugus*	Chiroptera	XM_023759553.1
**87**	PDI	*Phyllostomus discolor*	Chiroptera	XM_028511010.1
**88**	EFU	*Eptesicus fuscus*	Chiroptera	XM_028140585.1
**89**	HAR	*Hipposideros armiger*	Chiroptera	XM_019628264.1
**90**	CAS	*Chrysochloris asiatica*	Afrotheria-Afrosoricida	XM_006866760.1
**91**	OAF	*Orycteropus afer afer*	Afrotheria-Tubulidentata	XM_007941754.1
**92**	LAF	*Loxodonta africana*	Afrotheria-Proboscidea	XM_003417959.3
**93**	MJA	*Manis javanica*	Pholidota	XM_017643222.1
**94**	CCR	*Condylura cristata*	Insectivora-Talpidae	XM_004674189.1
**95**	SAR	*Sorex araneus*	Insectivora-Eulipotyphla	XM_004610876.1
**96**	EEU	*Erinaceus europaeus*	Insectivora- Erinaceomorpha	XM_007520388.1
**97**	DNO	*Dasypus novemcinctus*	Xenartha-Cingulata	Poor coverage
**98**	MDO	*Monodelphis domestica*	Marsupialia	XM_016429321.1
**99**	OAN	*Ornithorhynchus anatinus*	Monotremata	XM_007664152.2

Supplementary Table 7: Branch models tested in CODEML

Model	Description	Assumption
**A. one ratio**	$\omega_0 = \omega_{A1} = \omega_{BranchA1} = \omega_{A2} = \omega_{A3} = \omega_{B1} = \omega_{BranchB1} = \omega_{B2} = \omega_{C1} = \omega_{D1}$	Null model, no variation in selective pressures in all species analyzed
**B. two rates**	$\omega_0 = \omega_{A2} = \omega_{A3} = \omega_{B1} = \omega_{BranchB1} = \omega_{B2} = \omega_{C1} = \omega_{D1}$, $\boldsymbol{\omega_{A1} = \omega_{BranchA1}}$	Pinnipedia Clade A1 and Pinniped Branch A1 shift from ω0
**C. two rates**	$\omega_0 = \omega_{A1} = \omega_{BranchA1} = \omega_{A3} = \omega_{B1} = \omega_{BranchB1} = \omega_{B2} = \omega_{C1} = \omega_{D1}$, $\boldsymbol{\omega_{A2}}$	Canidae Clade A2 shifts from ω0
**D. two rates**	$\omega_0 = \omega_{A1} = \omega_{BranchA1} = \omega_{A2} = \omega_{B1} = \omega_{BranchB1} = \omega_{B2} = \omega_{C1} = \omega_{D1}$, $\boldsymbol{\omega_{A3}}$	Feliformia Clade A3 shifts from ω0
**E. two rates**	$\omega_0 = \omega_{A1} = \omega_{BranchA1} = \omega_{A2} = \omega_{A3} = \omega_{B2} = \omega_{C1} = \omega_{D1}$, $\boldsymbol{\omega_{B1} = \omega_{BranchB1}}$	Cetacea Clade B1 and Cetacea Branch B1 shift from ω0
**F. two rates**	$\omega_0 = \omega_{A1} = \omega_{BranchA1} = \omega_{A2} = \omega_{A3} = \omega_{B1} = \omega_{BranchB1} = \omega_{C1} = \omega_{D1}$, $\boldsymbol{\omega_{B2}}$	Ruminantia Clade B2 shifts from ω0
**G. two rates**	$\omega_0 = \omega_{A1} = \omega_{A2} = \omega_{A3} = \omega_{B1} = \omega_{BranchB1} = \omega_{B2} = \omega_{C1} = \omega_{D1}$, $\boldsymbol{\omega_{BranchA1}}$	Pinnipedia Branch A1 shifts from ω0
**H. two rates**	$\omega_0 = \omega_{A1} = \omega_{BranchA1} = \omega_{A2} = \omega_{A3} = \omega_{B1} = \omega_{B2} = \omega_{C1} = \omega_{D1}$, $\boldsymbol{\omega_{BranchB1}}$	Cetacea Branch B1 shifts from ω0
**I. two rates**	$\omega_0 = \omega_{A1} = \omega_{BranchA1} = \omega_{A2} = \omega_{A3} = \omega_{B1} = \omega_{B2} = \omega_{BranchB1} = \omega_{D1}$, $\boldsymbol{\omega_{C1}}$	Chiropetra Clade C1 shifts from ω0
**J. two rates**	$\omega_0 = \omega_{A1} = \omega_{BranchA1} = \omega_{A2} = \omega_{A3} = \omega_{B1} = \omega_{B2} = \omega_{BranchB1} = \omega_{C1}$, $\boldsymbol{\omega_{D1}}$	Rodentia Clade D1 shifts from ω0
**K. two rates**	$\omega_0 = \omega_{A2} = \omega_{A3} = \omega_{B2} = \omega_{C1} = \omega_{D1}$, $\boldsymbol{\omega_{A1} = \omega_{BranchA1} = \omega_{B1} = \omega_{BranchB1}}$	Pinnipedia Clade A1, Pinniped Branch A1, Cetacea Clade B1 and Cetacea Branch B1 equally shift from ω0
**L. three rates**	$\omega_0 = \omega_{A2} = \omega_{A3} = \omega_{B2} = \omega_{C1} = \omega_{D1}$, $\boldsymbol{\omega_{A1} = \omega_{BranchA1}}$, $\omega_{B1} = \omega_{BranchB1}$	Pinnipedia Clade A1 and Pinnipedia Branch A1 shift from Cetacea Clade B1 and Cetacea Branch B1, and both shift from ω0
**M. four rates**	$\omega_0 = \omega_{A2} = \omega_{A3} = \omega_{B2} = \omega_{C1} = \omega_{D1}$, $\boldsymbol{\omega_{A1}}$, ω_{B1}, $\boldsymbol{\omega_{BranchB1} = \omega_{BranchA1}}$	Pinnipedia Clade A1 shifts from ω0, Cetacea Clade B1 shifts from ω0, Cetacea Branch B1 and Pinnipedia Branch A1 equally shifts from ω0
**N. five rates**	$\omega_0 = \omega_{A2} = \omega_{A3} = \omega_{B2} = \omega_{C1} = \omega_{D1}$, $\boldsymbol{\omega_{A1}}$, $\omega_{BranchA1}$, $\boldsymbol{\omega_{B1}}$, $\omega_{BranchB1}$	Pinnipedia Clade A1 shifts from ω0, Pinnipedia Branch A1 shifts from ω0, Cetacea Clade B1 shifts from ω0, Cetacea Branch B1 shifts from ω0, all shift independently

Supplementary Table 8: Specification of the Log Likelihood Values (lnL), w parameters and number of parameters computed for each tested branch model in CODEML.

Model	ω_0	ω_{A1}	$\omega_{BranchA1}$	ω_{A2}	ω_{A3}	ω_{B1}	$\omega_{BranchB1}$	ω_{B2}	ω_{C1}	ω_{D1}	np	lnL
A. one ratio: $\omega_0 = \omega_{A1} = \omega_{BranchA1} = \omega_{A2} = \omega_{A3} = \omega_{B1} = \omega_{BranchB1} = \omega_{B2} = \omega_{C1} = \omega_{D1}$	0.297	$=\omega_0$	$=\omega_0$	$=\omega_0$	$=\omega_0$	$=\omega_0$	$=\omega_0$	$=\omega_0$	$=\omega_0$	$=\omega_0$	192	-19159.91
B. two rates: $\omega_0 = \omega_{A2} = \omega_{A3} = \omega_{B1} = \omega_{BranchB1} = \omega_{B2} = \omega_{C1} = \omega_{D1}, \omega_{A1} = \omega_{BranchA1}$	0.288	1.058	1.058	$=\omega_0$	$=\omega_0$	$=\omega_0$	$=\omega_0$	$=\omega_0$	$=\omega_0$	$=\omega_0$	193	-19144.71
C. two rates: $\omega_0 = \omega_{A1} = \omega_{BranchA1} = \omega_{A3} = \omega_{B1} = \omega_{BranchB1} = \omega_{B2} = \omega_{C1} = \omega_{D1}, \omega_{A2}$	0.296	$=\omega_0$	$=\omega_0$	0.386	$=\omega_0$	$=\omega_0$	$=\omega_0$	$=\omega_0$	$=\omega_0$	$=\omega_0$	193	-19159.57
D. two rates: $\omega_0 = \omega_{A1} = \omega_{BranchA1} = \omega_{A2} = \omega_{B1} = \omega_{BranchB1} = \omega_{B2} = \omega_{C1} = \omega_{D1}, \omega_{A3}$	0.298	$=\omega_0$	$=\omega_0$	$=\omega_0$	0.252	$=\omega_0$	$=\omega_0$	$=\omega_0$	$=\omega_0$	$=\omega_0$	193	-19159.73
E. two rates: $\omega_0 = \omega_{A1} = \omega_{BranchA1} = \omega_{A2} = \omega_{A3} = \omega_{B2} = \omega_{C1} = \omega_{D1}, \omega_{B1} = \omega_{BranchB1}$	0.284	$=\omega_0$	$=\omega_0$	$=\omega_0$	$=\omega_0$	0.816	0.816	$=\omega_0$	$=\omega_0$	$=\omega_0$	193	-19142.41
F. two rates: $\omega_0 = \omega_{A1} = \omega_{BranchA1} = \omega_{A2} = \omega_{A3} = \omega_{B1} = \omega_{BranchB1} = \omega_{C1} = \omega_{D1}, \omega_{B2}$	0.298	$=\omega_0$	$=\omega_0$	$=\omega_0$	$=\omega_0$	$=\omega_0$	$=\omega_0$	0.231	$=\omega_0$	$=\omega_0$	193	-19159.42
G. two rates: $\omega_0 = \omega_{A1} = \omega_{A2} = \omega_{A3} = \omega_{B1} = \omega_{BranchB1} = \omega_{B2} = \omega_{C1} = \omega_{D1}, \omega_{BranchA1}$	0.296	$=\omega_0$	$=\omega_0$	1.797	$=\omega_0$	$=\omega_0$	$=\omega_0$	$=\omega_0$	$=\omega_0$	$=\omega_0$	193	-19158.14
H. two rates: $\omega_0 = \omega_{A1} = \omega_{BranchA1} = \omega_{A2} = \omega_{A3} = \omega_{B1} = \omega_{B2} = \omega_{C1} = \omega_{D1}, \omega_{BranchB1}$	0.297	$=\omega_0$	$=\omega_0$	$=\omega_0$	$=\omega_0$	$=\omega_0$	0.326	$=\omega_0$	$=\omega_0$	$=\omega_0$	193	-19159.88
I. two rates: $\omega_0 = \omega_{A1} = \omega_{BranchA1} = \omega_{A2} = \omega_{A3} = \omega_{B1} = \omega_{B2} = \omega_{BranchB1} = \omega_{D1}, \omega_{C1}$	0.294	$=\omega_0$	$=\omega_0$	$=\omega_0$	$=\omega_0$	$=\omega_0$	$=\omega_0$	$=\omega_0$	0.317	$=\omega_0$	193	-19159.67
J. two rates: $\omega_0 = \omega_{A1} = \omega_{BranchA1} = \omega_{A2} = \omega_{A3} = \omega_{B1} = \omega_{B2} = \omega_{BranchB1} = \omega_{C1}, \omega_{D1}$	0.290	$=\omega_0$	$=\omega_0$	$=\omega_0$	$=\omega_0$	$=\omega_0$	$=\omega_0$	$=\omega_0$	$=\omega_0$	0.328	193	-19158.99
K. two rates: $\omega_0 = \omega_{A2} = \omega_{A3} = \omega_{B2}, \omega_{A1} = \omega_{BranchA1} = \omega_{B1} = \omega_{BranchB1}$	0.274	0.896	0.896	$=\omega_0$	$=\omega_0$	0.896	0.896	$=\omega_0$	$=\omega_0$	$=\omega_0$	193	-19126.38
L. three rates: $\omega_0 = \omega_{A2} = \omega_{A3} = \omega_{B2} = \omega_{C1} = \omega_{D1}, \omega_{A1} = \omega_{BranchA1}, \omega_{B1} = \omega_{BranchB1}$	0.274	1.058	1.058	$=\omega_0$	$=\omega_0$	0.816	0.816	$=\omega_0$	$=\omega_0$	$=\omega_0$	194	-19126.03
M. four rates: $\omega_0 = \omega_{A2} = \omega_{A3} = \omega_{B2} = \omega_{C1} = \omega_{D1}, \omega_{A1} = \omega_{B1}, \omega_{BranchB1}, \omega_{BranchA1}$	0.274	1.033	1.726	$=\omega_0$	$=\omega_0$	1.033	0.322	$=\omega_0$	$=\omega_0$	$=\omega_0$	195	-19122.60
N. five rates: $\omega_0 = \omega_{A2} = \omega_{A3} = \omega_{B2} = \omega_{C1} = \omega_{D1}, \omega_{A1}, \omega_{BranchA1}, \omega_{B1}, \omega_{BranchB1}$	0.274	1.024	1.726	$=\omega_0$	$=\omega_0$	1.038	0.322	$=\omega_0$	$=\omega_0$	$=\omega_0$	196	-19122.60
