# Supplementary material for: GBA3: a polymorphic pseudogene in humans that experienced repeated gene loss during mammalian evolution
Source: Sci Rep. 2020 Jul 14;10:11565. doi: 10.1038/s41598-020-68106-y (PMC7360587; doi:10.1038/s41598-020-68106-y)
Supplement: Supplementary file 2 — Supplementary Material [file 41598_2020_68106_MOESM2_ESM.pdf]

SUPPLEMENTARY MATERIAL

GBA3: a polymorphic pseudogene in humans that experienced repeated gene loss during mammalian evolution

Lopes-Marques, Monica[1,2,3ǂ]; Serrano, Catarina [1,2,3]; Cardoso, Ana R. [1,2,3]; Salazar, Renato[1,2]; Seixas, Susana[1,2], Amorim, António[1,2,3]; Azevedo, Luisa[1,2,3*] Prata, Maria J[1,2,3*]

[1]i3S- Instituto de Investigação e Inovação em Saúde, Universidade do Porto, Population Genetics and Evolution Group, Rua Alfredo Allen 208, 4200-135 Porto, Portugal

[2]IPATIMUP-Institute of Molecular Pathology and Immunology, University of Porto, Rua Júlio Amaral de Carvalho 45, 4200-135 Porto, Portugal

[3]Department of Biology, Faculty of Sciences, University of Porto, Rua do Campo Alegre, s/n, 4169-007 Porto, Portugal

SUPPLEMENTARY MATERIAL- 1

Supplementary Material 1A- 1KGP genotype distribution

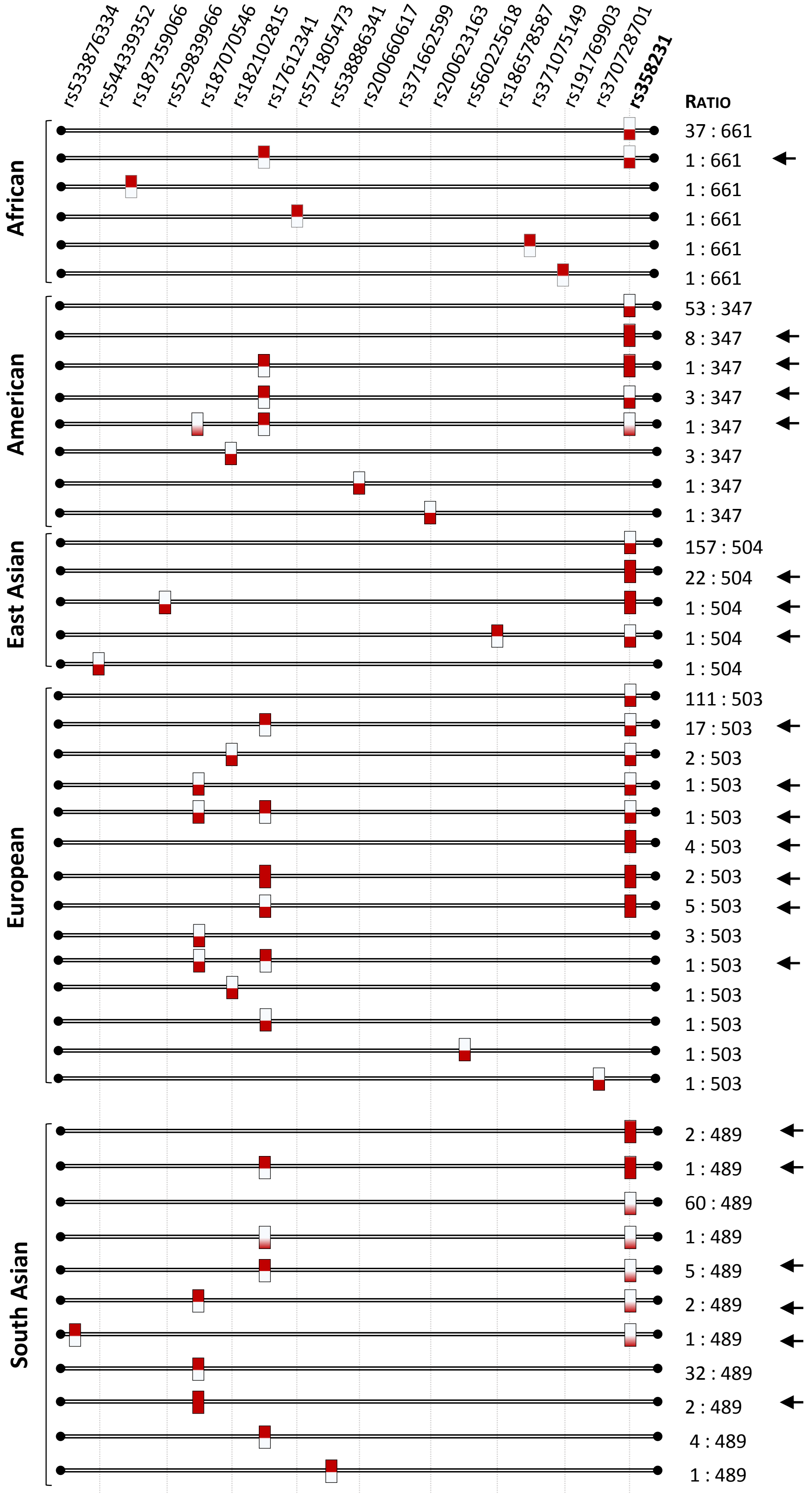

FigureS1: Schematic representation of the 1KGP genotypes including the predicted damaging variants. On top variant ID, each line represents a chromosome, thus double lines represent both chromosomes variant alleles are represented by boxes, homozygous for the damaging allele, heterozygous for the damaging allele, no box indicates homozygous for the ancestral state. Ratio on the right indicates the number of individuals with the corresponding genotype in the total of the corresponding population analyzed. Populations are indicated on the right. Black arrows highlight genotypes carrying two damaging alleles in distinct chromosomes.

Supplementary Material 1B- gnomAD SVs V2.1.1 Annotation

Loss of function mutations present in *GBA3* and corresponding allele frequencies. Data extracted from gnomAD Browser (https://gnomad.broadinstitute.org/) last accessed 21/11/2019

	CHROMOSOME	RSID	REF	ALT	CONSEQUENCE	ANNOTATION	ALLELE COUNT	ALLELE NUMBER	ALLELE FREQUENCY	HOMOZYGOTE COUNT	HEMIZYGOTE COUNT
1	4	rs747981571	G	GC	p.Ala5CysfsTer82	frameshift_variant	1	245252	4,07744E-06	0	0
2	4	rs576810567	C	T	p.Gln17Ter	stop_gained	5	241874	2,06719E-05	0	0
3	4	rs1490196128	G	A	p.Trp22Ter	stop_gained	1	248038	4,03164E-06	0	0
4	4	rs1445474143	GC	G	p.Pro29LeufsTer23	frameshift_variant	1	248442	4,02508E-06	0	0
5	4	rs767570401	G	A	p.Trp32Ter	stop_gained	1	248604	4,02246E-06	0	0
6	4	rs760658989	CTT	C	p.Leu69ArgfsTer17	frameshift_variant	1	248942	4,017E-06	0	0
7	4	rs746343429	T	A	p.Leu84Ter	stop_gained	1	248436	4,02518E-06	0	0
8	4	rs747784699	AG	A	p.Gly90ValfsTer19	frameshift_variant	11	247826	4,4386E-05	0	0
9	4	rs1293001937	C	A	p.Tyr100Ter	stop_gained	1	246424	4,05805E-06	0	0
10	4		A	AGATC	p.Ile104AspfsTer4	frameshift_variant	1	247102	4,04691E-06	0	0
11	4	rs1263087168	TC	T	p.Ile115LeufsTer2	frameshift_variant	1	249020	4,01574E-06	0	0
12	4	rs751675029	A	AC	p.Leu118ProfsTer5	frameshift_variant	5	249104	2,00719E-05	0	0
13	4	rs201348337	GA	G	p.Glu136GlyfsTer28	frameshift_variant	18	249248	7,22172E-05	0	0
14	4	rs1156870427	CT	C	p.Phe142LeufsTer22	frameshift_variant	1	249258	4,01191E-06	0	0
15	4	rs1254685798	C	T	p.Gln159Ter	stop_gained	1	249220	4,01252E-06	0	0
16	4	rs971016595	G	A	p.Trp205Ter	stop_gained	1	249078	4,01481E-06	0	0
17	4	rs756397054	CA	C	p.Ser207AlafsTer69	frameshift_variant	12	280518	4,2778E-05	0	0
18	4	rs971793842	AT	A	p.Arg213GlufsTer63	frameshift_variant	2	249116	8,02839E-06	0	0
19	4	rs201515557	C	T	p.Arg213Ter	stop_gained	14	280344	4,99386E-05	1	0
20	4	rs777757169	AGCAG	A	p.Gln216LysfsTer59	frameshift_variant	1	249096	4,01452E-06	0	0
21	4	rs1285494537	C	T	p.Gln216Ter	stop_gained	1	249088	4,01465E-06	0	0
22	4	rs760316620	A	T	p.Lys217Ter	stop_gained	1	249110	4,01429E-06	0	0
23	4	rs777616477	C	A	p.Ser236Ter	stop_gained	1	249006	4,01597E-06	0	0
24	4	rs745975584	C	T	p.Gln240Ter	stop_gained	1	249028	4,01561E-06	0	0
25	4	rs1424607429	GC	G	p.Ile247SerfsTer29	frameshift_variant	1	249042	4,01539E-06	0	0
26	4	rs762931123	G	T	p.Glu266Ter	stop_gained	3	249036	1,20465E-05	0	0
27	4	rs751403797	CA	C	p.Lys278SerfsTer19	frameshift_variant	2	248696	8,04195E-06	0	0
28	4	rs1002537137	AAGAAG	A	p.Lys294AsnfsTer7	frameshift_variant	2	246378	8,11761E-06	0	0
29	4	rs373689817	G	T	p.Glu293Ter	stop_gained	1	246260	4,06075E-06	0	0
30	4	rs1401056840	A	T	p.Lys295Ter	stop_gained	1	31398	3,18492E-05	0	0
31	4		A	AG	p.Thr300HisfsTer3	frameshift_variant	1	245190	4,07847E-06	0	0
32	4	rs776182136	A	AT	p.Ala305CysfsTer29	frameshift_variant	15	274866	5,4572E-05	0	0
33	4	rs1357871546	G	A	p.Trp345Ter	stop_gained	2	244460	8,1813E-06	0	0
34	4	rs371662599	C	G	p.Tyr347Ter	stop_gained	3	244054	1,22924E-05	0	0
35	4	rs771680026	TG	T	p.Gly352GlufsTer6	frameshift_variant	2	272028	7,35218E-06	0	0
36	4	rs1249105062	G	A	p.Trp351Ter	stop_gained	1	239826	4,16969E-06	0	0
37	4	rs747275559	T	A	p.Cys354Ter	stop_gained	2	233162	8,57773E-06	0	0
38	4	rs775637297	C	T	p.Gln405Ter	stop_gained	4	244816	1,63388E-05	0	0
39	4	rs778733949	G	A	p.Trp417Ter	stop_gained	3	248206	1,20867E-05	0	0
40	4	rs745681466	C	G	p.Tyr429Ter	stop_gained	1	248392	4,02589E-06	0	0
41	4	rs1233129522	CG	C	p.Phe433LeufsTer42	frameshift_variant	6	248484	2,41464E-05	0	0
42	4	rs751059825	C	T	p.Arg447Ter	stop_gained	1	248512	4,02395E-06	0	0
43	4	rs538670192	C	T	p.Arg461Ter	stop_gained	2	248168	8,05906E-06	0	0

Supplementary Material 1B- gnomAD SVs V2.1.1 Annotation

Loss of function mutations present in *GBA* and corresponding allele frequencies. Data extracted from gnomAD Browser (https://gnomad.broadinstitute.org/) last accessed 21/11/2019

	CHROMOSOME	RSID	REF	ALT	CONSEQUENCE	ANNOTATION	ALLELE COUNT	ALLELE NUMBER	ALLELE FREQUENCY	HOMOZYGOTE COUNT	HEMIZYGOTE COUNT
**1**	1	rs750282937	ACTT G	A	p.Lys464SerfsTer24	frameshift_variant	1	250050	3,9992E-06	0	0
**2**	1	rs1484043383	C	T	p.Trp432Ter	stop_gained	1	251446	3,977E-06	0	0
**3**	1	rs754743440	C	T	p.Trp417Ter	stop_gained	1	251478	3,97649E-06	0	0
**4**	1	rs121908309	G	A	p.Arg398Ter	stop_gained	1	251386	3,97795E-06	0	0
**5**	1	rs781356917	C	CT	p.Phe386ValfsTer50	frameshift_variant	2	251440	7,95418E-06	0	0
**6**	1	rs1266525981	AG	A	p.Pro305LeufsTer31	frameshift_variant	1	251184	3,98115E-06	0	0
**7**	1	rs1201298815	GA	G	p.Ser212HisfsTer19	frameshift_variant	1	31388	3,18593E-05	0	0
**8**	1	rs1338037944	G	A	p.Gln205Ter	stop_gained	2	215522	9,2798E-06	0	0
**9**	1	rs1009850780	G	A	p.Arg202Ter	stop_gained	2	226974	8,81158E-06	0	0
**10**	1	rs1311482099	CAG	C	p.Leu199AspfsTer62	frameshift_variant	2	189032	1,05802E-05	0	0
**11**	1	rs779619231	CT	C	p.Glu111AsnfsTer7	frameshift_variant	2	251474	7,95311E-06	0	0
**12**	1	rs1050455670	GC	G	p.Ile95SerfsTer12	frameshift_variant	1	251290	3,97947E-06	0	0
**13**	1	rs1141810	ACT	A	p.Ser81TyrfsTer17	frameshift_variant	1	251334	3,97877E-06	0	0
**14**	1	rs769900428	CA	C	p.Gly74ValfsTer17	frameshift_variant	1	251342	3,97864E-06	0	0
**15**	1	rs750712570	C	CG	p.Thr69AspfsTer12	frameshift_variant	1	251298	3,97934E-06	0	0

Supplementary Material 1C- Average Tajima D and Diversity of the genomic *locus* containing *GBA3* using POPHUMAN browser

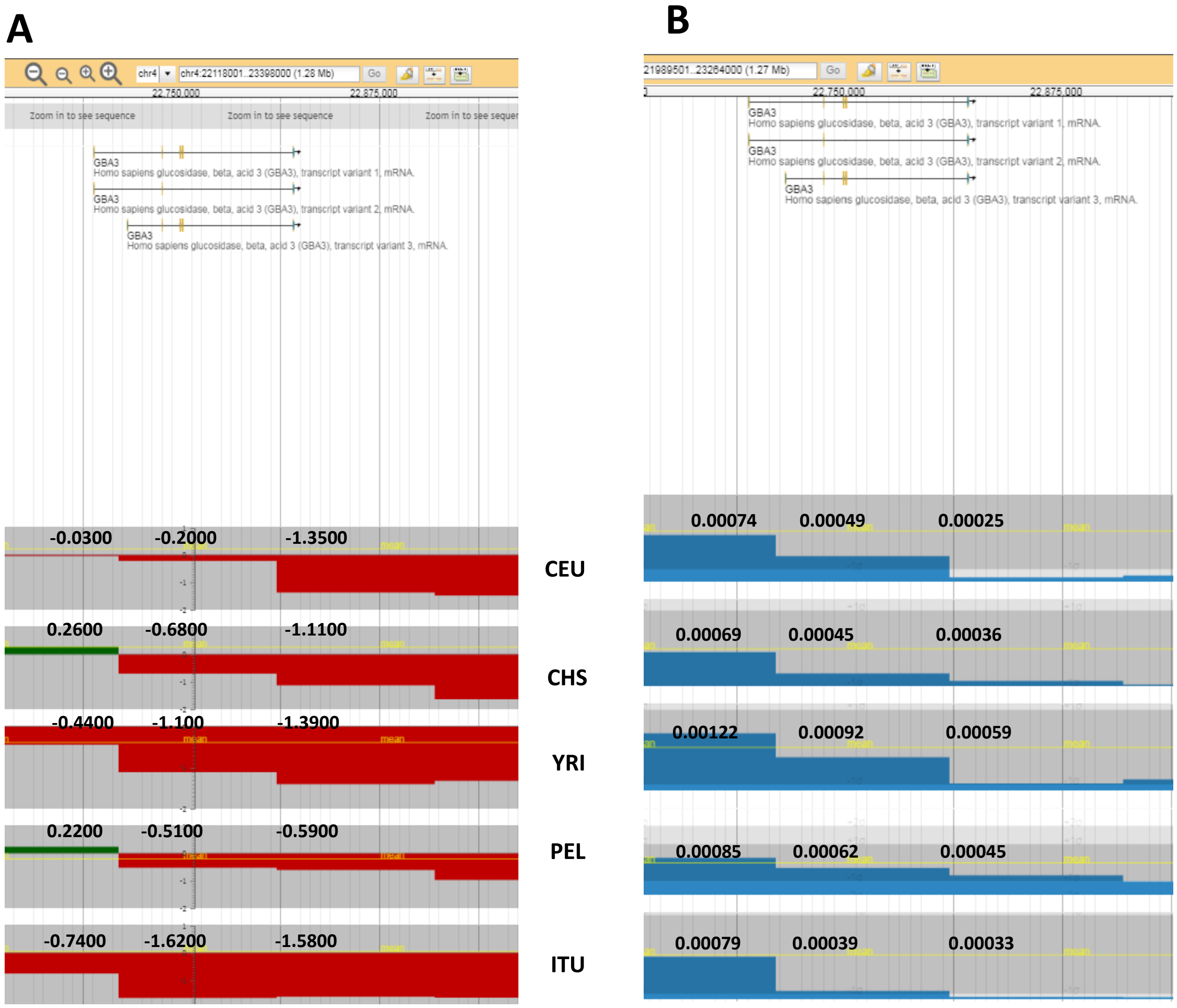

Figure S2: **A**- 100k sliding window Tajima D values for the 3 genomic tracks (22610195-22710195, 22710195-22810195 and 22810195-22910195) in the *GBA3* genomic region, yellow line represents mean value, green bares indicate values higher than mean, red bar indicate values below mean, grey bar indicates expected standard deviation from the mean, values for each 100k are indicated accordingly. **B** Nucleotide diversity for the 3 genomic tracks (22610195-22710195, 22710195-22810195 and 22810195-22910195) encompassing *GBA3*. Given that no population deviated from average, here we present one example per Mega population, CEU-European; East Asian-CHS, African-YRI; American- PEL; South Asian- ITU

SUPPLEMENTARY MATERIAL- 2

Supplementary Material 2 - GBA3 annotation in primates

In the case of primates namely *Aotus nancymaae* and *Papio anubis* while NCBI GBA3 annotation for these species is not tagged as LQ alignment of the collected GBA3 sequences (XM_012472455.2 and XM_021938915.1 respectively) revealed that NCBI sequences were 3´ partial missing the last exon of GBA3 in *A. nancymaae* and that the first exon of *P. anubis* was poorly predicted due to poor genome coverage.

Thus the genomic region was collected and manually annotated in both species. This procedure revealed a number of ORF disrupting mutations in the last exon of *A. nancymaae GBA3* namely the loss of the canonical splice site acceptor AG>AC, followed by the insertion of 2bp, 2 premature stop codons and the insertion of 4 bp (see Figure S3A). We next sought to validate the identified mutations however several searches in SRA projects for *A. nancymaae GBA3* Exon 5 were unsuccessful retrieving no results. An alternative approach using *GBA3* Exon 5 sequence for tblastn searches in *A. nancymaae* whole genome retrieved 2 genomic scaffolds NW_018503959 (current assembly) and NW_012188438 (from previous genome assembly) non of which correspond to the current location of GBA3 in Gene-NCBI (NW_018511797.1). Gene annotation of the collected genomic scaffolds identified GBA3 exon 5 with no deleterious mutations in both cases thus full coding CDS was predicted and included in further analysis (Figure S3B)

Regarding *Papio anubis* although a 1bp deletion in exon 1 was detected in the collected genomic sequence (NC_018156.2 21400000-21676592) this mutation was not confirmed in SRA data (Figure S4A) thus GBA3 was considered as coding and the predicted CDS was included in further analysis (Figure S4B).

A

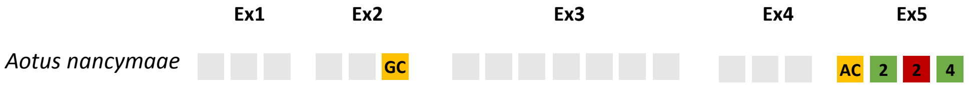

B

> ***Aotus nancymaae***_GBA3
ATGGCTTTCCCTGAGGGATTTGGATGGGCAGCGGCCACCGCAGCTTATCAAGTGGAAGGAGGCTGGGATGCAGATGGAAAAG
GCCCTTGTGTCTGGGACACATTTACTCATCAGGGAGGAGAGAGAGTTTTCAAGAACCAGACTGGCGATGTAGCTTGTGGCAG
CTACACTCTGTGGGAGGAAGATTTGAAATGTATCAAACAGCTTGGATTGACTCATTACCGCTTCTCTCTTTCCTGGTCACGT
CTGTTACCTGATGGGACGACAGGTTTCATCAACCAGAAAGGAATTGATTACTACAACAAGATCATCGACGATTTGTTAACAA
ATGGGGTTACTCCCATTGTGACACTCTACCACTTTGATTTACCTCAGGCTTTAGAAGACCAAGGAGGTTGGTTGTCAGAGGC
AATCATTGAATCATTTGACAAATATGCTCAGTTTTGCTTCAGTACCTTTGGGGATCGTGTTAAGCAGTGGATCACCATAAAT
GAAGCTAATATTCTTTCCCTGATGGCATATGACTTAGGTATCTTTCCTCCAGGTATCCCTCACTGTGGAACTGGAGGTTATC
AGGCAGCTCATAATTTGATTAAGGCTCATGCCAGATCCTGGCACAGCTATGATTCCTTATTTCGAAAAGAGCAGAAAGGTAT
GGTGTCCATATCACTTTTTGCGACCTGGTTGGAACCAGCAGATCCCAACTCAGTGTCTGACCAGGAAGCTGCTAAAAGAGCC
ATCACTTTCCATCTGGATTTATTTGCTAAACCCATATTCATTGATGGTGATTATCCTGAAGTTGTCAAATCTAAGATTGCCT
CTATGAGTCAAAGGCAAGGATATCCATCATCAAGGCTTCCGGAATTCACAGAAGAAGAGAAGAAAATGATCAAAGGCACTGC
TGATTTTTTTGCTGTGCAATATTATACAACTCGCTTAATCAAGTACCAGGAGAACAAGAAAGGAGAACTAGGCATTCTCCAG
GATGTGGAGATTGAATATTTTCCAGATCCATCTTGGAAAAATGTGGACTGGATCTATGTGGTACCATGGGGAATACGTAAAC
TACTGAAATATATTAAGGATACATATAATAACCCTGTAATTTATATTACTGAGAATGGGTTTCCCCAGAGTGACCCGGCACC
TCTTGATGACACTCAACGCTGGGAGCATTTCAGACAGACATTTCAGGAACTGCTCAAAGCGATCCAACTTGATAAAGTCAAT
CTTCAAGTATACTGTGCATGGTCTCTTCTGGATAACTTTGAGTGGAACCAAGGATACAGCAGCAGATTTGGTCTCTTCCACG
TCGATTTTGAAGACCCAGCTAGACCCCGAGTCCCTTACACATCGGCCAAGGAATATGCCAAGATCATCCGAAACAATGGCCT
AGAAGAACATCTGTAG

Figure S3: **A**-Schematic representation of the identified mutations during gene annotation of GBA3 in *Aotus nancymaae* using the available genome assembly in NCBI (Scaffold NW_018511797.1 Assembly GCF_000952055.2). Each group of grey squares represents an exon, number indicated above, yellow indicates loss of canonical splice site (AG-GT), green indicates insertion, blue indicates deletion and read indicates premature stop codon. Numbers in the squares indicate how many nucleotides were inserted or deleted or how many stop codons were identified.
B- Predicted GBA3 coding sequence obtained from scaffolds NW_018503959 and NW_012188438. Alternating colors (blue and black) indicate alternating exons.

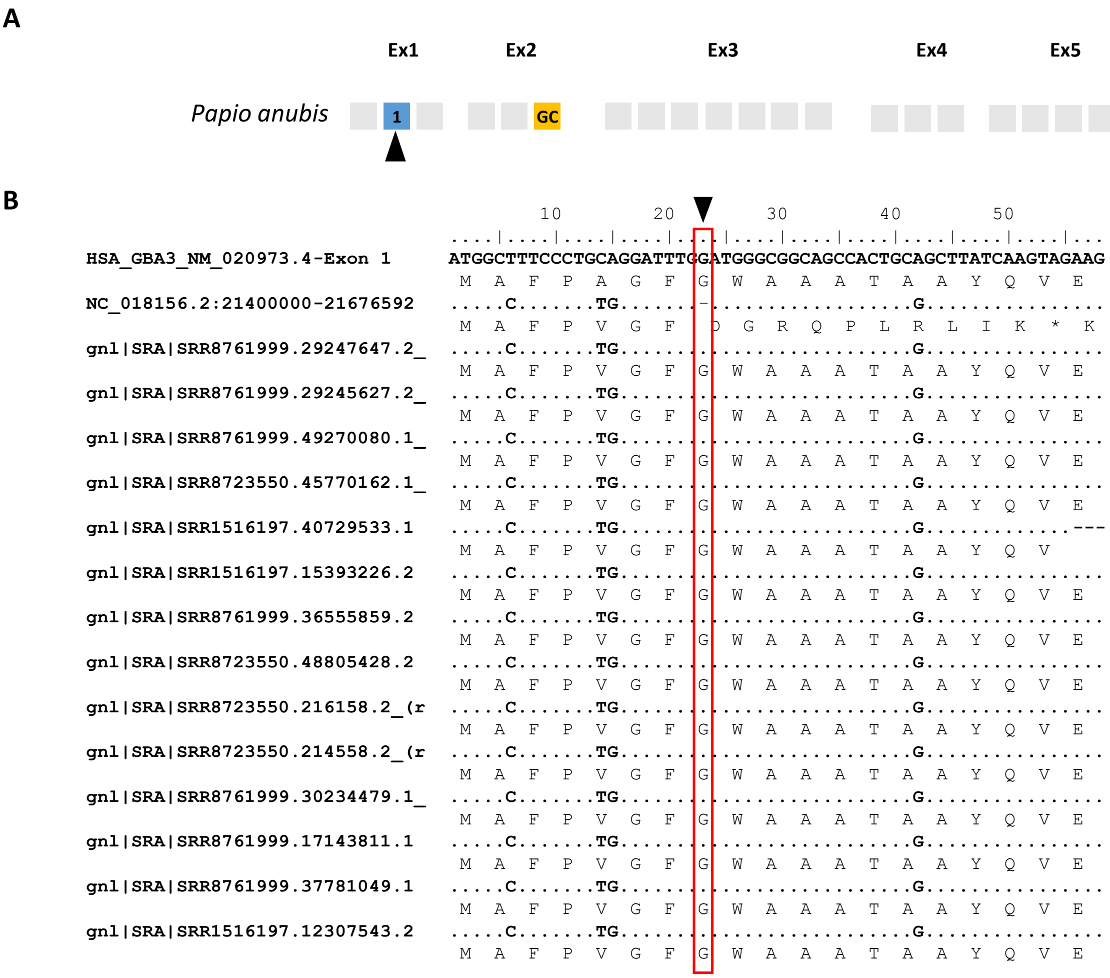

C

Project	Run	Submitted by	DATE	Sample
SRX653787	SRR1516197	Baylor College of Medicine (BCM)	2015-07-22	SAMN02866411 - LIV5 female
SRX5553583	SRR8761999	University of California, San Francisco	2019-03-21	SAMN11179045 –15944 male
SRX5516821	SRR8723550	University of California, San Francisco	2019-03-13	SAMN11119513 –12242 Female

D

```
>Papio anubis _GBA3
ATGGCCTTCCCTGTGGGATTTGGATGGGCGGCAGCCACTGCGGCTTATCAAGTAGAAGGAGGCTGGGATGCAGATGGAAAAGGCCC
TTGTGTCTGGGACACATTTACTCATCAGGGAGGAGAGAGAGTTTTCAAGAACCAGACTGGCGATGTAGCTTGTGGCAGCTACACTC
TGTGGGAGGAAGATTTGAAATGTATCAAACAGCTTGGATTGACTCATTACCGCTTCTCTCTTTCCTGGTCACGTCTGTTACCTGAT
GGGACGACAGGTTTCATCAACCAGAAAGGAATTGATTATTACAACAAGATCATTGATGATTTGTTAAAAAATGGGGTTACTCCCAT
CGTGACACTCTACCACTTTGATTTGCCTCAGGCTTTAGAAGACCGAGGAGGTTGGTTGTCAGAGTCTATCATTGAATCCTTTGACA
AATATGCTCAGTTTTGCTTCAGTACCTTTGGGGATCGTGTCAAGCAGTGGATCACCATAAATGAAGCTAATGTTCTTTCCGTGATG
GCATATGAGTTAGGTATGTTTCCTCCGGGTATCCCTCACTTTGGAACTGGAGGTTATCAGGCAGCTCATAATTTGATTAAGGCTCA
TGCCAGATCCTGGCACAGCTATGATTCCCTGTTTCGAAAACAGCAGAAAGGTATGGTGTCCCTATCACTTTTTGCGGTCTGGTTGG
AACCTGCAGATCCCAACTCAGTGTCTGACCAGGAAGCTGCTAAAAGAGCCATCAAATTCCATCTGGATTTCTTTGCTAAACCCATA
TTCATCGATGGTGATTATCCTGAAGTTGTCAAGTCTCAGATTGCCTCCATGAGTCAAAAGCAAGGCTATCCATCATCGAGGCTTCC
AGAATTCGCTGAAGAAGAGAAGAAAATGATCAAAGGCACTGCTGATTTTTTTGCTGTGCAATATTATACAACTCGCTTAATCAAAT
ACCAGGAGAACAAGAAAGGAGAATTAGGTATTCTCCAGGATGCGGAAATTGAATTTTTTCCAGATCCATCTTGGAAAAATGTGGAT
TGGATTTATGTGGTACCATGGGGAGTACGTAAACTACTGAAGTATATTAAGGATACATATAATAACCCTGTAATTTATATCACTGA
GAATGGGTTTCCCCAGAGTGACCCAGCGCCTCTTGATGACACTCAACGCTGGGAGCATTTCAGACAGACATTTCAGGAACTGTTCA
AAGCTATCCAACTTGATAAAGTCAATCTTCAAGTATATTGTGCGTGGTCTCTTCTGGATAACTTTGAGTGGAACCAGGGATACAGC
AGCCGGTTTGGTCTCTTCCATGTTGATTTTGAAGACCCAGCTAGACCCCGAGTCCCTTACACATCAGCCAAGGAATATGCCAAGAT
CATCCGAAACAATGGCCTTGAAGCACATCTGTAG
```

Figure S4: **A**-Schematic representation of the identified mutations during gene annotation of GBA3 in *Papio Anubis* using the available genome assembly in NCBI (Scaffold XM_021938915.1 Assembly GCF_000264685.3). Each group of grey squares represents an exon, number indicated above, yellow indicates loss of canonical splice site (AG-GT), green indicates insertion, blue indicates deletion and read indicates premature stop codon. Numbers in the squares indicate how many nucleotides were inserted or deleted or how many stop codons were identified. **B**-Validation of the identified mutation through multiple sequence alignment of reads obtained from 3 SRA projects. Red box highlights the location of the expected 1 nucleotide deletion which was not observed in the collected reads. **C**-SRA projects consulted. **D**- Predicted GBA3 coding sequence obtained from scaffolds XM_021938915.1 and SRA data. Alternating colours (blue and black) indicate alternating exons.

Regarding the primates *Piliocolobus tephrosceles* and *Callithrix jacchus*, manual annotation uncovered a 1bp insertion in exon 3 followed by a 2bp deletion in exon 4 and the loss of the canonical acceptor splice site in exon 5 AG>TG in *Piliocolobus tephrosceles* (Figure S5). SRA search and validation of mutations found in *P. tephrosceles* retrieved no results, while blastn searches in the available whole genome assemblies ASM277652V2 (current assembly) and ASM277652v1 (previous) both obtain from the same individual recovered the same region previously annotated. Thus the identified mutations in *Piliocolobus tephrosceles GBA3* gene were not validated, and GBA3 coding status remained undetermined.

For *Callithrix jacchus* manual annotation was unsuccessful in identifying the 5´region of exon 5 due to poor genome coverage, however a 5bp insertion was identified in the remaining exon 5 (FigureS6A), yet SRA validation did not confirm the identified mutations (Figure S6 B and C) and predicted CDS was included in further analysis (Figure S6D) .

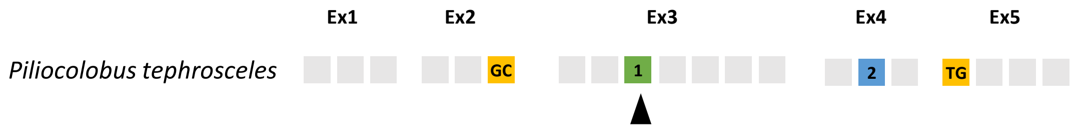

Figure S5: Schematic representation of the identified mutations during gene annotation of GBA3 in *Papio Anubis* using the available genome assembly in NCBI (Scaffold NW_020556090.1 Assembly ASM277652V2). Each group of grey squares represents an exon, number indicated above, yellow indicates loss of canonical splice site (AG-GT), green indicates insertion, blue indicates deletion and read indicates premature stop codon. Numbers in the squares indicate how many nucleotides were inserted or deleted or how many stop codons were identified..

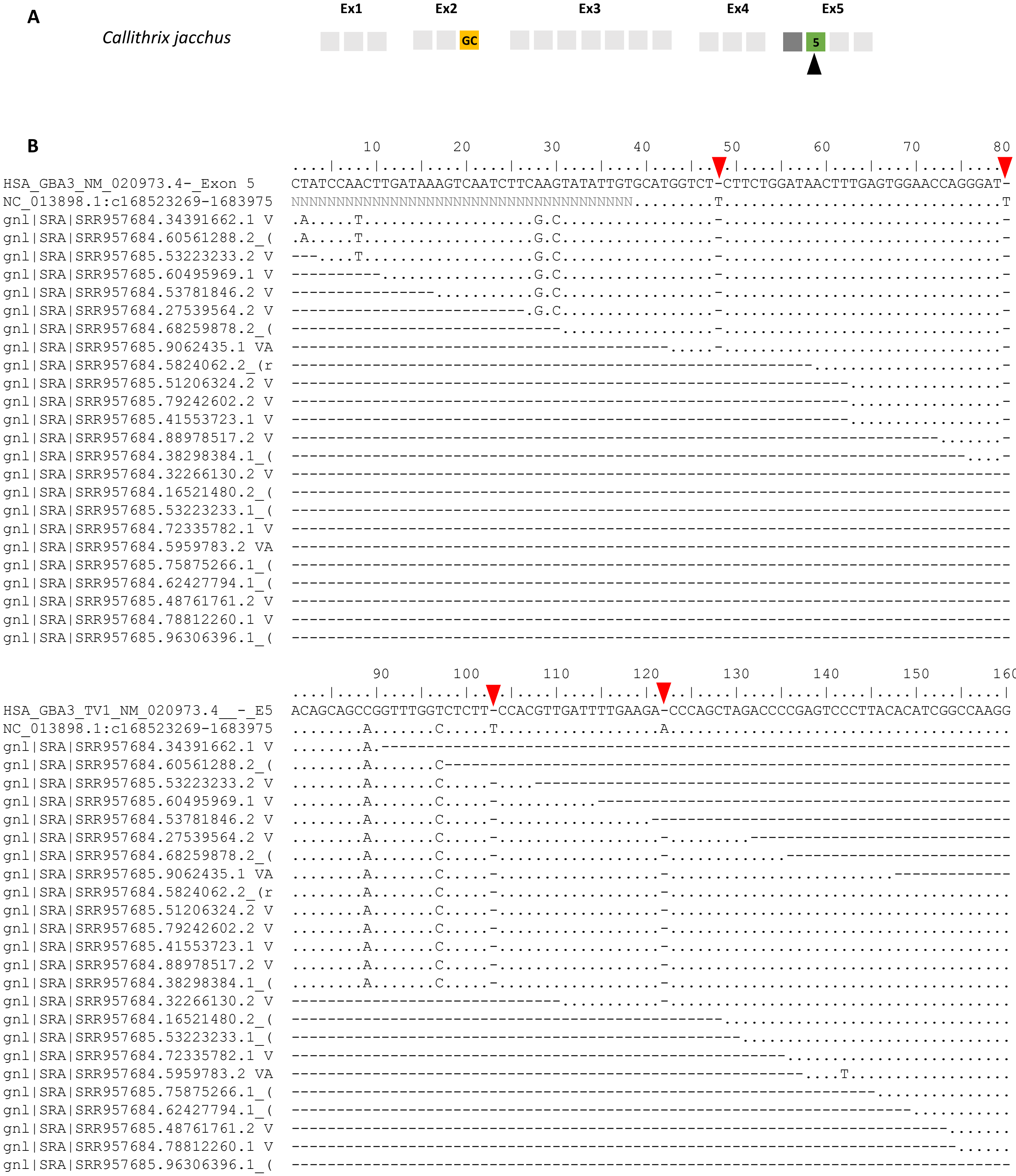
A
Callithrix jacchus
Ex1
Ex2
Ex3
Ex4
Ex5
GC
5
B
10 20 30 40 50 60 70 80
HSA_GBA3_NM_020973.4-_Exon 5
CTATCCAACTTGATAAAGTCAATCTTCAAGTATATTGTGCATGGTCT-CTTCTGGATAACTTTGAGTGGAACCAGGGAT-
NC_013898.1:c168523269-1683975
gnl|SRA|SRR957684.34391662.1 V
gnl|SRA|SRR957684.60561288.2_(
gnl|SRA|SRR957685.53223233.2 V
gnl|SRA|SRR957685.60495969.1 V
gnl|SRA|SRR957684.53781846.2 V
gnl|SRA|SRR957684.27539564.2 V
gnl|SRA|SRR957684.68259878.2_(
gnl|SRA|SRR957685.9062435.1 VA
gnl|SRA|SRR957684.5824062.2_(r
gnl|SRA|SRR957685.51206324.2 V
gnl|SRA|SRR957685.79242602.2 V
gnl|SRA|SRR957685.41553723.1 V
gnl|SRA|SRR957684.88978517.2 V
gnl|SRA|SRR957684.38298384.1_(
gnl|SRA|SRR957684.32266130.2 V
gnl|SRA|SRR957684.16521480.2_(
gnl|SRA|SRR957685.53223233.1_(
gnl|SRA|SRR957684.72335782.1 V
gnl|SRA|SRR957684.5959783.2 VA
gnl|SRA|SRR957685.75875266.1_(
gnl|SRA|SRR957684.62427794.1_(
gnl|SRA|SRR957685.48761761.2 V
gnl|SRA|SRR957684.78812260.1 V
gnl|SRA|SRR957685.96306396.1_(
90 100 110 120 130 140 150 160
HSA_GBA3_TV1_NM_020973.4__-_E5
ACAGCAGCCGGTTTGGTCTCTT-CCACGTTGATTTTGAAGA-CCCAGCTAGACCCCGAGTCCCTTACACATCGGCCAAGG
NC_013898.1:c168523269-1683975

```
                                        170       180       190       200
                                ....|....|....|....|....|....|....|....|....|...
HSA_GBA3_TV1_NM_020973.4__-_E5  AA-TATGCCAAGATCATCCGAAACAATGGCCTTGAAGCACATCTGTAG
NC_013898.1:c168523269-1683975  ..A............G................A....A......A...
gnl|SRA|SRR957684.34391662.1 V  ------------------------------------------------
gnl|SRA|SRR957684.60561288.2_(  ------------------------------------------------
gnl|SRA|SRR957685.53223233.2 V  ------------------------------------------------
gnl|SRA|SRR957685.60495969.1 V  ------------------------------------------------
gnl|SRA|SRR957684.53781846.2 V  ------------------------------------------------
gnl|SRA|SRR957684.27539564.2 V  ------------------------------------------------
gnl|SRA|SRR957684.68259878.2_(  ------------------------------------------------
gnl|SRA|SRR957685.9062435.1 VA  ------------------------------------------------
gnl|SRA|SRR957684.5824062.2_(r  ..----------------------------------------------
gnl|SRA|SRR957685.51206324.2 V  ..-....-----------------------------------------
gnl|SRA|SRR957685.79242602.2 V  ..-....-----------------------------------------
gnl|SRA|SRR957685.41553723.1 V  ..-....-----------------------------------------
gnl|SRA|SRR957684.88978517.2 V  ..-............G.-------------------------------
gnl|SRA|SRR957684.38298384.1_(  ..-............G....----------------------------
gnl|SRA|SRR957684.32266130.2 V  ..-............G................A....A......A...
gnl|SRA|SRR957684.16521480.2_(  ..-............G................A....A......A...
gnl|SRA|SRR957685.53223233.1_(  ..-............G................A....A......A...
gnl|SRA|SRR957684.72335782.1 V  ..-............G................A....A......A...
gnl|SRA|SRR957684.5959783.2 VA  ..-............G................A....A......A...
gnl|SRA|SRR957685.75875266.1_(  ..-............G................A....A......A...
gnl|SRA|SRR957684.62427794.1_(  ..-............G................A....A......A...
gnl|SRA|SRR957685.48761761.2 V  ..-............G................A....A......A...
gnl|SRA|SRR957684.78812260.1 V  ..-............G................A....A......A...
gnl|SRA|SRR957685.96306396.1_(  -------------..G................A....A......A...
```

C

Project	Run	Submitted by	DATE	Sample
SRX339467	SRR957685	LAUSANNE UNIVERSITY, CIG, GÉNOPODE	2014-04-23	SAMN02333822 female
SRX339466	SRR957684	LAUSANNE UNIVERSITY, CIG, GÉNOPODE	2014-04-23	SAMN02333821 male

D

```
>Callithrix jacchus_GBA3
ATGGCTTTCCCTGAGGGATTTGGATGGGCGGCAGCCACCGCAGCTTATCAAGTGGAAGGAGGCTGGGATGCAGATGGAAAAGGGCCTTGTGTCTGGGACACAT
TTACTCATCAGGGAGGAGAGAGAGTTTTCAAGAACCAGACTGGCGATGTAGCTTGTGGCAGCTACACTCTGTGGGAGGAAGATTTGAAATGTATCAAACAGCT
TGGATTGACTCATTACCGCTTCTCTCTTTCCTGGTCACGTCTGTTACCTGATGGGACGACAGGTTTCATCAACCAGAAAGGAATTGATTATTACAACAAGATC
ATCGACGATTTGTTAACAAATGGGGTTACTCCCATTGTGACACTCTACCACTTTGATTTACCTCAGGCTTTAGAAGACCAAGGAGGTTGGTTGTCAGAGGCAA
TCATTGAATTCTTTGACAAATATGCTCAGTTTTGCTTCAGTACTTTTGGGGATCGTGTCAAGCAGTGGATCACCATAAATGAAGCTAATATTCTTTCCATGAT
GGCATATGACTTAGGTATCTTTCCTCCAGGTGTCCCTCACTGTGGAACAGGAGGTTATCAGGCAGCTCATAATTTGATTAAGGCTCATGCCAGATCCTGGCAC
AGCTATGATTCCTTATTTCGAAAAGAGCAGAAAGGTCTGGTGTCCCTATCACTTTTTGCGACCTGGTTGGAACCAGCAGATCCAAACTCAGTATCTGACCAGG
AAGCTGCTAAAAGAGCCATCACTTTCCATCTGGATTTCTTTGCTAAACCCATATTCATTGATGGTGATTATCCTGAAGTTGTCAAGTCTCAGATTGCCTCCAT
GAGTCAAAAGCAAGGGTATCCATCGTCAAGGCTTCCGGAATTCACAGAAGAAGAGAAGAAAATGATCAAAGGCACTGCTGATTTTTTTGCTGTGCAATATTAT
ACAACTCGTTTAATCAAGTACCAGGAGAACAGGAAAGGAGAACTAGGCATTCTCCAGGATGTGGAGATTGAATTTTTTCCAGATCCATCTTGGAAAAATGTGG
ACTGGATCTATGTGGTACCATGGGGAGTACGTAAGCTACTGAAATATATTAAGGATACATATAATAACCCTGTAATTTATATCACTGAGAATGGATTTCCCCA
GAGTGACCCGGCACCTCTTGATGACACTCAACGCTGGGAGTATTTCAGACAGACATTTCAGGAACTGCTCAAAGCAATCCATCTTGATAAAGTCAATCTTCGA
CTATATTGTGCATGGTCTCTTCTGGATAACTTTGAGTGGAACCAGGGATACAGCAGCAGGTTTGGCCTCTTCCACGTTGATTTTGAAGACCCAGCTAGACCCC
GAGTCCCTTACACATCGGCCAAGGAATATGCCAAGATCGTCCGAAACAATGGCCTAGAAGAACATCTATAG
```

Figure S6: **A**-Schematic representation of the identified mutations during gene annotation of GBA3 in *Callithrix jacchus* using the available genome assembly in NCBI (Scaffold NC_013898.1 Assembly GCA_000004665.1). Each group of grey squares represents an exon, number indicated above, yellow indicates loss of canonical splice site (AG-GT), green indicates insertion, blue indicates deletion and read indicates premature stop codon. Numbers in the squares indicate how many nucleotides were inserted or deleted or how many stop codons were identified. **B**-Validation of the identified mutation through multiple sequence alignment of reads obtained from 2 SRA projects. Red arrows highlights the location of the expected 1 nucleotide insertions (total of 5) which where not observed in the collected reads. **C**-SRA projects consulted. **D**- Predicted GBA3 coding sequence obtained from scaffold NC_013898.1 and SRA data. Alternating colours (blue and black) indicate alternating exons.

SUPPLEMENTARY MATERIAL- 3

Supplementary Material 3 - Validation of the identified mutations in Rodentia

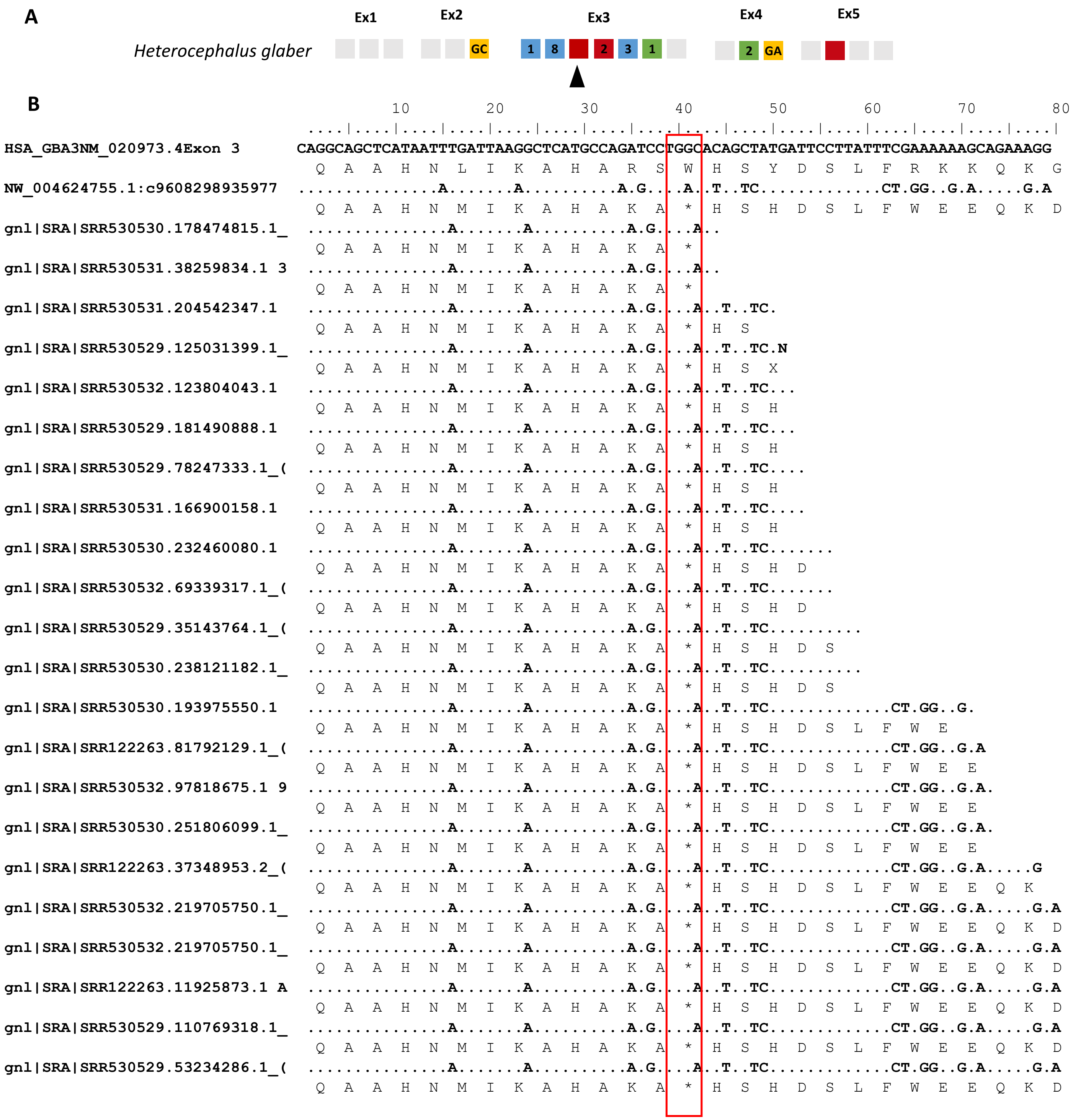

C

Project	Run	Submitted by	DATE	Sample
SRX171625	SRR530532	Broad Institute (BI)	20120731	SAMN00714537 NMR29 female
SRX171625	SRR530531	Broad Institute (BI)	20120731	SAMN00714537 NMR29 female
SRX171625	SRR530530	Broad Institute (BI)	20120731	SAMN00714537 NMR29 female
SRX171625	SRR530529	Broad Institute (BI)	20120731	SAMN00714537 NMR29 female
SRX047034	SRR122263	Beijing Genome Institute (BGI)	20111012	SAMN00217190 BGINMRgDNA male

Figure S7: **A**-Schematic representation of the identified mutations during gene annotation of GBA3 in *Heterocephalus glaber* using the available genome assembly in NCBI (Scaffold NW_004624755.1). Each group of grey squares represents an exon, number indicated above, yellow indicates loss of canonical splice site (AG-GT), green indicates insertion, blue indicates deletion and read indicates premature stop codon. Numbers in the squares indicate how many nucleotides were inserted or deleted or how many stop codons were identified. **B**-Validation of the identified mutation through multiple sequence alignment of reads obtained from 2 SRA projects. Red box highlights the location of the premature stop codon. **C**-SRA projects consulted.

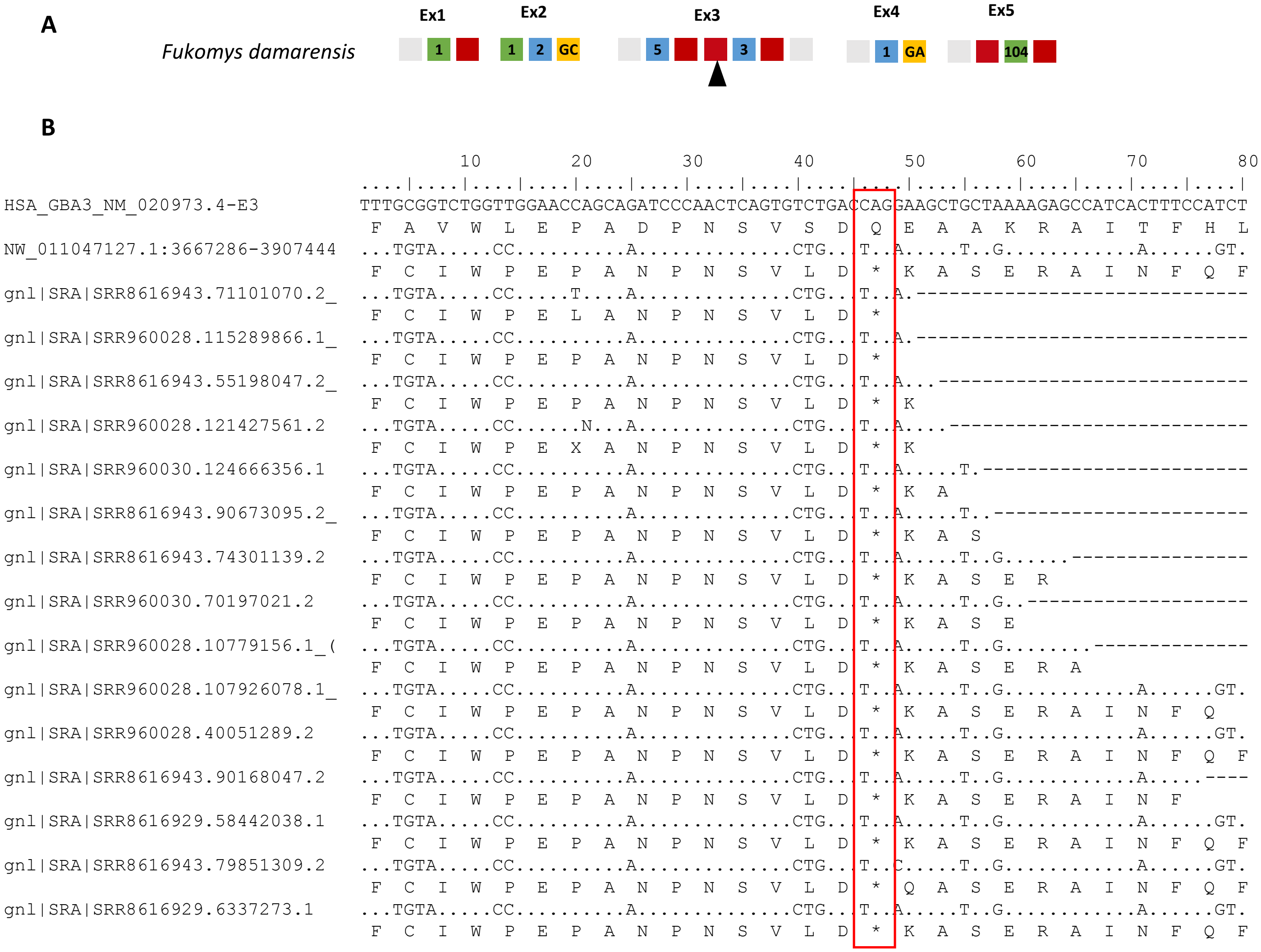

C

Project	Run	Submitted by	DATE	Sample
SRX5415846	SRR8616943	Baylor College of Medicine	2019-02-24	SAMN109737241036 female
SRX5415860	SRR8616929	Baylor College of Medicine	2019-02-24	SAMN109737241036 female
SRX341884	SRR960028	Beijing Genome Institute	2015-07-22	SAMN02339281
SRX341884	SRR960030	Beijing Genome Institute	2015-07-22	SAMN02339281

Figure S8: **A**-Schematic representation of the identified mutations during gene annotation of GBA3 in *Fukomys damarensis* using the available genome assembly in NCBI (Scaffold NW_011047127.1). Each group of grey squares represents an exon, number indicated above, yellow indicates loss of canonical splice site (AG-GT), green indicates insertion, blue indicates deletion and read indicates premature stop codon. Numbers in the squares indicate how many nucleotides were inserted or deleted or how many stop codons were identified. **B**-Validation of the identified mutation through multiple sequence alignment of reads obtained from 2 SRA projects. Red box highlights the location of the premature stop codon. **C**-SRA projects consulted.

A

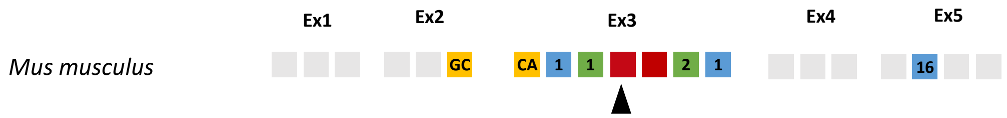

B

```
                                  10        20        30        40        50        60        70        80
                              ....|....|....|....|....|....|....|....|....|....|....|....|....|....|....|....|
HSA_GBA3_NM_020973.4_-_Exon 3 CATG-CCAGATCCTGG-CACAGCTA-TGATTCCTTATTTCGAAAAAAGCAGAAAGGTATGGTGTCTC-TATCACTTTTTG
                               H  A  R  S  W  H  S  Y  D  S  L  F  R  K  K  Q  K  G  M  V  S  L  S  L  F
NC_000071.6:c51058996-49659951 ....-T..........-........-......T.....CT..G..G.......G..CT.T..C..C.-.G...T.A...T
                               H  V  R  S  W  H  S  Y  D  S  L  F  *  E  E  Q  K  G  F  V  S  L  S  L  F
ti:9239033 G10P610156RE1.T0     ....-T..T.......-........-......T.....CT..T..TT-..T.....CT.T..C..C.-.G...T.A...T
                               H  V  I  S  W  H  S  Y  D  S  L  F  *  *  F  I  K  A  L  S  P  C  H  Y  F
ti:1018826122_(reversed) 19866 ....-...........-........-......T.....CT..G..G.......G..CT.T..C..C.-.G...T.A...T
                               H  A  R  S  W  H  S  Y  D  S  L  F  *  E  E  Q  K  G  F  V  S  L  S  L  F
ti:1102395782_(reversed) 19866 ....-...........-........-......T.....CT..G..G.......G..CT.T..C..C.-.G...T.A...T
                               H  A  R  S  W  H  S  Y  D  S  L  F  *  E  E  Q  K  G  F  V  S  L  S  L  F
ti:1035355973 19866846494073   ....-...........-........-......T.....CT..G..G.......G..CT.T..C..C.-.G...T.A...T
                               H  A  R  S  W  H  S  Y  D  S  L  F  *  E  E  Q  K  G  F  V  S  L  S  L  F
ti:37386555_(reversed) G10P646 ....-T......T...-........-.....AT......T..G..G.......G..CT.T..C..C.-.G...T.A...T
                               H  V  R  S  W  H  S  Y  D  Y  L  F  *  E  E  Q  K  G  F  V  S  L  S  L  F
ti:37324480_(reversed) G10P646 ....-T......T...-........-.....AT......T..G..G.......G..CT.T..C..C.-.G...T.A...T
                               H  V  R  S  W  H  S  Y  D  Y  L  F  *  E  E  Q  K  G  F  V  S  L  S  L  F
ti:48745085_(reversed) kpx24d1 ....-T..........-........-......T.....CT..G..G.......G..CT.T..C..C.-.G...T.A...T
                               H  V  R  S  W  H  S  Y  D  S  L  F  *  E  E  Q  K  G  F  V  S  L  S  L  F
ti:44708880 G10P672563FE7.T0   ....-T..........-........-......T.....CT..G..G.......G..CT.T..C..C.-.G...T.A...T
                               H  V  R  S  W  H  S  Y  D  S  L  F  *  E  E  Q  K  G  F  V  S  L  S  L  F
ti:87523555_(reversed) lzt96h0 ....-T..........-........-......T.....CT..G..G.......G..CT.T..C..C.-.G...T.A...T
                               H  V  R  S  W  H  S  Y  D  S  L  F  *  E  E  Q  K  G  F  V  S  L  S  L  F
ti:1092365262 19866832669217 s ....-...........-........-......T.....CT..G..G.......G..CT.T..C..C.-.G...T.A...T
                               H  A  R  S  W  H  S  Y  D  S  L  F  *  E  E  Q  K  G  F  V  S  L  S  L  F
ti:80289841 lmt78f06.b1        ....-T..........-........-......T.....CT..G..G.......G..CT.T..C..C.-.G...T.A...T
                               H  V  R  S  W  H  S  Y  D  S  L  F  *  E  E  Q  K  G  F  V  S  L  S  L  F
ti:1038747621 19866849120478 s ....-T..........-........-......T.....CT..G..G.......G..CT.T..C..C.-.G...T.A...T
                               H  V  R  S  W  H  S  Y  D  S  L  F  *  E  E  Q  K  G  F  V  S  L  S  L  F
ti:1099175474 19866828235050 s ....-...........-........-......T.....CT..G..G.......G..CT.T..C..C.-.G...T.A...T
                               H  A  R  S  W  H  S  Y  D  S  L  F  *  E  E  Q  K  G  F  V  S  L  S  L  F
ti:1030514705 19866847738048   ....-T..........-........-......T.....CT..G..G.......G..CT.T..C..C.-.G...T.A...T
                               H  V  R  S  W  H  S  Y  D  S  L  F  *  E  E  Q  K  G  F  V  S  L  S  L  F
ti:54470615_(reversed) G10P671 ....-T..........-........-......T.....CT..G..G.......G..CT.T..C..C.-.G...T.A...T
                               H  V  R  S  W  H  S  Y  D  S  L  F  *  E  E  Q  K  G  F  V  S  L  S  L  F
ti:692448_(reversed) mk2A-a317 ....-T..........-........-......T.....CT..G..G.......G..CT.T..C..C.-.G...T.A...T
                               H  V  R  S  W  H  S  Y  D  S  L  F  *  E  E  Q  K  G  F  V  S  L  S  L  F
```

C

Trace archive search	Submitted by	DATE
ti:1071293497	CRA	2005-12-07
ti:1071293497	MWGA	2001-10-04
ti:1118280633	CRA	2006-01-11
ti:692448	SC	2003-01-03
ti: 54470615	BI	2001-10-10
ti:1030514705	CRA	2005-11-05
ti: 1099175474	CRA	2005-12-28
ti: 1038747621	CRA	2005-11-13
ti: 80289841	WUGSC	2001-12-27
ti: 1092365262	CRA	2005-12-24

Figure S9: **A**-Schematic representation of the identified mutations during gene annotation of GBA3 in *Mus musculus* using the available genome assembly in NCBI (Scaffold NC_000071.6). Each group of grey squares represents an exon, number indicated above, yellow indicates loss of canonical splice site (AG-GT), green indicates insertion, blue indicates deletion and read indicates premature stop codon. Numbers in the squares indicate how many nucleotides were inserted or deleted or how many stop codons were identified. **B**-Validation of the identified mutation through multiple sequence alignment of reads obtained from NCBI trace archive projects. Red box highlights the location of the premature stop codon. **C-**Reference numbers of Trace archives searched.

Rodentia pseudogenes predicted CDS

>*Mus musculus*_PSEUDO_GBA3
ATGGCTTTCCCTGTGGGGTTGGGATGGGGAGCAGCCACTGCTGCATATCAAGTAGAAGGAGGCTGGGATGCAGATGGAAGAGGCCCT
TGTGCCTGGGACACATTCACTCATCAGGGAGGTGAGCGAGATTTTGAGGACCAGACTGGTGATGTAGCTTGTGGCAGCTACACTCTG
TGGGAAGAAGATGTGAAATGTATCAAACAGCTTGGATTGACTCATTACCGTTTCTCTCTTTCCTGGTCACGTCTGTTACCTGATGGG
ACGACAGGTTTCATCAACCAGAAAGAAATTGACTATTACAACAAGATCAAGATGATTTGTTAAGGAATGGGGTGACGCCCATAGTGG
TCATCTATCACTTTGGTTTGTCTCGGGCTCTAGAAGACCAAGGAGATTGGTTGTCGGAATCAATTGTTGAAGCCTTTGACAGATATG
CTCAGTTTTGCTTTGGCACCTTTGGAGACCTAGTGAAGCAGTGGATCACCATAAATGAGCCTAATATTCTTGCTCTTTTGGCCTACA
ACATGGGTATCTTTTCTCCTGTTGTGTCTCATGTTGGGATTGGGGGCTTATCAAGCAGATCATAATTTGATTAAGGCTCATGTCAGA
TCCTGGCACAGCTATGATTCTTTATTCTGAGAAGAGCAGAAGGGCTTTGTCTCCCTGTCATTATTTTTTTGCTGGTTAGAACCAGCA
GAGCCCCACTCAGAGATTGGCCAGGAAGCTACTAAGAGGGCCATCAATTTCCACTTGGATTTCTTTGCCAAACCCATATTTATTGAT
GGCGACTATCCAGATGTTGTCAAGTCCCAGGTTACCCCCATGAGCAAAAAGCAAGACGATCCATCATCAAGGCTTCCAGGATCCACA
GAAGAAGAGAAGAAAATGATCAAAGGCACTGCTGATTTCTTTGCTGTTAAATATCATCCAACTCCCATAGTCAGGCATCAGTAGAAT
AAGAAGAGAGAACTGAGCTTTCTTCAGGATGTGGAGATTGAATTTTTTTTCCAATCCATCTTGGAAAAATGTGGGTTGGATATATAT
GGTGCCATGGGGAATATGTAAATTACTGAGATATATTAAAGATACATATAATAACCCTGTAATTTACATCACTGAGAATGGGTTTCC
CCAGTGTGACCCCCCATCTCTTGATGACACTCAGCGATGGGAGTATTTCAGACAGACATTTCAGGAACTATTCAAAGCTATCCATAT
TGATGGATTCAATCTTCAAGTATACTGGATAATTTTGAGTGGAACAATAGATACCACAGACTGTTTGATCCCTCCCATGTTGATTTT
GAAGATCCTGCTAGAACCAGTGCTCCATACAGGGCAGCCAAGGAATATGCCAAGGTCATCAGAAACAATGGCCTGGAAGACACTGTG
TAG

Supplementary material- 4

Supplementary Material 4-Validation of the identified mutations in Cetacea

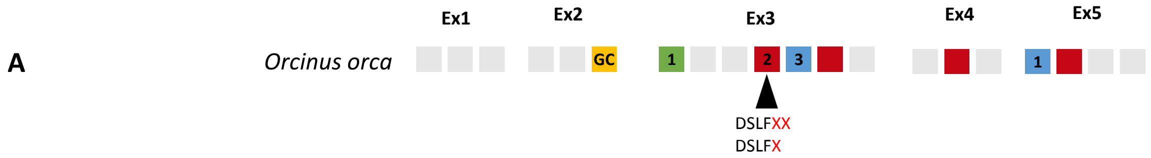

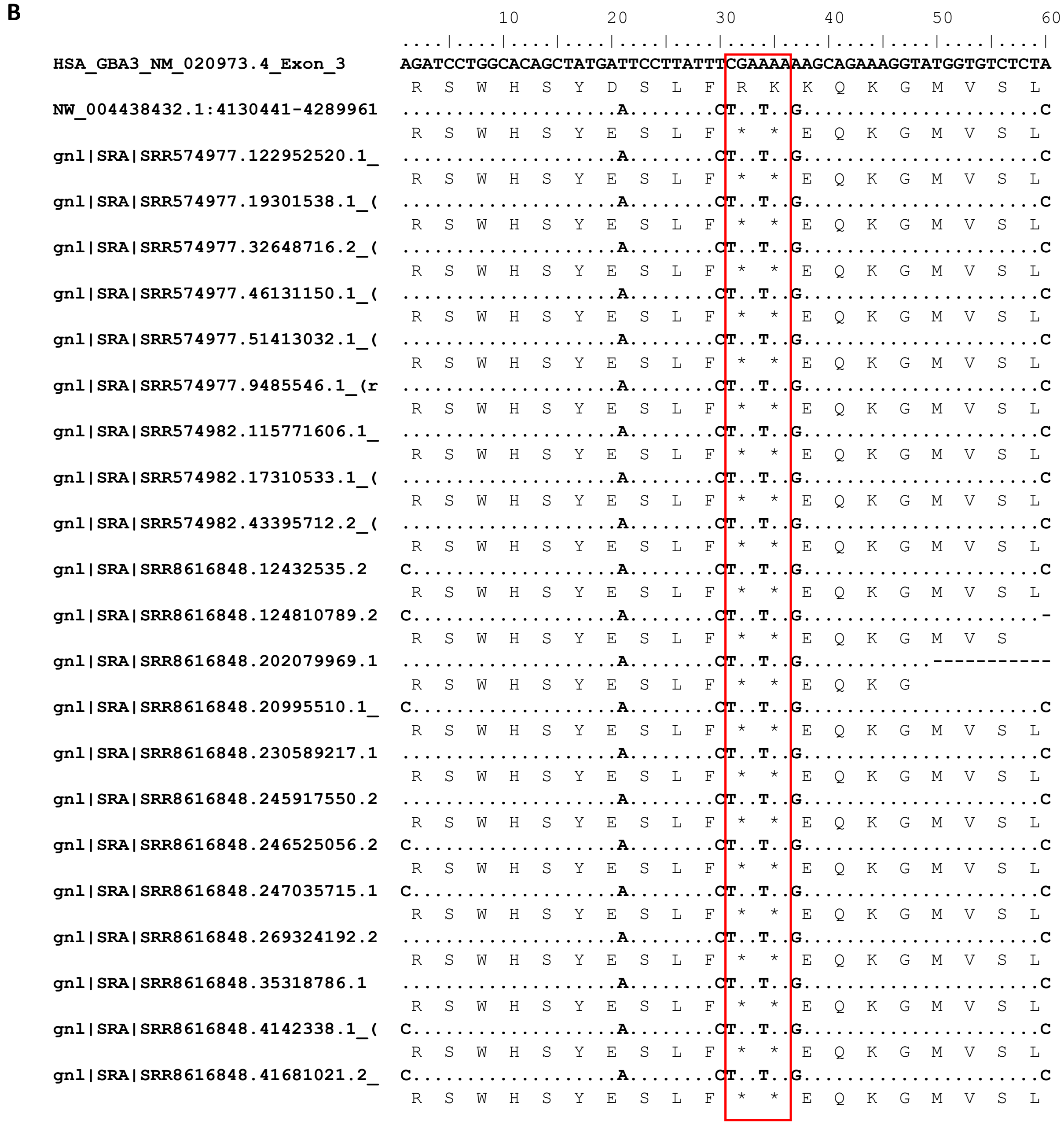

C

Project	Run	Submitted by	DATE	Sample
SRX188934	SRR574977	Baylor College of Medicine	2012-09-20	SAMN01180276 AForca1 female
SRX188934	SRR574982	Baylor College of Medicine	2012-09-20	SAMN01180276 AForca1 female
SRX5415941	SRR8616848	Baylor College of Medicine	2019-02-24	SAMN10973740 0193 female

Figure S10: **A**-Schematic representation of the identified mutations during gene annotation of GBA3 in *Orcinus orca* using the available genome assembly in NCBI (Scaffold NW_004438432.1). Each group of grey squares represents an exon, number indicated above, yellow indicates loss of canonical splice site (AG-GT), green indicates insertion, blue indicates deletion and read indicates premature stop codon. Numbers in the squares indicate how many nucleotides were inserted or deleted or how many stop codons were identified. **B**-Validation of the identified mutation through multiple sequence alignment of reads obtained from SRA projects. Red box highlights the location of the premature stop codon. **C**-SRA projects consulted.

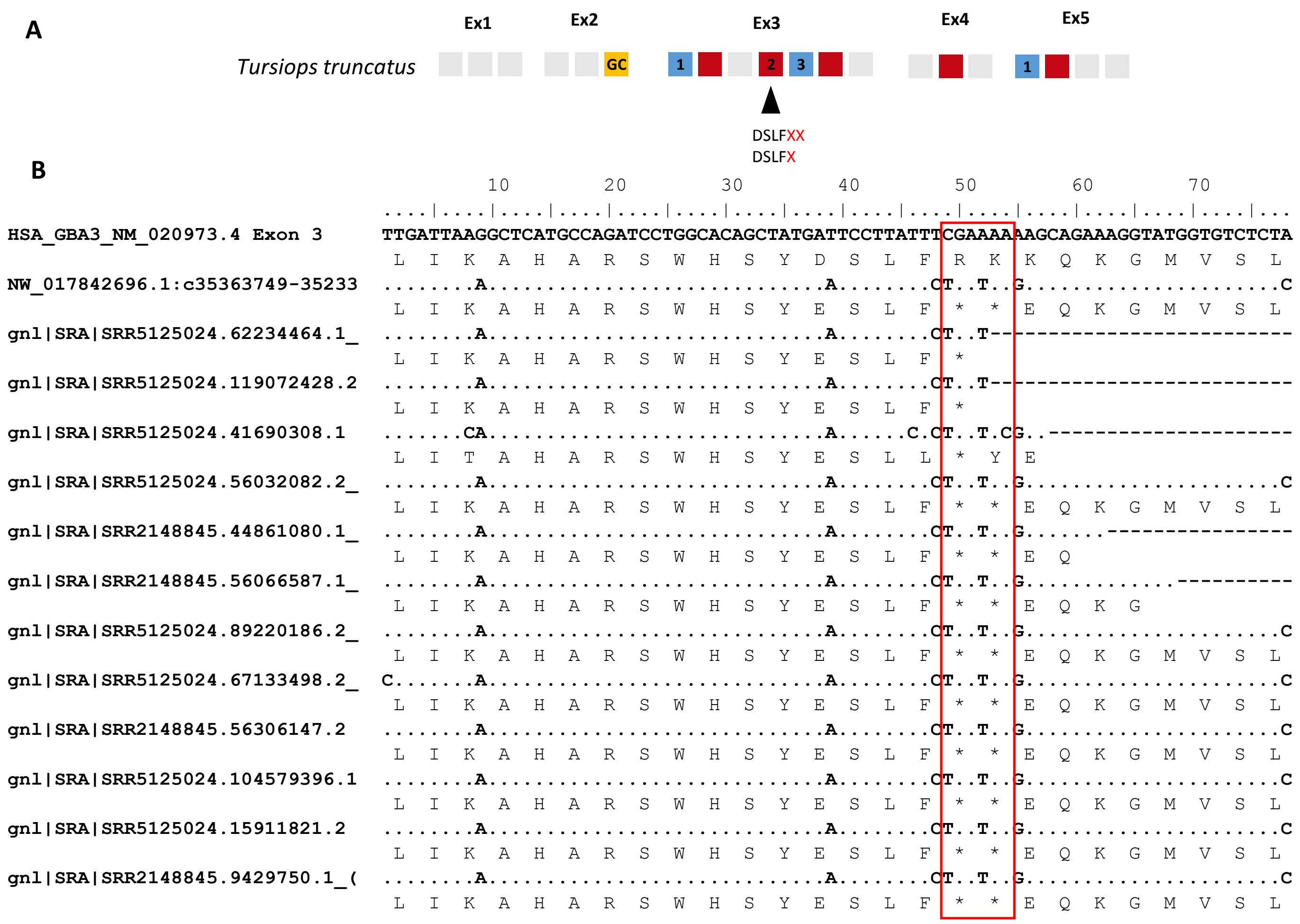

C

Project	Run	Submitted by	DATE	Sample
SRX1136400	SRR2148845	Beijing Genome Institute	2016-08-10	SAMN03968479
SRX2439826	SRR5125024	National Institute of Standards and Technology	2016-12-27	SAMN06114300 ACU285439A female

Figure S11: **A**-Schematic representation of the identified mutations during gene annotation of GBA3 in *Tursiops truncatus* using the available genome assembly in NCBI (Scaffold NW_017842696.1). Each group of grey squares represents an exon, number indicated above, yellow indicates loss of canonical splice site (AG-GT), green indicates insertion, blue indicates deletion and read indicates premature stop codon. Numbers in the squares indicate how many nucleotides were inserted or deleted or how many stop codons were identified. **B**-Validation of the identified mutation through multiple sequence alignment of reads obtained from SRA projects. Red box highlights the location of the premature stop codon. **C**-SRA projects consulted.

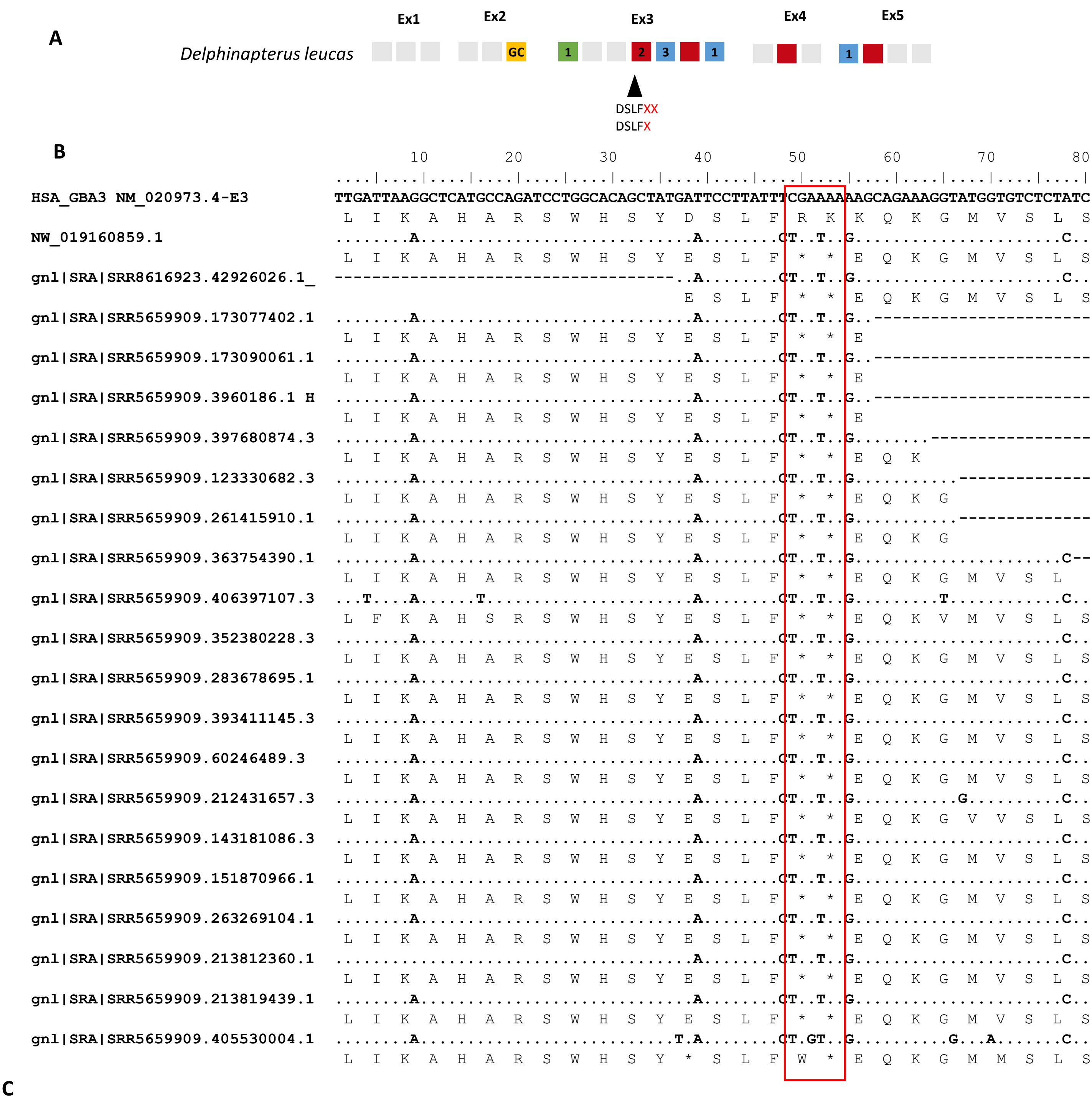

C

Project	Run	Submitted by	DATE	Sample
SRX2896241	SRR5659909	BC Cancer Agency Michael Smith Genome Sciences Centre	2017-06-27	SAMN06216270 SJ_33 female
SRX5415866	SRR8616923	Baylor College of Medicine	2019-02-24	SAMN10973720 0237 female

Figure S12: **A**-Schematic representation of the identified mutations during gene annotation of GBA3 in *Delphinapterus leucas* using the available genome assembly in NCBI (Scaffold NW_019160859.1). Each group of grey squares represents an exon, number indicated above, yellow indicates loss of canonical splice site (AG-GT), green indicates insertion, blue indicates deletion and read indicates premature stop codon. Numbers in the squares indicate how many nucleotides were inserted or deleted or how many stop codons were identified. **B**-Validation of the identified mutation through multiple sequence alignment of reads obtained from SRA projects. Red box highlights the location of the premature stop codon. **C**-SRA projects consulted.

A

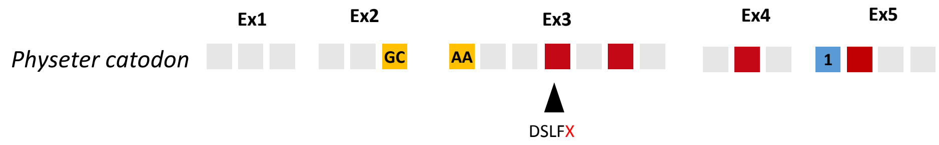

B

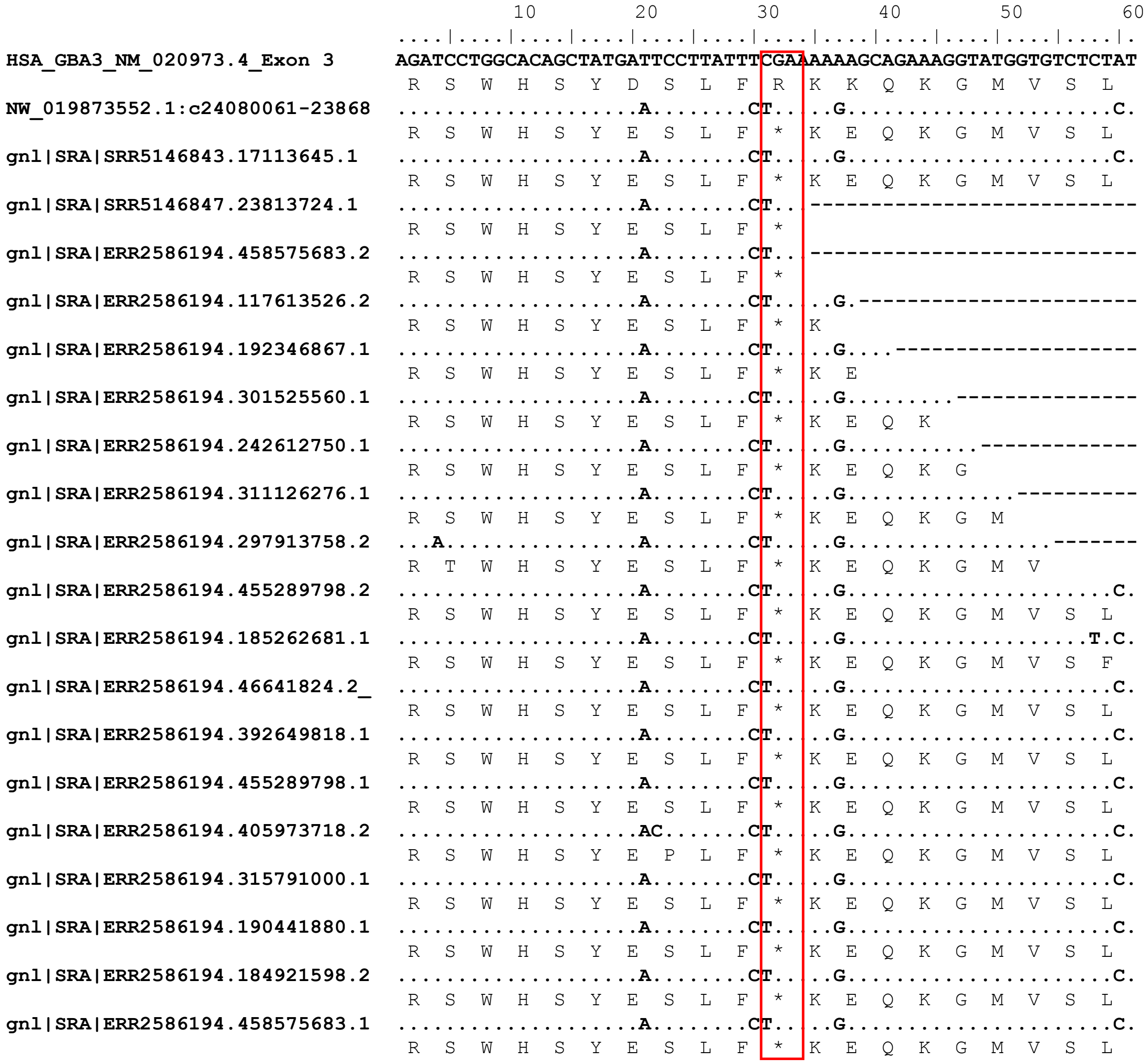

C

Project	Run	Submitted by	DATE	Sample
SRX2447269	SRR5146847	The Genome Center at Washington University School of Medicine in St. Louis	2017-01-05	SAMN06187412 - 892431
SRX2447270	SRR5146843	The Genome Center at Washington University School of Medicine in St. Louis	2017-01-05	SAMN06187412 - 892431
ERX2601917	ERR2586194	CNGB	2018-07-03	SAMEA1067860 - female

Figure S13: **A**-Schematic representation of the identified mutations during gene annotation of GBA3 in *Physeter catodon* using the available genome assembly in NCBI (Scaffold NW_019873552.1). Each group of grey squares represents an exon, number indicated above, yellow indicates loss of canonical splice site (AG-GT), green indicates insertion, blue indicates deletion and read indicates premature stop codon. Numbers in the squares indicate how many nucleotides were inserted or deleted or how many stop codons were identified. **B**-Validation of the identified mutation through multiple sequence alignment of reads obtained from SRA projects. Red box highlights the location of the premature stop codon. **C**-SRA projects consulted.

A

B

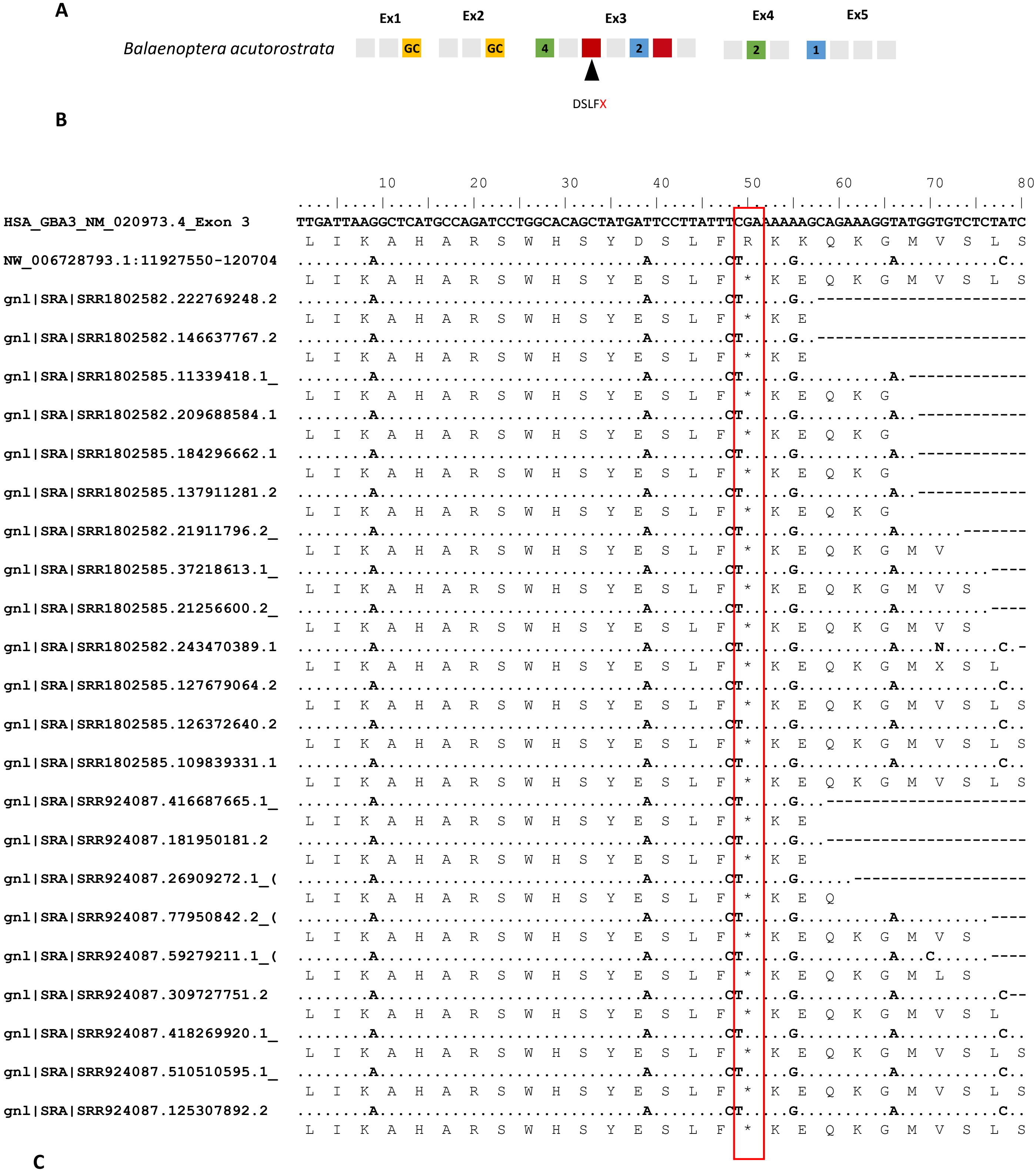

C

Project	Run	Submitted by	DATE	Sample
SRX316738	SRR924087	Korea Institute of Ocean Science and Technology	2013-10-31	SAMN02192644 – MinkeWhale-01
SRX872204	SRR1802582	Seoul National University	2016-02-11	SAMN03339797- MW30
SRX872217	SRR1802585	Seoul National University	2016-02-11	SAMN03339800- MW37

Figure S14: **A**-Schematic representation of the identified mutations during gene annotation of GBA3 in *Balaenoptera acutorostrata* using the available genome assembly in NCBI (Scaffold NW_006728793.1). Each group of grey squares represents an exon, number indicated above, yellow indicates loss of canonical splice site (AG-GT), green indicates insertion, blue indicates deletion and read indicates premature stop codon. Numbers in the squares indicate how many nucleotides were inserted or deleted or how many stop codons were identified. **B**-Validation of the identified mutation through multiple sequence alignment of reads obtained from SRA projects. Red box highlights the location of the premature stop codon. **C**-SRA projects consulted.

A

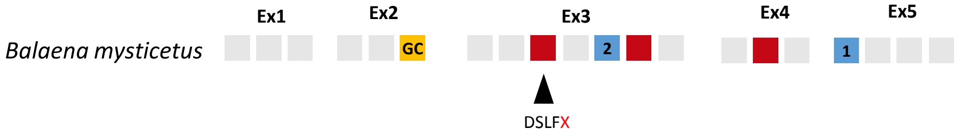

B

```
                                                 10        20        30        40        50        60        70        80
                                        ....|....|....|....|....|....|....|....|....|....|....|....|....|....|....|....|
HSA_GBA3_TV1_NM_020973.4_Exon           TTGATTAAGGCTCATGCCAGATCCTGGCACAGCTATGATTCCTTATTTCGAAAAAGCAGAAAGGTATGGTGTCTCTATC
                                         L  I  K  A  H  A  R  S  W  H  S  Y  D  S  L  F  R  K  K  Q  K  G  M  V  S  L
gnl|BL_ORD_ID|966 scaffold_843          ........A.............................A........CT....G...........A...........C..
                                         L  I  K  A  H  A  R  S  W  H  S  Y  E  S  L  F  *  K  E  Q  K  G  M  V  S  L
gnl|SRA|SRR1685385.305835961.2          ........A.............................A........CT....G..--------------------------
                                         L  I  K  A  H  A  R  S  W  H  S  Y  E  S  L  F  *  K  E
gnl|SRA|SRR1685385.305835963.2          ........A.............................A........CT....G..--------------------------
                                         L  I  K  A  H  A  R  S  W  H  S  Y  E  S  L  F  *  K  E
gnl|SRA|SRR1685385.150701245.2          ........A.............................A........CT....G.......-------------------
                                         L  I  K  A  H  A  R  S  W  H  S  Y  E  S  L  F  *  K  E  Q  K
gnl|SRA|SRR1685385.21925658.2_          ........A.............................A........CT....G...........A...........---
                                         L  I  K  A  H  A  R  S  W  H  S  Y  E  S  L  F  *  K  E  Q  K  G  M  V  S
gnl|SRA|SRR1685385.61661511.2_          ........A.............................A........CT....G...........A...........---
                                         L  I  K  A  H  A  R  S  W  H  S  Y  E  S  L  F  *  K  E  Q  K  G  M  V  S
gnl|SRA|SRR1685385.54663245.2_          ........A.............................A........CT....G...........A...........---
                                         L  I  K  A  H  A  R  S  W  H  S  Y  E  S  L  F  *  K  E  Q  K  G  M  V  S
gnl|SRA|SRR1685385.53557765.1_          ........A.............................A........CT....G...........A...........---
                                         L  I  K  A  H  A  R  S  W  H  S  Y  E  S  L  F  *  K  E  Q  K  G  M  V  S
gnl|SRA|SRR1685385.87139334.2_          ........A.............................A........CT....G...........A...........---
                                         L  I  K  A  H  A  R  S  W  H  S  Y  E  S  L  F  *  K  E  Q  K  G  M  V  S
gnl|SRA|SRR1685385.241193186.1          ........A.............................A........CT....G...........A...........C..
                                         L  I  K  A  H  A  R  S  W  H  S  Y  E  S  L  F  *  K  E  Q  K  G  M  V  S  L
gnl|SRA|SRR1685386.310772961.2          ........A.............................A........CT....G...........A...........C..
                                         L  I  K  A  H  A  R  S  W  H  S  Y  E  S  L  F  *  K  E  Q  K  G  M  V  S  L
gnl|SRA|SRR1685386.304081513.1          ........A.............................A........CT....G...........A...........C..
                                         L  I  K  A  H  A  R  S  W  H  S  Y  E  S  L  F  *  K  E  Q  K  G  M  V  S  L
gnl|SRA|SRR1685386.290098590.2          ........A.............................A........CT....G...........A...........C..
                                         L  I  K  A  H  A  R  S  W  H  S  Y  E  S  L  F  *  K  E  Q  K  G  M  V  S  L
gnl|SRA|SRR1685386.280454827.1          ........A.............................A........CT....G...........A...........C..
                                         L  I  K  A  H  A  R  S  W  H  S  Y  E  S  L  F  *  K  E  Q  K  G  M  V  S  L
```

C

Project	Run	Submitted by	DATE	Sample
SRX790318	SRR1685386	University of Liverpool	2015-01-06	SAMN03225705 –ID325 female
SRX790317	SRR1685385	University of Liverpool	2015-01-06	SAMN03225705 –ID325 female

Figure S15: **A**-Schematic representation of the identified mutations during gene annotation of GBA3 in *Balaena mysticetus* using the available genome assembly in NCBI (Scaffold 843). Each group of grey squares represents an exon, number indicated above, yellow indicates loss of canonical splice site (AG-GT), green indicates insertion, blue indicates deletion and read indicates premature stop codon. Numbers in the squares indicate how many nucleotides were inserted or deleted or how many stop codons were identified. **B**-Validation of the identified mutation through multiple sequence alignment of reads obtained from SRA projects. Red box highlights the location of the premature stop codon. **C**-SRA projects consulted.

SUPPLEMENTARY MATERIAL- 5

Supplementary Material 5 - Validation of the identified mutations in Carnivora

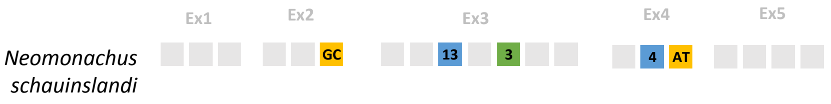

Only one DNA project available in NCBI and which was not accessible for blast searches (**ERX2808478**)
Blast on RNA project (**SRX2771656)** retrieves approximately 100 reads covering exon 5 and 3 however this reads do not cover the 13 nucleotide deletion region (Figure 1 and S16). Additionally the low number of reads collected and the absence of mature RNA reads (spanning between 2 exons) supports the predicted pseudogene status of *GBA3* in this specie.

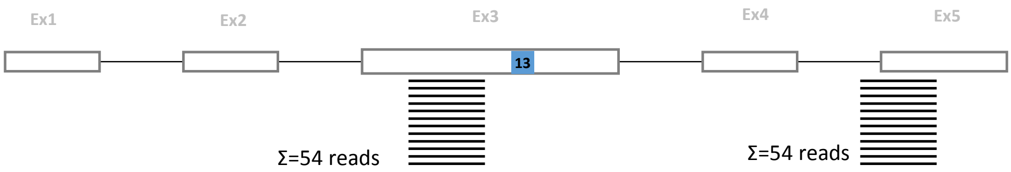

Figure S16: Mapping of the collected RNA reads on *GBA3* gene

A

B

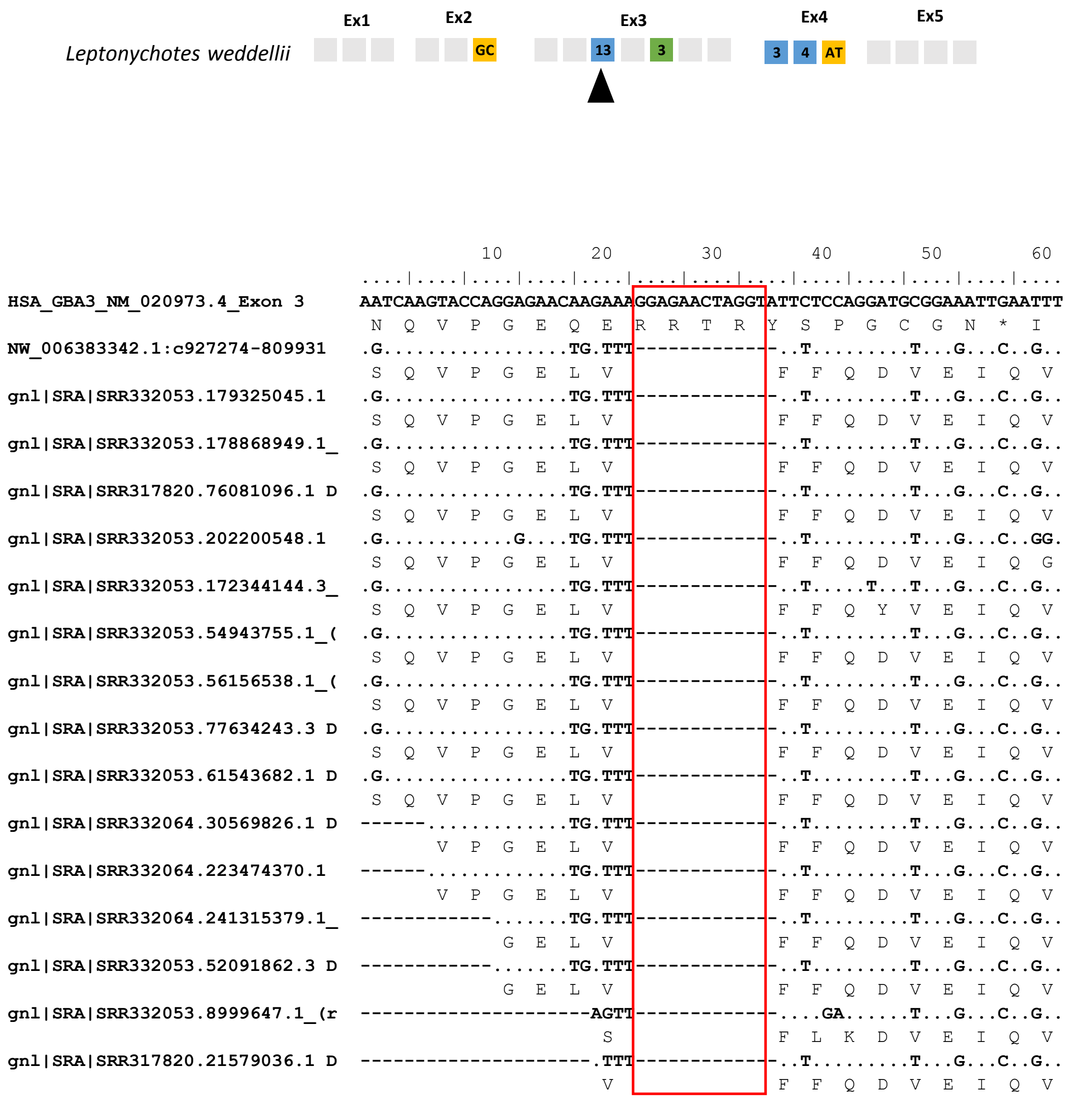

C

Project	Run	Submitted by	DATE	Sample
SRX085502	SRR317820	Broad Institute (BI)	2011-07-29	SAMN00672463 WS11-02 female
SRX091967	SRR332053	Broad Institute (BI)	2011-08-22	SAMN00672463 WS11-02 female
SRX091975	SRR332064	Broad Institute (BI)	2011-08-22	SAMN00672463 WS11-02 female

Figure S17: **A**-Schematic representation of the identified mutations during gene annotation of GBA3 in *Leptonychotes weddellii* using the available genome assembly in NCBI (Scaffold NW_006383342.1). Each group of grey squares represents an exon, number indicated above, yellow indicates loss of canonical splice site (AG-GT), green indicates insertion, blue indicates deletion and read indicates premature stop codon. Numbers in the squares indicate how many nucleotides were inserted or deleted or how many stop codons were identified. **B**-Validation of the identified mutation through multiple sequence alignment of reads obtained from SRA projects. Red box highlights the location of the 13 nucleotide deletion. **C**-SRA projects consulted.

A

B

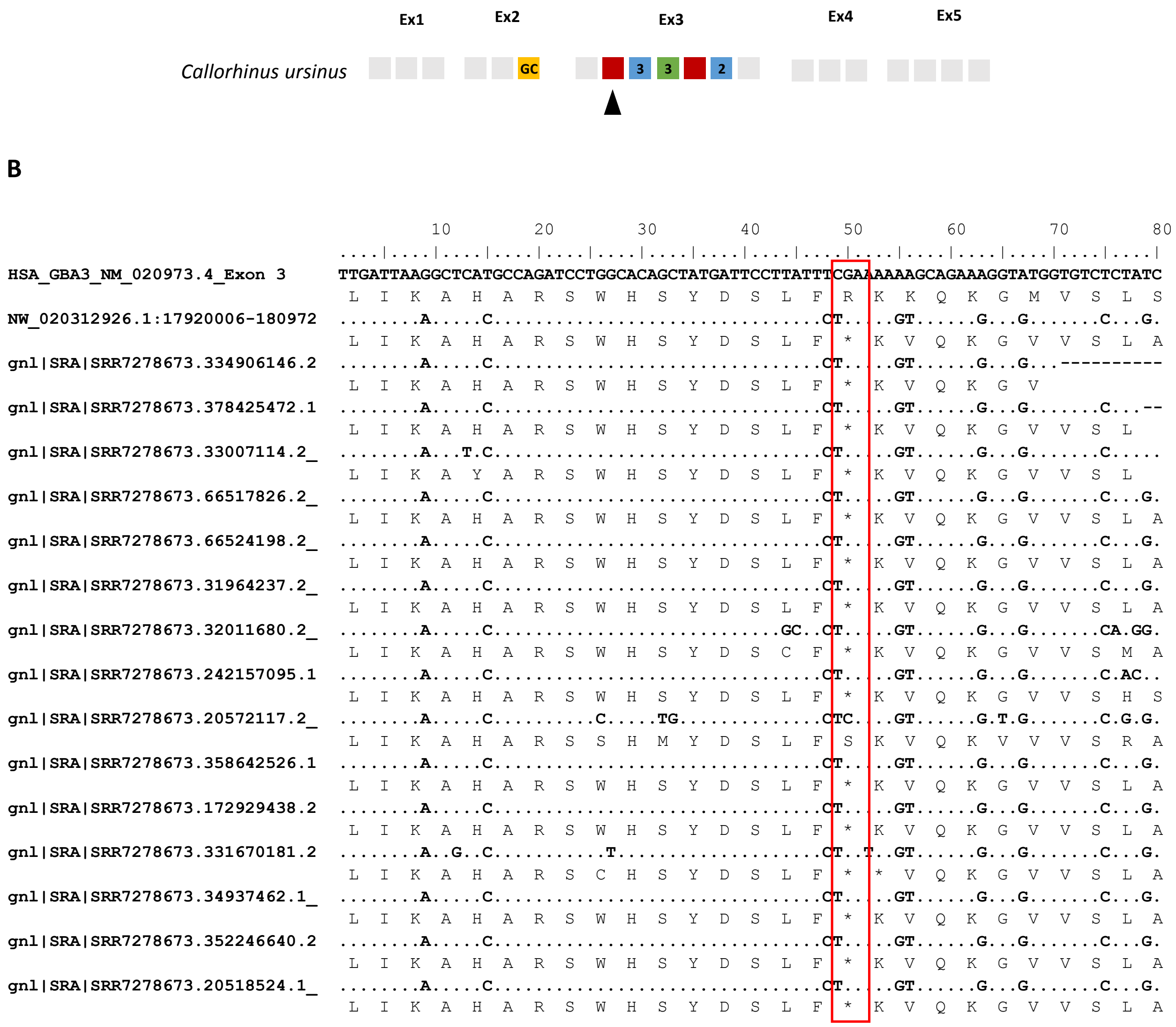

C

Project	Run	Submitted by	DATE	Sample
SRX4182256	SRR7278673	BC Cancer Agency Michael Smith Genome Sciences Centre	2018-06-21	SAMN09379881 SJ78 female

Figure S18: **A**-Schematic representation of the identified mutations during gene annotation of GBA3 in *Callorhinus ursinus* using the available genome assembly in NCBI (Scaffold NW_020312926.1). Each group of grey squares represents an exon, number indicated above, yellow indicates loss of canonical splice site (AG-GT), green indicates insertion, blue indicates deletion and read indicates premature stop codon. Numbers in the squares indicate how many nucleotides were inserted or deleted or how many stop codons were identified. **B**-Validation of the identified mutation through multiple sequence alignment of reads obtained from SRA projects. Red box highlights the location of the premature stop codon. **C**-SRA projects consulted.

A

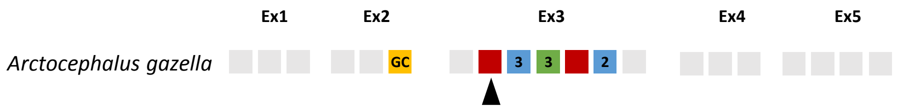

B

```
                                         10        20        30        40        50        60        70
                                 ....|....|....|....|....|....|....|....|....|....|....|....|....|....|
HSA_GBA3_NM_020973.4_Eeon 3      AGATCCTGGCACAGCTATGATTCCTTATTTCGAAAAAAGCAGAAAGGTATGGTGTCTCTATCACTTTTTG
                                   R  S  W  H  S  Y  D  S  L  F  R  K  K  Q  K  G  M  V  S  L  S  L  F
UIRR01000237.1:1357959-1559759   AGATCCTGGCACAGCTATGATTCCTTATTCTGAAAAGTGCAGAAGGGTGTGGTGTCCCTAGCAATTTTTG
                                   R  S  W  H  S  Y  D  S  L  F  *  K  V  Q  K  G  V  V  S  L  A  I  F
gnl|SRA|SRR2658583.85020411.1_   AGATCCTGGCACAGCTATGATTCCTTATTCTGAAAAGTGCAGAAGGG
                                   R  S  W  H  S  Y  D  S  L  F  *  K  V  Q  K
gnl|SRA|SRR2658583.155667376.1   AGATCCTGGCACAGCTATGATTCCTTATTCTGAAAAGTGCAGAAGGGT
                                   R  S  W  H  S  Y  D  S  L  F  *  K  V  Q  K  G
gnl|SRA|SRR2658584.16079018.2    AGATCCTGGCACAGCTATGATTCCTTATTCTGAAAAGTGCAGAAGGGTGTGGTGTCCCTAGCA
                                   R  S  W  H  S  Y  D  S  L  F  *  K  V  Q  K  G  V  V  S  L  A
gnl|SRA|SRR2658583.24179347.2_   AGATCCTGGCACAGCTATGATTCCTTATTCTGAAAAGTGCAGAAGGGTGTGGTGTCCCTAGCAAT
                                   R  S  W  H  S  Y  D  S  L  F  *  K  V  Q  K  G  V  V  S  L  A
gnl|SRA|SRR2658579.6211843.2_(   AGATCCTGGCACAGCTATGATTCCTTATTCTGAAAAGTGCAGAAGGGTGTG
                                   R  S  W  H  S  Y  D  S  L  F  *  K  V  Q  K  G  V
gnl|SRA|SRR2658579.1282270.2_(   AGATCCTGGCACAGCTATGATTCCTTATTCTGAAAAGTGCAGAAGGGTGTG
                                   R  S  W  H  S  Y  D  S  L  F  *  K  V  Q  K  G  V
gnl|SRA|SRR2658579.6211843.2_(   AGATCCTGGCACAGCTATGATTCCTTATTCTGAAAAGTGCAGAAGGGTGTG
                                   R  S  W  H  S  Y  D  S  L  F  *  K  V  Q  K  G  V
gnl|SRA|SRR2658579.1282270.2_(   AGATCCTGGCACAGCTATGATTCCTTATTCTGAAAAGTGCAGAAGGGTGTG
                                   R  S  W  H  S  Y  D  S  L  F  *  K  V  Q  K  G  V
gnl|SRA|SRR2658585.49667850.2_   AGATCCTGGCACAGCTATGATTCCTTATTCTGAAAAGTGCAGAAGGGTGTGGTGTCCCTAGCAATTTTTG
                                   R  S  W  H  S  Y  D  S  L  F  *  K  V  Q  K  G  V  V  S  L  A  I  F
gnl|SRA|SRR2658583.96667248.1_   AGATCCTGGCACAGCTATGATTCCTTATTCTGAAAAGTGCAGAAGGGTGTGGTGTCCCTAGCAATTTTTG
                                   R  S  W  H  S  Y  D  S  L  F  *  K  V  Q  K  G  V  V  S  L  A  I  F
gnl|SRA|SRR2658581.29500941.1    AGATCCTGGCACAGCTATGATTCCTTATTCTGAAAAGTGCAGAAGGGTGT
                                   R  S  W  H  S  Y  D  S  L  F  *  K  V  Q  K  G
gnl|SRA|SRR2658580.6904844.1     AGATCCTGGCACAGCTATGATTCCTTATTCTGAAAAGTGCAGAAGGGTGT
                                   R  S  W  H  S  Y  D  S  L  F  *  K  V  Q  K  G
gnl|SRA|SRR2658584.76763955.2_   AGATCGTGGCACAGCTATGATTCCTTATTCTGAAAAGTGCAGAAGGGTGTGGTGTCNCTAGCAATTTTTG
                                   R  S  W  H  S  Y  D  S  L  F  *  K  V  Q  K  G  V  V  X  L  A  I  F
gnl|SRA|SRR2658581.9335601.2_(   AGATCCTGGCACAGCTATGATTCCTTATTCTGAAAAGTGCAGAAGGGTGTGGTGTCCCTAGCAATTTTTG
                                   R  S  W  H  S  Y  D  S  L  F  *  K  V  Q  K  G  V  V  S  L  A  I  F
gnl|SRA|SRR2658583.44426819.2_   AGATCCTGGCACAGCTATGATTCCTTATTCTGAAAAGTGCAGAAGGGTGTGGTGTCCCTAGCAATTTTTG
                                   R  S  W  H  S  Y  D  S  L  F  *  K  V  Q  K  G  V  V  S  L  A  I  F
gnl|SRA|SRR2658583.74042440.2_   AGATCCTGGCACAGCTATGATTCCTTATTCTGAAAAGTGCAGAAGGGTGTGGTGTCCCTAGCAATTTTTG
                                   R  S  W  H  S  Y  D  S  L  F  *  K  V  Q  K  G  V  V  S  L  A  I  F
gnl|SRA|SRR2658581.15796689.1    AGATCCTGGCACAGCTATGATTCCTTATTCTGAAAAGTGCAGAAGGGTGTGGTGTCCCTAGCAATTTTTG
                                   R  S  W  H  S  Y  D  S  L  F  *  K  V  Q  K  G  V  V  S  L  A  I  F
gnl|SRA|SRR2658583.62553491.2    AGATCCTGGCACAGCTATGATTCCTTATTATGAAAAGTGCAGAAGGGTGTGGTGTCCCTAGCAAATTTTT
                                   R  S  W  H  S  Y  D  S  L  L  *  K  V  Q  K  G  V  V  S  L  A  N  F
gnl|SRA|SRR2658583.1338996.1     AGATCCTGGCACAGCTATGATTCCTTATTCTGAAAAGTGCAGAAGGGTGTGGTGTCCCTAGCAATTTTTG
                                   R  S  W  H  S  Y  D  S  L  F  *  K  V  Q  K  G  V  V  S  L  A  I  F
```

C

Project	Run	Submitted by	DATE	Sample
SRX1338504	SRR2658583	UPPSALA UNIVERSITY	2015-11-23	SAMN04159679 female
SRX1338505	SRR2658584	UPPSALA UNIVERSITY	2015-11-23	SAMN04159679 female
SRX1338500	SRR2658579	UPPSALA UNIVERSITY	2015-11-23	SAMN04159679 female
SRX1338506	SRR2658585	UPPSALA UNIVERSITY	2015-11-23	SAMN04159679 female
SRX1338502	SRR2658581	UPPSALA UNIVERSITY	2015-11-23	SAMN04159679 female
SRX1338501	SRR2658580	UPPSALA UNIVERSITY	2015-11-23	SAMN04159679 female

Figure S19: **A**-Schematic representation of the identified mutations during gene annotation of GBA3 in *Arctocephalus gazella* using the available genome assembly in NCBI (Scaffold UIRR01000237.1). Each group of grey squares represents an exon, number indicated above, yellow indicates loss of canonical splice site (AG-GT), green indicates insertion, blue indicates deletion and read indicates premature stop codon. Numbers in the squares indicate how many nucleotides were inserted or deleted or how many stop codons were identified. **B**-Validation of the identified mutation through multiple sequence alignment of reads obtained from SRA projects. Red box highlights the location of the premature stop codon. **C**-SRA projects consulted.

A

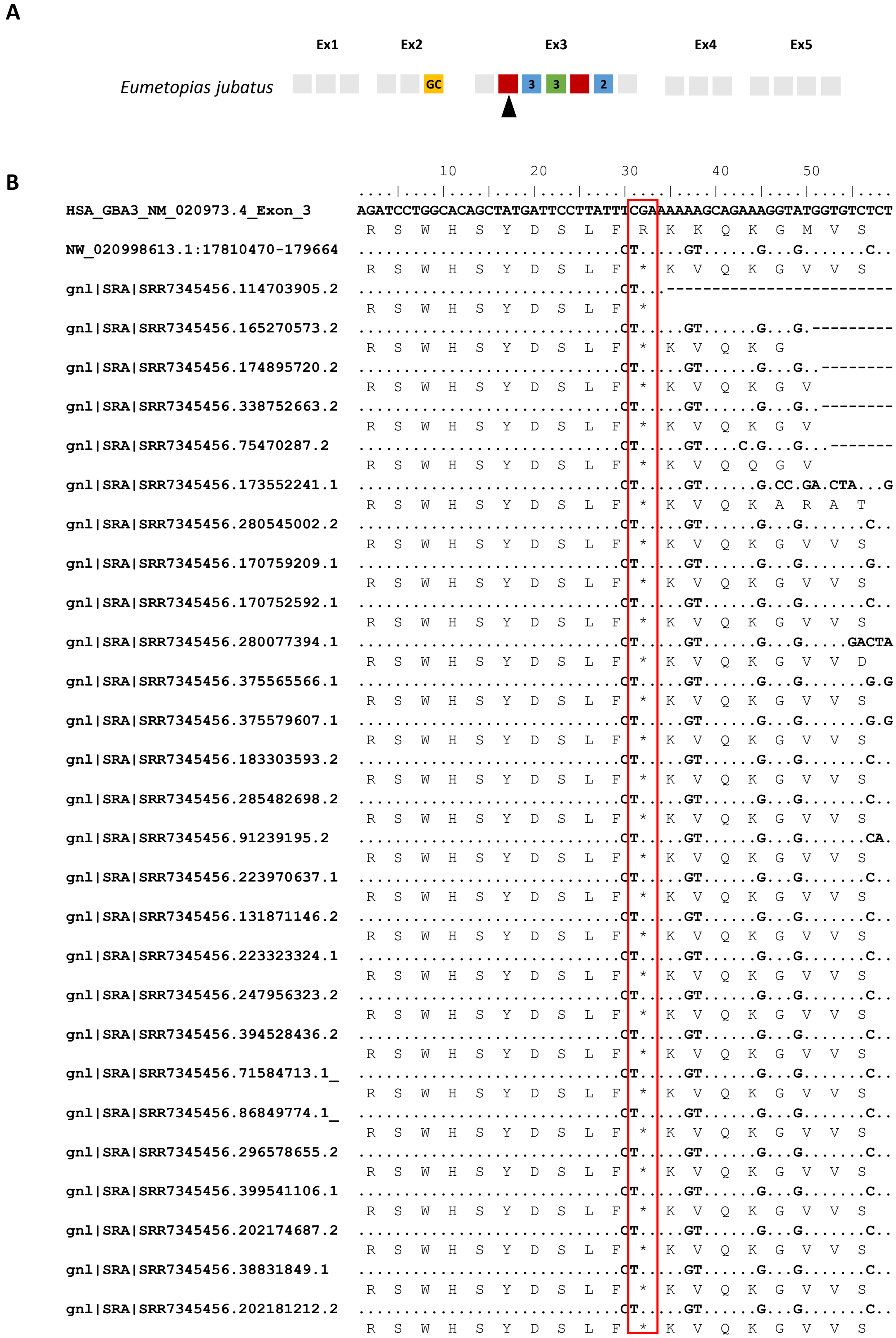

C

Project	Run	Submitted by	DATE	Sample
SRX4218881	SRR7345456	BC Cancer Agency Michael Smith Genome Sciences Centre	2018-06-21	SAMN09402722 SJ_48 female

Figure S20: **A**-Schematic representation of the identified mutations during gene annotation of GBA3 in *Eumetopias jubatus* using the available genome assembly in NCBI (Scaffold NW_020998613.1). Each group of grey squares represents an exon, number indicated above, yellow indicates loss of canonical splice site (AG-GT), green indicates insertion, blue indicates deletion and read indicates premature stop codon. Numbers in the squares indicate how many nucleotides were inserted or deleted or how many stop codons were identified. **B**-Validation of the identified mutation through multiple sequence alignment of reads obtained from SRA projects. Red box highlights the location of the premature stop codon. **C**-SRA projects consulted.

Zalophus californianus presented 2 genome assemblies, gene annotation was performed on the assembly ZalCal2.2 (2018/12/07) GCA_900631625.1. Scaffold NW_020874464.1
Blastn search was performed on the most recent unannotated second assembly ZalCal_v1_BIUU (2019/01/05) GCA_004024565.1 to confirm the identified stop codon in exon 3 scaffold PISZ01005978.1.
Annotation revealed that both assemblies presented the exact same mutations in exon 3

A single SRA attributed to this species SRX007150, corresponds in fact to nasal swabs of sea lion used to investigate calicivirus. Nevertheless this project was also searched retrieving no hits

A

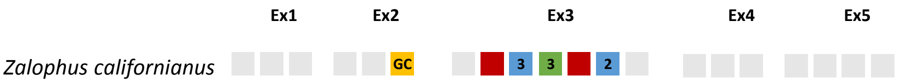

B

```
                                      10        20        30        40        50        60        70        80        90
                              ....|....|....|....|....|....|....|....|....|....|....|....|....|....|....|....|....|....|
HSA_GBA3_TV1_NM_020973.4-Exon3 GAATTGATTATTACAACAAGATCATCGATGATTTGTTAAAAAATGGGGTTACTCCCATTGTGACCCTCTACCACTTTGATTTGCCTCAGA
                                 I  D  Y  Y  N  K  I  I  D  D  L  L  K  N  G  V  T  P  I  V  T  L  Y  H  F  D  L  P  Q
PISZ01005978.1                 .......C.................T.............CT....C......G..T..A................A.............G
                                 I  D  Y  Y  N  K  I  I  D  D  L  L  T  N  A  V  T  P  I  V  T  L  Y  H  Y  D  L  P  Q
NW_020874464.1                 .......C.................T.............CT....C......G..T..A................A.............G
                                 I  D  Y  Y  N  K  I  I  D  D  L  L  T  N  A  V  T  P  I  V  T  L  Y  H  Y  D  L  P  Q

                                     100       110       120       130       140       150       160       170       180
                              ....|....|....|....|....|....|....|....|....|....|....|....|....|....|....|....|....|....|
HSA_GBA3_TV1_NM_020973.4-Exon3 CTTTAGAAGACCAAGGAGGTTGGTTGTCAGAGGCAATCATTGAATCCTTTGACAAATATGCTCAGTTTTGCTTCAGTACCTTTGGGGATC
                               T  L  E  D  Q  G  G  W  L  S  E  A  I  I  E  S  F  D  K  Y  A  Q  F  C  F  S  T  F  G  D
PISZ01005978.1                 .C.........A........................................T........C.G...............T.....A....
                               A  L  E  D  K  G  G  W  L  S  E  A  I  I  E  S  F  D  K  Y  A  R  F  C  F  S  T  F  G  D
NW_020874464.1                 .C.........A........................................T........C.G...............T.....A....
                               A  L  E  D  K  G  G  W  L  S  E  A  I  I  E  S  F  D  K  Y  A  R  F  C  F  S  T  F  G  D

                                     190       200       210       220       230       240       250       260       270
                              ....|....|....|....|....|....|....|....|....|....|....|....|....|....|....|....|....|....|
HSA_GBA3_TV1_NM_020973.4-Exon3 GTGTCAAGCAGTGGATCACCATAAATGAAGCTAATGTTCTTTCTGTGATGTCATATGACTTAGGTATGTTTCCTCCGGGTATCCCTCACT
                               R  V  K  Q  W  I  T  I  N  E  A  N  V  L  S  V  M  S  Y  D  L  G  M  F  P  P  G  I  P  H
PISZ01005978.1                 .A...C..TG....G.............GC........T..G.CC..C..G.......T..T..A..A.A......T...G.A.......
                               R  V  Q  W  W  V  T  I  N  E  P  N  V  F  A  L  L  A  Y  D  F  G  I  Y  P  P  G  V  P  H
NW_020874464.1                 .A...C..TG....G.............GC........T..G.CC..C..G.......T..T..A..A.A......T...G.A.......
                               R  V  Q  W  W  V  T  I  N  E  P  N  V  F  A  L  L  A  Y  D  F  G  I  Y  P  P  G  V  P  H

                                     280       290       300       310       320       330       340       350       360
                              ....|....|....|....|....|....|....|....|....|....|....|....|....|....|....|....|....|....|
HSA_GBA3_TV1_NM_020973.4-Exon3 TTGGGACTGGAGGTTATCAGGCAGCTCATAATTTGATTAAGGCTCATGCCAGATCCTGGCACAGCTATGATTCCTTATTTCGAAAAAAGC
                               F  G  T  G  G  Y  Q  A  A  H  N  L  I  K  A  H  A  R  S  W  H  S  Y  D  S  L  F  R  K  K
PISZ01005978.1                 ....A...................................A.....C................A...............CT.....GT..
                               F  G  T  G  G  Y  Q  A  A  H  N  L  I  K  A  H  A  R  S  W  H  N  Y  D  S  L  F  *  K  V
NW_020874464.1                 ....A...................................A.....C................A...............CT.....GT..
                               F  G  T  G  G  Y  Q  A  A  H  N  L  I  K  A  H  A  R  S  W  H  N  Y  D  S  L  F  *  K  V

                                     370       380       390       400       410       420       430       440       450
                              ....|....|....|....|....|....|....|....|....|....|....|....|....|....|....|....|....|....|
HSA_GBA3_TV1_NM_020973.4-Exon3 AGAAAGGTATGGTGTCTCTATCACTTTTTGCGGTCTGGTTGGAACCAGCAGATCCCAACTCAGTGTCTGACCAGGAAGCTGCTAAAAGAG
                               Q  K  G  M  V  S  L  S  L  F  A  V  W  L  E  P  A  D  P  N  S  V  S  D  Q  E  A  A  K  R
PISZ01005978.1                 ....G...G.......C...G..A.......TAC....G.......G...........................................
                               Q  K  G  V  V  S  L  A  I  F  A  T  W  V  E  P  A  D  P  N  S  V  S  D  Q  E  A  A  K  R
NW_020874464.1                 ....G...G.......C...G..A.......TAC....G.......G...........................................
                               Q  K  G  V  V  S  L  A  I  F  A  T  W  V  E  P  A  D  P  N  S  V  S  D  Q  E  A  A  K  R

                                     460       470       480       490       500       510       520       530       540
                              ....|....|....|....|....|....|....|....|....|....|....|....|....|....|....|....|....|....|
HSA_GBA3_TV1_NM_020973.4-Exon3 CCATCACTTTCCATCTGGATTTATTTGCTAAACCCATATTCATCGATGGTGATTATCCTGAAGTTGTCAAGTCTCAGATTGCCTCCATGA
                               A  I  T  F  H  L  D  L  F  A  K  P  I  F  I  D  G  D  Y  P  E  V  V  K  S  Q  I  A  S  M
PISZ01005978.1                 ..C.TG.....TGCT.......C.................T.CT.T............G..............C................
                               A  L  A  F  C  L  D  F  F  A  K  P  I  F  T  V  G  D  Y  P  E  V  V  K  S  Q  I  A  F  M
NW_020874464.1                 ..C.TG.....TGCT.......C.................T.CT.T............G..............C..........T.....
                               A  L  A  F  C  L  D  F  F  A  K  P  I  F  T  V  G  D  Y  P  E  V  V  K  S  Q  I  A  F  M
```

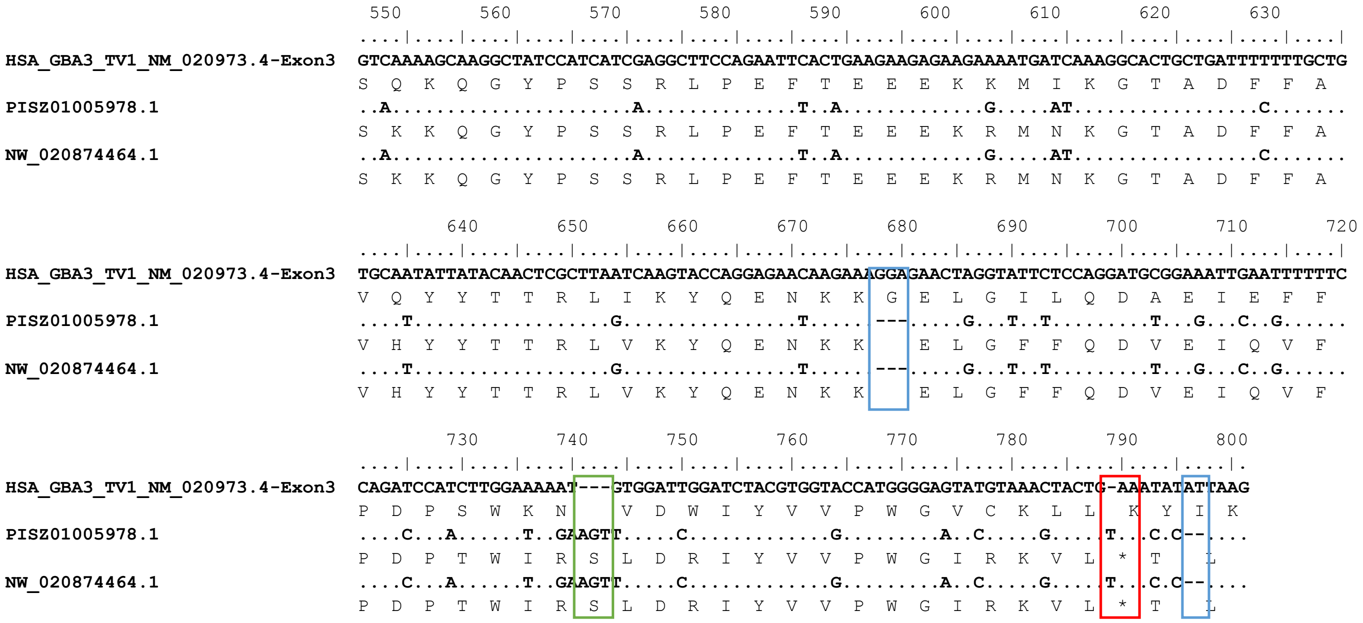

Figure S21: **A**-Schematic representation of the identified mutations during gene annotation of GBA3 in *Eumetopias jubatus* using the available genome assembly in NCBI (Scaffold NW_020998613.1). Each group of grey squares represents an exon, number indicated above, yellow indicates loss of canonical splice site (AG-GT), green indicates insertion, blue indicates deletion and read indicates premature stop codon. Numbers in the squares indicate how many nucleotides were inserted or deleted or how many stop codons were identified. **B**-Validation of the identified mutation through multiple sequence alignment of two distinct genomic assemblies ZalCal2.2 (2018/12/07) and GCA_900631625.1. Scaffold NW_020874464.1. Red boxes highlight premature stop codon, blue boxes highlight deletions and Green boxes highlight insertions.

To further validate stop codon mutation found in exon 3 we next searched the unannotated genome of a related *Zalophus wollebaeki,* confirming this mutation in at least 4 independent individuals.

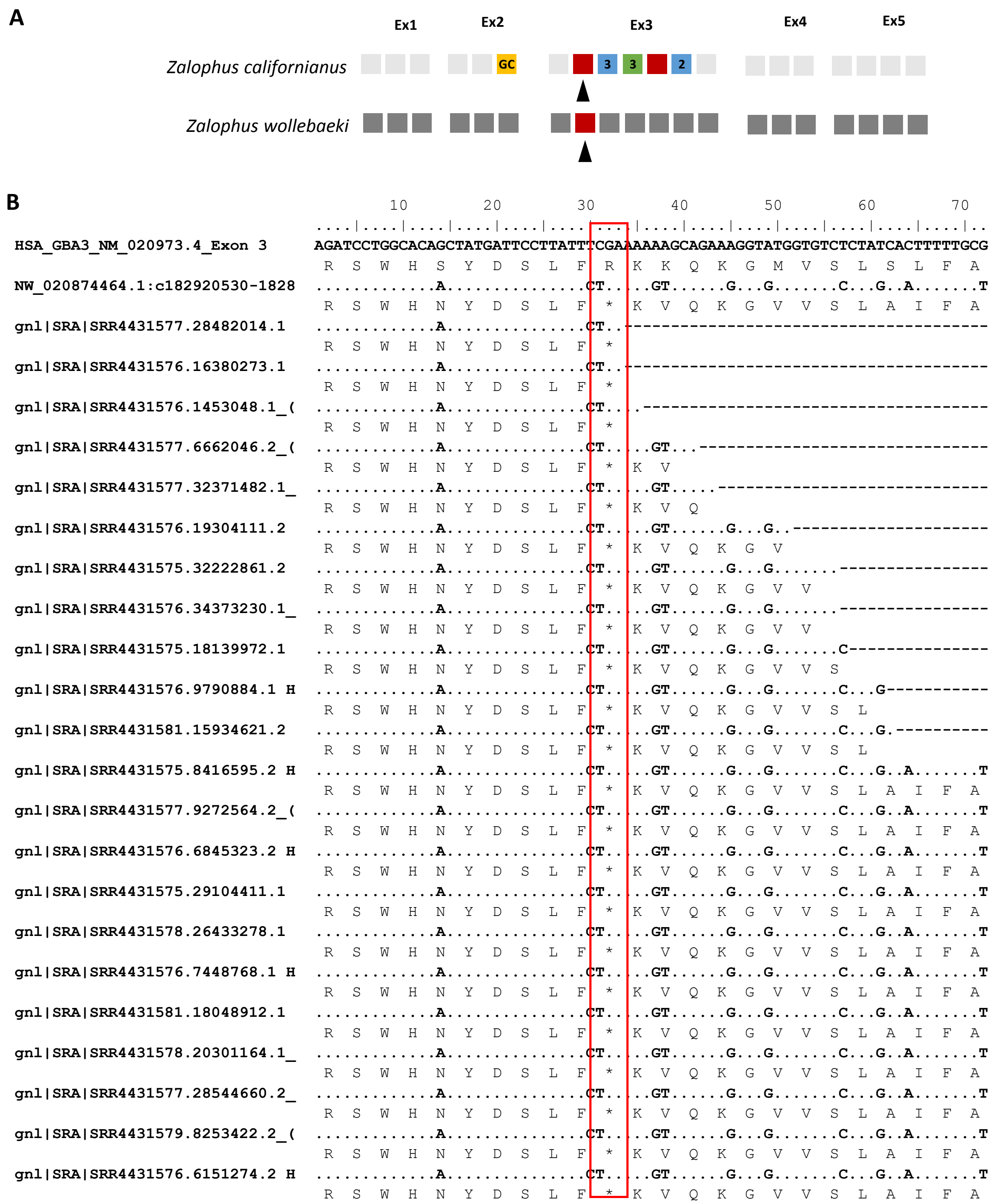

C

Project	Run	Submitted by	DATE	Sample
SRX2251477	SRR4431575	Uppsala University	2017-08-31	SAMN05905682 MO39 female
SRX2251478	SRR4431576	Uppsala University	2017-08-31	SAMN05905681 Mo17 female
SRX2251479	SRR4431577	Uppsala University	2017-08-31	SAMN05905681 Mo17 female
SRX2251480	SRR4431578	Uppsala University	2017-08-31	SAMN05905680 PC28 female
SRX2251481	SRR4431579	Uppsala University	2017-08-31	SAMN05905680 PC28 female
SRX2251483	SRR4431581	Uppsala University	2017-08-31	SAMN05905684 EG05 female

Figure S22: **A**-Schematic representation of the identified mutations during gene annotation of GBA3 in *Zalophus wollebaeki* using the available SRA projects. Each group of grey squares represents an exon, number indicated above, yellow indicates loss of canonical splice site (AG-GT), green indicates insertion, blue indicates deletion and read indicates premature stop codon. Numbers in the squares indicate how many nucleotides were inserted or deleted or how many stop codons were identified. **B**-Validation of the identified mutation through multiple sequence alignment of reads obtained from SRA projects. Red box highlights the location of the premature stop codon. **C-**SRA projects consulted.

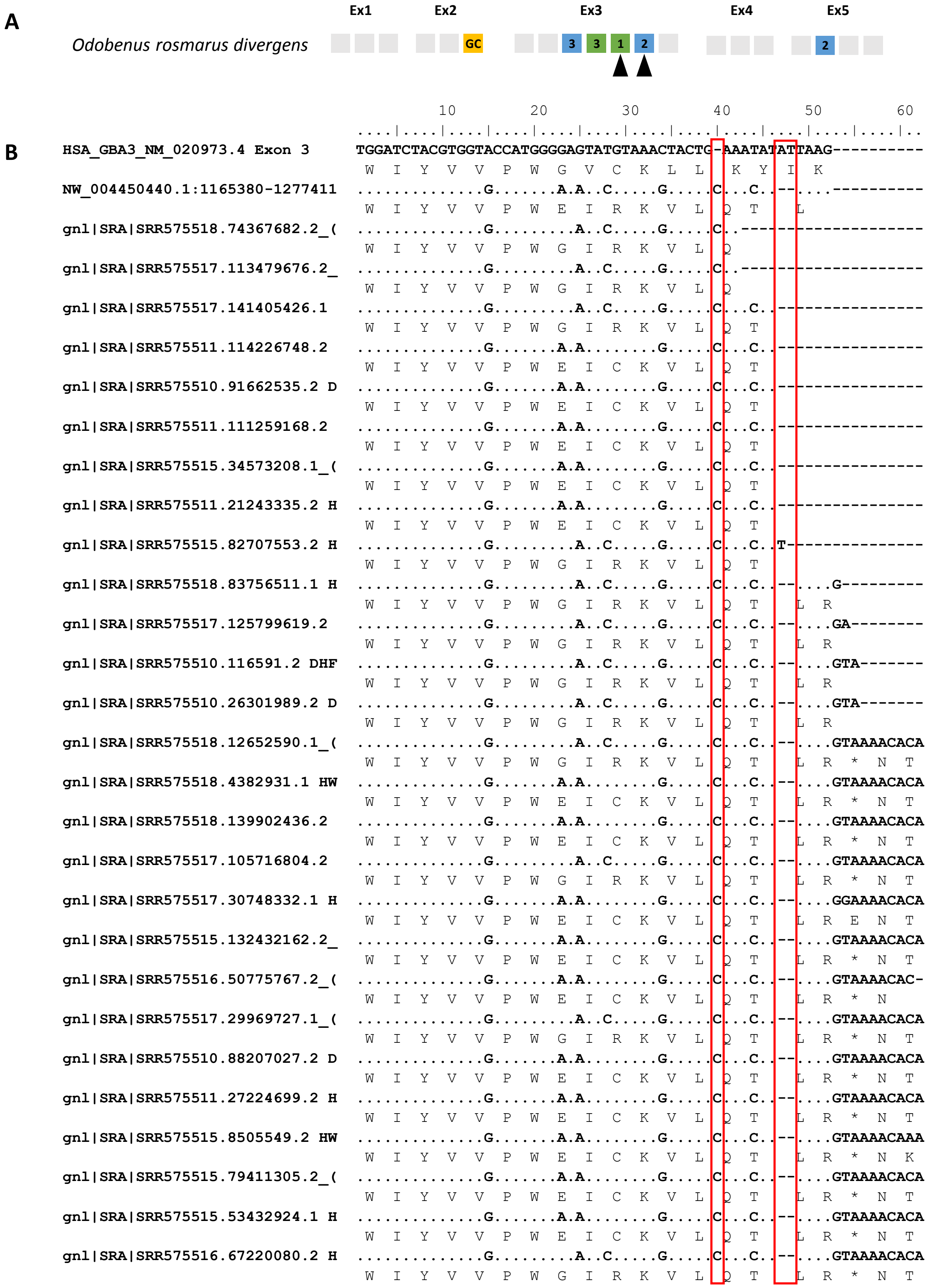

C

Project	Run	Submitted by	DATE	Sample
SRX189120	SRR575510	Baylor College of Medicine	2012-09-21	SAMN01180787 - AFwalrus1
SRX189120	SRR575511	Baylor College of Medicine	2012-09-21	SAMN01180787 - AFwalrus1
SRX189122	SRR575515	Baylor College of Medicine	2012-09-21	SAMN01180787 - AFwalrus1
SRX189122	SRR575516	Baylor College of Medicine	2012-09-21	SAMN01180787 - AFwalrus1
SRX189122	SRR575517	Baylor College of Medicine	2012-09-21	SAMN01180787 - AFwalrus1
SRX189122	SRR575518	Baylor College of Medicine	2012-09-21	SAMN01180787 - AFwalrus1

Figure S23: **A**-Schematic representation of the identified mutations during gene annotation of GBA3 in *Odobenus rosmarus divergens* using the available genome assembly in NCBI (Scaffold NW_004450440.1). Each group of grey squares represents an exon, number indicated above, yellow indicates loss of canonical splice site (AG-GT), green indicates insertion, blue indicates deletion and read indicates premature stop codon. Numbers in the squares indicate how many nucleotides were inserted or deleted or how many stop codons were identified. **B**-Validation of the identified mutation through multiple sequence alignment of reads obtained from SRA projects. Red box highlights the location of the insertions and deletions. **C**-SRA projects consulted.

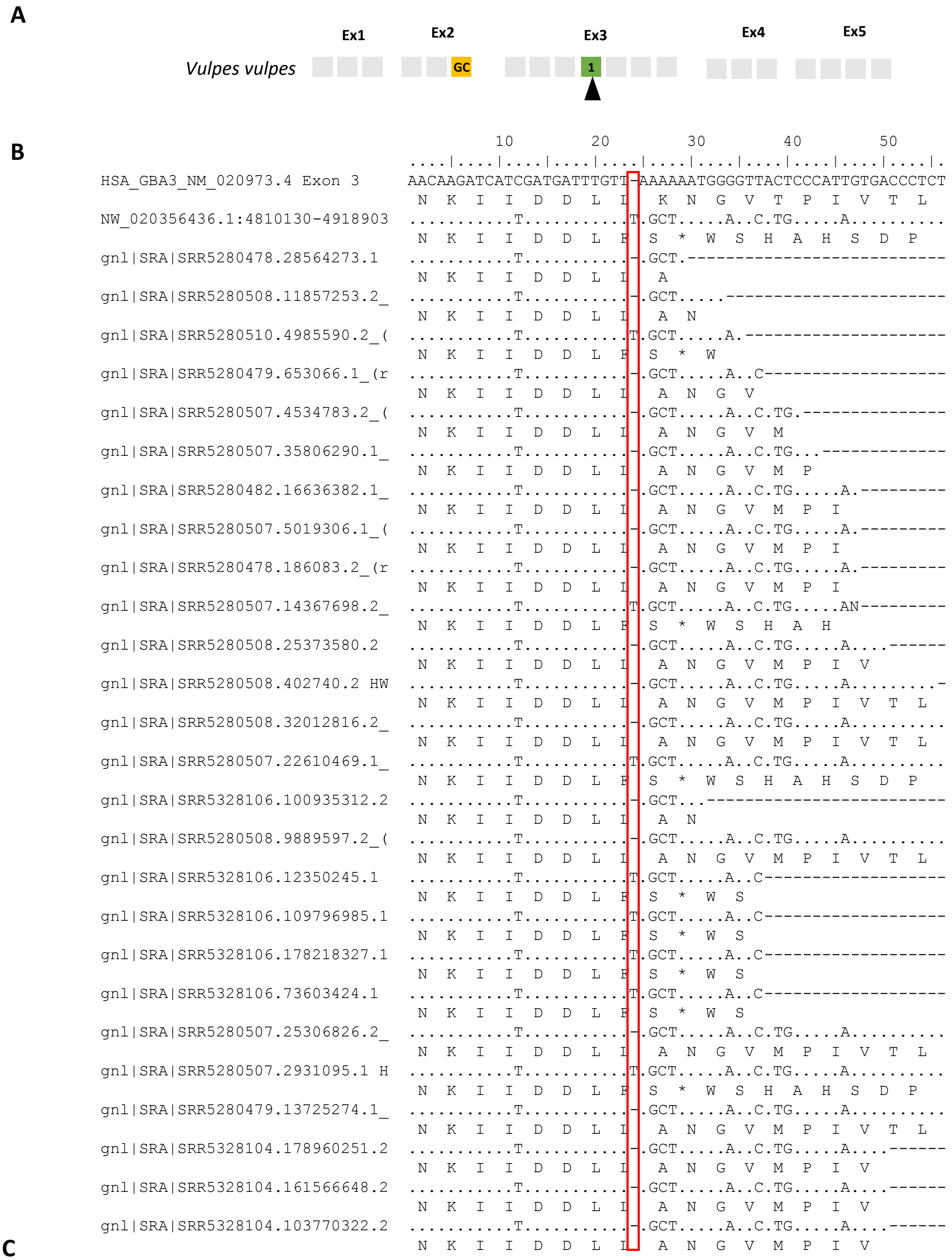

C

Project	Run	Submitted by	DATE	Sample	Genotype
SRX2627137	SRR5328106	University of Illinois at Urbana-Champaign	2018-02-26	SAMN06553695 Reef male	Heterozygous
SRX2627135	SRR5328104	University of Illinois at Urbana-Champaign	2018-02-26	SAMN06553695 Reef male	Heterozygous
SRX2584171	SRR5280479	University of Illinois at Urbana-Champaign	2018-02-26	SAMN06434883 CONV07 female	coding (C)
SRX2584170	SRR5280478	University of Illinois at Urbana-Champaign	2018-02-26	SAMN06434884 CONV08 female	coding (C)
SRX2584202	SRR5280510	University of Illinois at Urbana-Champaign	2018-02-26	SAMN06434857 TAME01 female	Pseudo 1 read (T)
SRX2584200	SRR5280508	University of Illinois at Urbana-Champaign	2018-02-26	SAMN06434859 TAME03 female	Coding (T)
SRX2584199	SRR5280507	University of Illinois at Urbana-Champaign	2018-02-26	SAMN06434860 TAME04 male	Heterozygous
SRX2584174	SRR5280482	University of Illinois at Urbana-Champaign	2018-02-26	SAMN06434880 CON04 male	Coding 1 read (C)

Figure S24: **A**-Schematic representation of the identified mutations during gene annotation of GBA3 in *Vulpes vulpes* using the available genome assembly in NCBI (Scaffold NW_020356436.1). Each group of grey squares represents an exon, number indicated above, yellow indicates loss of canonical splice site (AG-GT), green indicates insertion, blue indicates deletion and read indicates premature stop codon. Numbers in the squares indicate how many nucleotides were inserted or deleted or how many stop codons were identified. **B**-Validation of the identified mutation through multiple sequence alignment of reads obtained from SRA projects. Red box highlights the location of the 1bp deletion. **C**-SRA projects consulted.

A

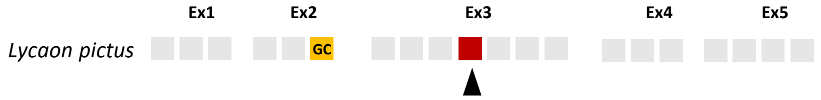

B

```
                                          10        20        30        40        50        60
                                 ....|....|....|....|....|....|....|....|....|....|....|....|...
HSA_GBA3_NM_020973.4_Exon 3      TGATTAAGGCTCATGCCAGATCCTGGCACAGCTATGATTCCTTATTTCGAAAAAAGCAGAAAGGTATG
                                    I  K  A  H  A  R  S  W  H  S  Y  D  S  L  F  R  K  K  Q  K  G  M
CM007414.1:87000000-87241457_M   TGATTAAAGCTCACGCCAGATCCTGGCACAGCTATAATTCCTTATTCCGAAGAGAGCAGAAGGGCATG
                                    I  K  A  H  A  R  S  W  H  S  Y  N  S  L  F  R  R  E  Q  K  G  M
gnl|SRA|SRR7759803.162700634.1   TGATTAAAGCTCACGCCAGATCCTGGCACAGCTATAA-------------------------------
                                    I  K  A  H  A  R  S  W  H  S  Y
gnl|SRA|SRR7874816.237434914.1   TGATTAAAGCTCACGCCAGATCCTGGCACAGCTAGAATT-----------------------------
                                    I  K  A  H  A  R  S  W  H  S  *  N
gnl|SRA|SRR8049198.395814872.1   TGATTAAAGCTCACGCCAGATCCTGGCACAGCTAGAATTC----------------------------
                                    I  K  A  H  A  R  S  W  H  S  *  N
gnl|SRA|SRR8049198.294556439.1   TGATTAAAGCTCACGCCAGATCCTGGCACAGCTAGAATTCCT--------------------------
                                    I  K  A  H  A  R  S  W  H  S  *  N  S
gnl|SRA|SRR7874816.60454900.2    TGATTAAAGCTCACGCCAGATCCTGGCACAGCTATAATTCCT--------------------------
                                    I  K  A  H  A  R  S  W  H  S  Y  N  S
gnl|SRA|SRR7874816.64312600.2_   TGATTAAAGCTCACGCCAGATCCTGGCACAGCTATAATTCCT--------------------------
                                    I  K  A  H  A  R  S  W  H  S  Y  N  S
gnl|SRA|SRR7874816.283512423.1   TGATTAAAGCTCACGCCAGATCCTGGCACAGCTATAATTCCTTA------------------------
                                    I  K  A  H  A  R  S  W  H  S  Y  N  S  L
gnl|SRA|SRR7874816.283512694.1   TGATTAAAGCTCACGCCAGATCCTGGCACAGCTATAATTCCTTA------------------------
                                    I  K  A  H  A  R  S  W  H  S  Y  N  S  L
gnl|SRA|SRR7874815.51446504.1_   TGATTAAAGCTCACGCCAGATCCTGGCACAGCTAGAATTCCTAGCT----------------------
                                    I  K  A  H  A  R  S  W  H  S  *  N  S  *
gnl|SRA|SRR2971441.33737515.1_   TGATTAAAGCTCACGCCAGATCCTGGCACAGCTATAATTCCTTATTC---------------------
                                    I  K  A  H  A  R  S  W  H  S  Y  N  S  L  F
gnl|SRA|SRR7874815.69286514.2    TGATTAAAGCTCACGCCAGATCCTGGCACAGCTAGAATTCCTTATTCCGAAG----------------
                                    I  K  A  H  A  R  S  W  H  S  *  N  S  L  F  R
gnl|SRA|SRR7874815.140771235.2   TGATTAAAGCTCACGCCAGATCCTGGCACAGCTAGAATTCCTTATTCCGAAG----------------
                                    I  K  A  H  A  R  S  W  H  S  *  N  S  L  F  R
gnl|SRA|SRR7874816.339536791.2   TGATTAAAGCTCACGCCAGATCCTGGCACAGCTATAATTCCTTATTCCGAAGAG--------------
                                    I  K  A  H  A  R  S  W  H  S  Y  N  S  L  F  R  R
gnl|SRA|SRR7874815.20757585.1    TGATTAAAGCTCACGCCAGATCCTGGCACAGCTATAATTCCTTATTCCGAAGAGAG------------
                                    I  K  A  H  A  R  S  W  H  S  Y  N  S  L  F  R  R  E
gnl|SRA|SRR7874816.209615481.1   TGATTAAAGCTCACGCCAGATCCTGGCACAGCTATAATTCCTTGTTCCGAAGAGAGCA----------
                                    I  K  A  H  A  R  S  W  H  S  Y  N  S  L  F  R  R  E
gnl|SRA|SRR7874815.122762155.1   TGATTAAAGCTCACGCCAGATCCTGGCACAGCTAGAATTCCTTATTCCGAAGAGAGCAG---------
                                    I  K  A  H  A  R  S  W  H  S  *  N  S  L  F  R  R  E  Q
gnl|SRA|SRR7874815.131213275.2   TGATTAAAGCTGACGCCAGATCCTGGCACAGCTAGAATTCCTTATTCCGAAGAGAACAG---------
                                    I  K  A  D  A  R  S  W  H  S  *  N  S  L  F  R  R  E  Q
gnl|SRA|SRR8049198.27549201.1    TGATTAAAGCTCACGCCAGATCCTGGCACAGCTAGAATTCCTTATTCCGAAGAGAGCAGAA-------
                                    I  K  A  H  A  R  S  W  H  S  *  N  S  L  F  R  R  E  Q
gnl|SRA|SRR7874816.24028064.1    TGATTAAAGCTCACGCCAGATCCTGGCACAGCTATAATTCCTTATTCCGAAGAGAGCAGAAGGG----
                                    I  K  A  H  A  R  S  W  H  S  Y  N  S  L  F  R  R  E  Q  K
gnl|SRA|SRR7874816.245548394.2   TGATTAAAGCTCACGCCAGATCCTGGCACAGCTATAATTCCTTATTCCGAAGAGAGCAGAAGGGCATG
                                    I  K  A  H  A  R  S  W  H  S  Y  N  S  L  F  R  R  E  Q  K  G  M
gnl|SRA|SRR7759803.50170212.1    TGATTAAAGCTCACGCCAGATCCTGGCACAGCTAGAATTCCTTATTCCGAAGAGAGCAGAAGGGCATG
                                    I  K  A  H  A  R  S  W  H  S  *  N  S  L  F  R  R  E  Q  K  G  M
gnl|SRA|SRR2971441.21195367.2    TGATTAAACCTCACGCCAGCTCCTGGCACAGCTATAATTACTTATTCCGAAAACAGCAGAAGGGCATG
                                    I  K  P  H  A  S  S  W  H  S  Y  N  Y  L  F  R  K  Q  Q  K  G  M
gnl|SRA|SRR2971441.1873522.1_(   TGATTAAAGCTCACGCCAGATCCTGGCACAGCTATAATTCCTTATTCCGAAGAGAGCAGAAGGGCATG
                                    I  K  A  H  A  R  S  W  H  S  Y  N  S  L  F  R  R  E  Q  K  G  M
gnl|SRA|SRR2971441.44627527.2_   TGATTAAAGCTCACGCCAGATCCTGGCACAGCTATAATTCCTTATTCCGAAGAGAGCAGAAGGGCATG
                                    I  K  A  H  A  R  S  W  H  S  Y  N  S  L  F  R  R  E  Q  K  G  M
gnl|SRA|SRR7874815.156879393.2   TGATTAAAGCTCACGCCAGATCCTGGCACAGCTAGAATTCCTTATTCCGAAGAGAGCAGAAGGGCATG
                                    I  K  A  H  A  R  S  W  H  S  *  N  S  L  F  R  R  E  Q  K  G  M
gnl|SRA|SRR2971441.54404470.1    TGATTAAAGCTCACGCCAGATCCTGGCACAGCTATAATTCCTTATTCCGAAGAGAGCAGAAGGGCATG
                                    I  K  A  H  A  R  S  W  H  S  Y  N  S  L  F  R  R  E  Q  K  G  M
gnl|SRA|SRR2971441.44627527.1    TGATTAAAGCTCACGCCAGATCCTGGCACAGCTATAATTCCTTATTCCGAAGAGAGCAGAAGGGCATG
                                    I  K  A  H  A  R  S  W  H  S  Y  N  S  L  F  R  R  E  Q  K  G  M
gnl|SRA|SRR2971441.1873522.2 1   TGATTAAAGCTCAGGCCAGATCCTGGCATAGCTATAATTCCTTATTCCGAAGAGAGCAGAAGGGCATG
                                    I  K  A  Q  A  R  S  W  H  S  Y  N  S  L  F  R  R  E  Q  K  G  M
```

C

Project	Run	Submitted by	DATE	Sample	Genotype
SRX4615470	SRR7759803	University of California at Los Angeles	2019-06-19	SAMN09924608 female	Heterozygous
SRX4713675	SRR7874816	Stanford University	2018-09-19	SAMN09917479 female	Heterozygous
SRX4713676	SRR7874815	Stanford University	2018-09-19	SAMN09917480 female	Heterozygous
SRX1460797	SRR2971441	Smithsonian Institution	2016-11-16	SAMN04312209 female	Homozygous coding
SRX4878889	SRR8049198	University of Copenhagen	2018-10-13	SAMN10180432	Heterozygous

Figure S25: **A**-Schematic representation of the identified mutations during gene annotation of GBA3 in *Lycaon pictus* using the available genome assembly in NCBI (Scaffold CM007414.1). Each group of grey squares represents an exon, number indicated above, yellow indicates loss of canonical splice site (AG-GT), green indicates insertion, blue indicates deletion and read indicates premature stop codon. Numbers in the squares indicate how many nucleotides were inserted or deleted or how many stop codons were identified. **B**-Validation of the identified mutation through multiple sequence alignment of reads obtained from SRA projects. Red box highlights the location of the premature stop codons. **C-**SRA projects consulted.

GBA3 annotation in Mustelidae and Mephitidae

A

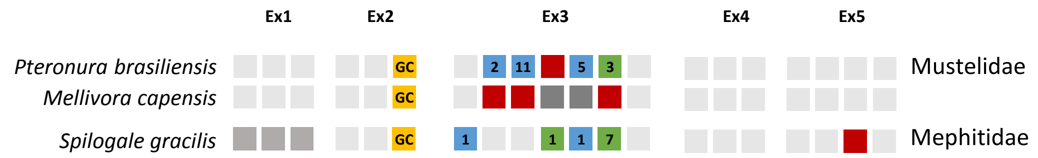

B

Specie	BioProject	Submitted by	DATE	Scaffold containing GBA3
Pteronura brasiliensis	PRJNA399365	Broad Institute	2019-01-15	PJEN01013838.1 – exon 1 & 2<br>PJEN01004883.1 – exon 3 & 4 & 5
Mellivora capensis	PRJNA399403	Broad Institute	2019-01-15	PISX010025557.1 - exon 2 & 3<br>PISX010021252.1 – exon 3 & 4<br>PISX010013454.1 – exon5<br>PISX010005505.1 – exon1
Spilogale gracilis	PRJNA399437	Broad Institute	2019-01-15	PITA01019296.1

Figure S26: **A**-Schematic representation of the identified mutations during gene annotation of GBA3 in *Pteronura brasiliensis*, *Mellivora capensis* and *Spilogale gracilis* . Each group of grey squares represents an exon, number indicated above, yellow indicates loss of canonical splice site (AG-GT), green indicates insertion, blue indicates deletion and read indicates premature stop codon. Numbers in the squares indicate how many nucleotides were inserted or deleted or how many stop codons were identified. **B**-Genome projects consulted and scaffold locations of the identified exons.

SUPPLEMENTARY MATERIAL- 6

Supplementary Material 6 - Validation of the identified mutations Chiroptera

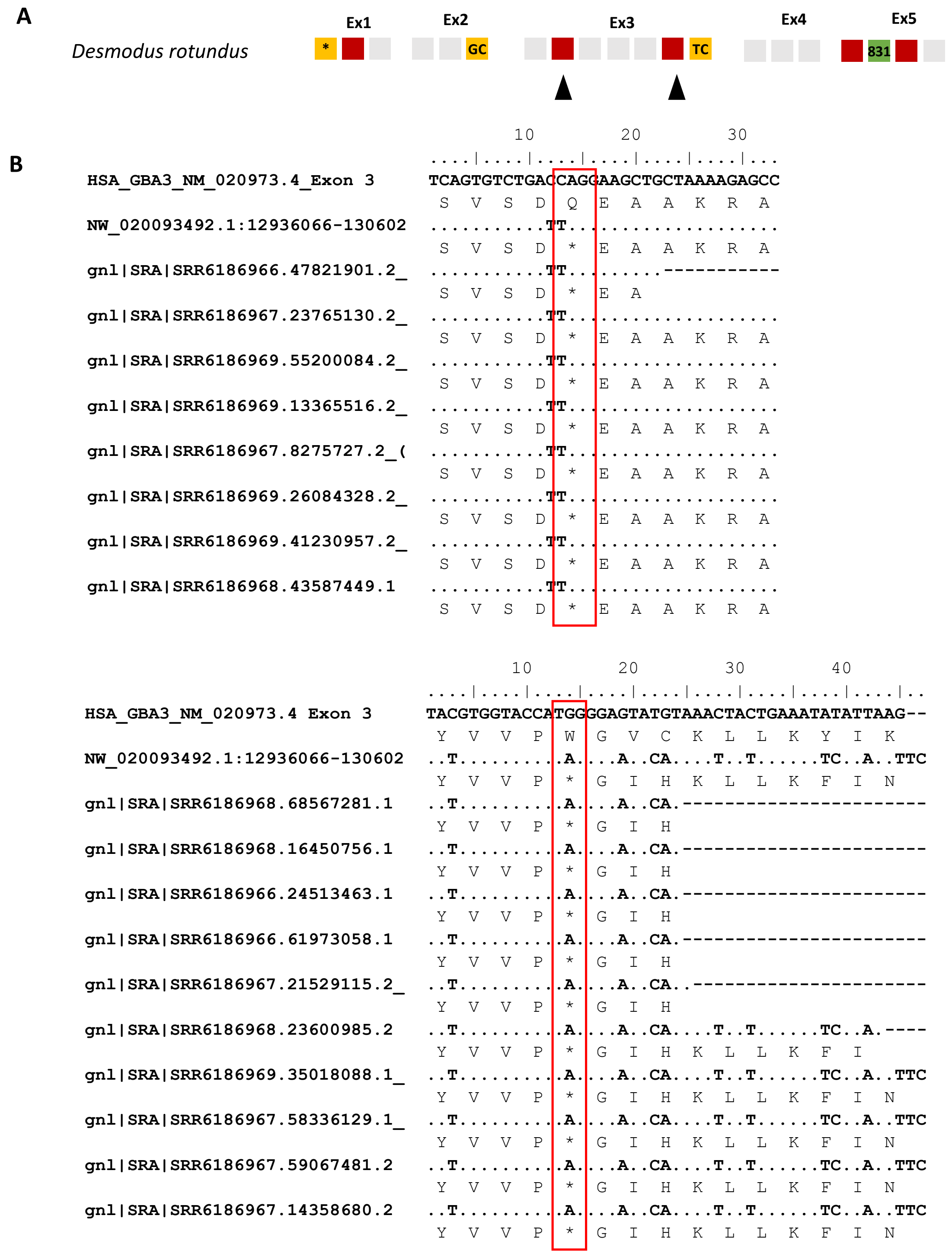

C

Project	Run	Submitted by	DATE	Sample
SRX3296991	SRR6186966	Beijing Genome Institute (BGI)	2018-02-26	SAMN07783852 DRU21DN04 male
SRX3296992	SRR6186967	Beijing Genome Institute (BGI)	2018-02-28	SAMN07783852 DRU21DN04 male
SRX3296991	SRR6186968	Beijing Genome Institute (BGI)	2018-02-26	SAMN07783852 DRU21DN04 male
SRX3296992	SRR6186969	Beijing Genome Institute (BGI)	2018-02-28	SAMN07783852 DRU21DN04 male

Figure S27: **A**-Schematic representation of the identified mutations during gene annotation of GBA3 in *Desmodus rotundus* using the available genome assembly in NCBI (Scaffold NW_020093492.1). Each group of grey squares represents an exon, number indicated above, yellow indicates loss of canonical splice site (AG-GT), green indicates insertion, blue indicates deletion and read indicates premature stop codon. Numbers in the squares indicate how many nucleotides were inserted or deleted or how many stop codons were identified. **B**-Validation of the identified mutation through multiple sequence alignment of reads obtained from SRA projects. Red box highlights the location of the premature stop codons. **C**-SRA projects consulted.

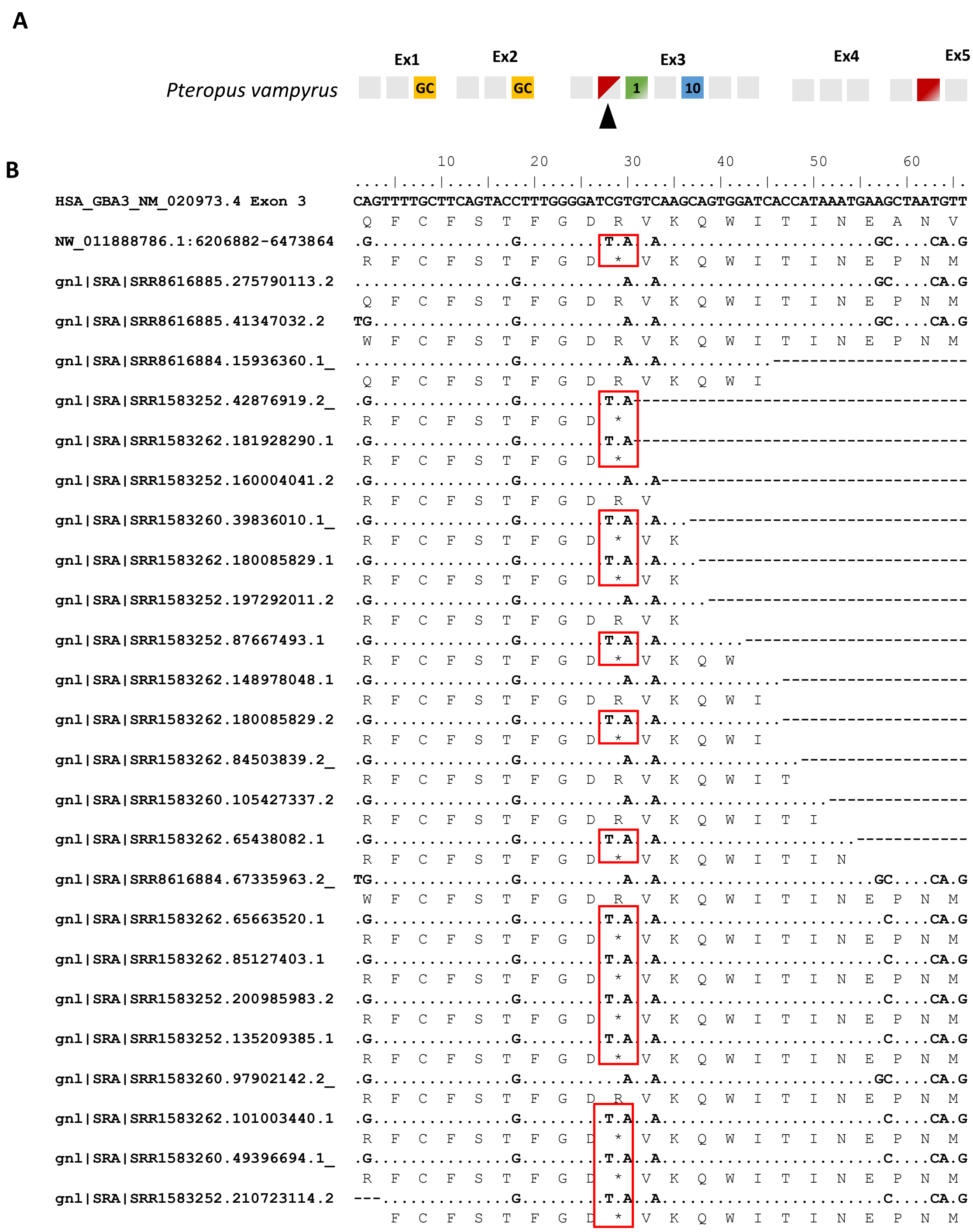

C

Project	Run	Submitted by	DATE	Sample	Genotype
SRX708169	SRR1583262	Baylor College of Medicine	2014-09-22	SAMN02894360 PVAM00 female	heterozygous
SRX708167	SRR1583260	Baylor College of Medicine	2014-09-22	SAMN02894360 PVAM00 female	heterozygous
SRX5415904	SRR8616885	Baylor College of Medicine	2019-02-24	SAMN10973750 0265 male	Homozygous non stop
SRX5415905	SRR8616884	Baylor College of Medicine	2019-02-24	SAMN10973750 0265 male	Homozygous non stop
SRX708167	SRR1583252	Baylor College of Medicine	2014-09-22	SAMN02894360 PVAM00 female	heterozygous

Figure S28 A-Schematic representation of the identified mutations during gene annotation of GBA3 in *Pteropus vampyrus* using the available genome assembly in NCBI (Scaffold NW_011888786.1). Each group of grey squares represents an exon, number indicated above, yellow indicates loss of canonical splice site (AG-GT), green indicates insertion, blue indicates deletion and read indicates premature stop codon. Numbers in the squares indicate how many nucleotides were inserted or deleted or how many stop codons were identified. **B**-Validation of the identified mutation through multiple sequence alignment of reads obtained from SRA projects. Red box highlights the location of the premature stop codons. **C**-SRA projects consulted.

A

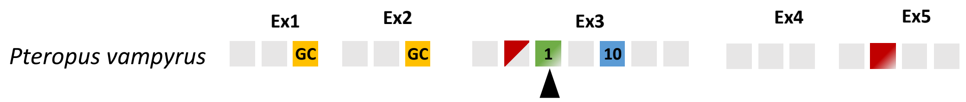

B

```
                                   10        20        30        40        50        60        70        80
                          ....|....|....|....|....|....|....|....|....|....|....|....|....|....|....|....|
HSA_GBA3_NM_020973.4_Exon_3       CAGGAAGCTGCTAAAAGAGCCATCACTTTCCATCTGGATTTATTT-GCTAAACCCATATTCATCGATGGTGATTATCCTG
                                   Q  E  A  A  K  R  A  I  T  F  H  L  D  L  F   A  K  P  I  F  I  D  G  D  Y  P
NW_011888786.1:6206882-6473864    CAGGAAGCTGCTAAAAGAGCCATGGCTTTTCACTTGGATTTTTTTTGCTAAACCTATATTTATTGATGGTGACTATCCTG
                                   Q  E  A  A  K  R  A  M  A  F  H  L  D  F  F  C  *  T  Y  I  Y  *  W  *  L  S  *
gnl|SRA|SRR1583262.134063914.2    CAGGAAGCTGCTAAAAGAGCCATGGCTTTTCACTTGGATTTTTTT-GCTAA-----------------------------
                                   Q  E  A  A  K  R  A  M  A  F  H  L  D  F  F   A
gnl|SRA|SRR1583252.54048823.1_    CAGGAAGCTGCTAAAAGAGCCATGGCTTTTCACTTGGATTTTTTTTGCTAAAC---------------------------
                                   Q  E  A  A  K  R  A  M  A  F  H  L  D  F  F  C  *
gnl|SRA|SRR1583252.135209385.2    CAGGAAGCTGCTAAAAGAGCCATGGCTTTTCACTTGGATTTTTTTTGCTAAACCTA------------------------
                                   Q  E  A  A  K  R  A  M  A  F  H  L  D  F  F  C  *  T
gnl|SRA|SRR8616884.31552352.2     CAGGAAGCTGCTAAAAGAGCCATGGCTTTTCACTTGGATTTTTTTTGCTAAACCTATAT---------------------
                                   Q  E  A  A  K  R  A  M  A  F  H  L  D  F  F  C  *  T  Y
gnl|SRA|SRR8616884.27796172.2     CAGGAAGCTGCTAAAAGAGCCATGGCTTTTCACTTGGATTTTTTTTGCTAAACCTATAT---------------------
                                   Q  E  A  A  K  R  A  M  A  F  H  L  D  F  F  C  *  T  Y
gnl|SRA|SRR1583260.102298166.2    CAGGAAGCTGCTAAAAGAGCCATGGCTTTTCACTTGGATTTTTTTTGCTAAACCTATATTT-------------------
                                   Q  E  A  A  K  R  A  M  A  F  H  L  D  F  F  C  *  T  Y  I
gnl|SRA|SRR1583262.121199683.1    CAGGAAGCTGCTAAAAGAGCCATGGCTTTTCACTTGGGGGTTTTT-GCTAAACCTATATTTA------------------
                                   Q  E  A  A  K  R  A  M  A  F  H  L  G  V  F   A  K  P  I  F
gnl|SRA|SRR1583252.221808407.1    CAGGAAGCTGCTAAAAGAGCCATGGCTTTTCACTTGGATTTTTTT-GCTAAACCTATATTTA------------------
                                   Q  E  A  A  K  R  A  M  A  F  H  L  D  F  F   A  K  P  I  F
gnl|SRA|SRR8616885.211205339.1    CAGGAAGCTGCTAAAAGAGCCATGGCTTTTCACTTGGATTTTTTTTGCTAAACCTATATTTAT-----------------
                                   Q  E  A  A  K  R  A  M  A  F  H  L  D  F  F  C  *  T  Y  I  Y
gnl|SRA|SRR8616885.64859546.2     CAGGAAGCTGCTAAAAGAGCCATGGCTTTTCACTTGGATTTTTTTTGCTAAACCTATATTTATTGATGGT----------
                                   Q  E  A  A  K  R  A  M  A  F  H  L  D  F  F  C  *  T  Y  I  Y  *  W
gnl|SRA|SRR1583262.83189639.2     CAGGAAGCTGCTAAAAGAGCCATGGCTTTTCACTTGGATTTTTTT-GCTAAACCTATATTTATTGATGGTG---------
                                   Q  E  A  A  K  R  A  M  A  F  H  L  D  F  F   A  K  P  I  F  I  D  G
gnl|SRA|SRR8616885.28672488.2_    CAGGAAGCTGCTAAAAGAGCCATGGCTTTTCACTTGGATTTTTTTTGCTAAACCTATATTTATTGATGGTG---------
                                   Q  E  A  A  K  R  A  M  A  F  H  L  D  F  F  C  *  T  Y  I  Y  *  W
gnl|SRA|SRR8616885.175831983.2    CAGGAAGCTGCTAAAAGAGCCATGGCTTTTCACTTGGATTTTTTTTGCTAAACCTATATTTATTGATGGTGAC-------
                                   Q  E  A  A  K  R  A  M  A  F  H  L  D  F  F  C  *  T  Y  I  Y  *  W  *
gnl|SRA|SRR8616885.39805943.1     CAGGAAGCTGCTAAAAGAGCCATGGCTTTTCACTTGGATTTTTTTTGCTAAACCTATATTTATTGATGGTGACTAT----
                                   Q  E  A  A  K  R  A  M  A  F  H  L  D  F  F  C  *  T  Y  I  Y  *  W  *  L
gnl|SRA|SRR1583252.41686883.2_    CAGGAAGCTGCTAAAAGAGCCATGGCTTTTCACTTGGATTTTTTT-GCTAAACCTATATTTATTGATGGTGACTATC---
                                   Q  E  A  A  K  R  A  M  A  F  H  L  D  F  F   A  K  P  I  F  I  D  G  D  Y
gnl|SRA|SRR1583252.119495504.2    CAGGAAGCTGCTAAAAGAGCCATGGCTTTTCACTTGGATTTTTTT-GCTAAACCTATATTTATTGATGGTGACTATCCT-
                                   Q  E  A  A  K  R  A  M  A  F  H  L  D  F  F   A  K  P  I  F  I  D  G  D  Y  P
gnl|SRA|SRR1583252.171436303.1    CAGGAAGCTGCTAAAAGAGCCATGGCTTTTCACTTGGATTTTTTT-GCTAAACCTATATTTATTGATGGTGACTATCCTG
                                   Q  E  A  A  K  R  A  M  A  F  H  L  D  F  F   A  K  P  I  F  I  D  G  D  Y  P
gnl|SRA|SRR1583260.61812250.2_    CAGGAAGCTGCTAAAAGAGCCATGGCTTTTCACTTGGATTTTTTT-GCTAAACCTATATTTATTGATGGTGACTATCCTG
                                   Q  E  A  A  K  R  A  M  A  F  H  L  D  F  F   A  K  P  I  F  I  D  G  D  Y  P
gnl|SRA|SRR8616885.11471787.2_    CAGGAAGCTGCTAAAAGAGCCATGGCTTTTCACTTGGATTTTTTTTGCTAAACCTATATTTATTGATGGTGACTATCCTG
                                   Q  E  A  A  K  R  A  M  A  F  H  L  D  F  F  C  *  T  Y  I  Y  *  W  *  L  S  *
gnl|SRA|SRR1583262.103372725.2    CAGGAAGCTGCTAAAAGAGCCATGGCTTTTCACTTGGATTTTTTT-GCTAAACCTATATTTATTGATGGTGACTATCCTG
                                   Q  E  A  A  K  R  A  M  A  F  H  L  D  F  F   A  K  P  I  F  I  D  G  D  Y  P
gnl|SRA|SRR1583252.34753233.1     CAGGAAGCTGCTAAAAGAGCCATGGCTTTTCACTTGGATTTTTTT-GCTAAACCTATATTTATTGATGGTGACTATCCTG
                                   Q  E  A  A  K  R  A  M  A  F  H  L  D  F  F   A  K  P  I  F  I  D  G  D  Y  P
gnl|SRA|SRR1583252.139240656.1    CAGGAAGCTGCTAAAAGAGCCATGGCTTTTCACTTGGATTTTTTT-GCTAAACCTATATTTATTGATGGTGACTATCCTG
                                   Q  E  A  A  K  R  A  M  A  F  H  L  D  F  F   A  K  P  I  F  I  D  G  D  Y  P
gnl|SRA|SRR8616885.56706837.2_    CAGGAAGCTGCTAAAAGAGCCATGACTTTTCACTTGGATTTTTTTTGCTAAACCTATATTTAGTGATGGTGACTATCCTG
                                   Q  E  A  A  K  R  A  M  T  F  H  L  D  F  F  C  *  T  Y  I  *  *  W  *  L  S  *
gnl|SRA|SRR8616885.286767882.1    CAGGAAGCTGCTAAAAGAGCCATGGCTTTTCACTTGGATTTTTTTTGCTAAACCTATATTTATTGATGGTGACTATCCTG
```

C

Project	Run	Submitted by	DATE	Sample	Genotype
SRX708169	SRR1583262	Baylor College of Medicine	2014-09-22	SAMN02894360 PVAM00 female	No insertion
SRX708167	SRR1583260	Baylor College of Medicine	2014-09-22	SAMN02894360 PVAM00 female	Heterozygous for 1 bp insertion
SRX5415904	SRR8616885	Baylor College of Medicine	2019-02-24	SAMN10973750 0265 male	Insertion 1 bp
SRX5415905	SRR8616884	Baylor College of Medicine	2019-02-24	SAMN10973750 0265 male	Insertion 1 bp
SRX708167	SRR1583252	Baylor College of Medicine	2014-09-22	SAMN02894360 PVAM00 female	Heterozygous for 1 bp insertion

Figure S29: **A**-Schematic representation of the identified mutations during gene annotation of GBA3 in *Pteropus vampyrus* using the available genome assembly in NCBI (Scaffold NW_011888786.1). Each group of grey squares represents an exon, number indicated above, yellow indicates loss of canonical splice site (AG-GT), green indicates insertion, blue indicates deletion and read indicates premature stop codon. Numbers in the squares indicate how many nucleotides were inserted or deleted or how many stop codons were identified. **B**-Validation of the identified mutation through multiple sequence alignment of reads obtained from SRA projects. Red box highlights the location of the 1bp insertion. **C**-SRA projects consulted.

A

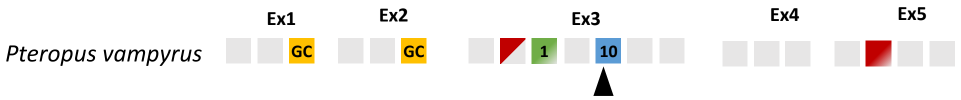

B

```
                                        10        20        30        40        50        60
                                ....|....|....|....|....|....|....|....|....|....|....|....|....
HSA_GBA3_NM_020973.4_Exon 3     TTTTTTCCAGATCCATCTTGGAAAAATGTGGATTGGATCTACGTGGTACCATGGGGAGTATGTAAACTA
                                 F  F  P  D  P  S  W  K  N  V  D  W  I  Y  V  V  P  W  G  V  C  K  L
NW_011888786.1:6206882-6473864  ATTTTTCCAGACCCATCT----------TGGATTGGATCTCTGTGGTGCCATGGGGACTACGTAAACTA
                                 I  F  P  D  P  S            W  I  G  S  L  W  C  H  G  D  Y  V  N
gnl|SRA|SRR8616885.257120797.1  ATTTTTCCAGACCCATCT----------TGGATTGGATCTCTGTGGTGCCATGGGGACTACGTAAACTA
                                 I  F  P  D  P  S            W  I  G  S  L  W  C  H  G  D  Y  V  N
gnl|SRA|SRR8616885.6427570.2_(  ATTTTTCCAGACCCATCT----------TGGATTGGATCTCTGTGGTGCCATGGGGACTACGTAAACTA
                                 I  F  P  D  P  S            W  I  G  S  L  W  C  H  G  D  Y  V  N
gnl|SRA|SRR8616885.6427764.2_(  ATTTTTCCAGACCCATCT----------TGGATTGGATCTCTGTGGTGCCATGGGGACTACGTAAACTA
                                 I  F  P  D  P  S            W  I  G  S  L  W  C  H  G  D  Y  V  N
gnl|SRA|SRR8616885.143701754.2  ATTTTTCCAGACCCATCT----------TGGATTGGATCTCTGTGGTGCCATGGGGACTACGTAAACTA
                                 I  F  P  D  P  S            W  I  G  S  L  W  C  H  G  D  Y  V  N
gnl|SRA|SRR1583252.227453636.1  ATTTTTCCAGACCCATCT----------TGGATTGGATCTCTGTGGTGCCATGGGGACTACGTAAACTA
                                 I  F  P  D  P  S            W  I  G  S  L  W  C  H  G  D  Y  V  N
gnl|SRA|SRR1583252.160232768.1  ATTTTTCCAGACCCATCT----------TGGATTGGATCTCTGTGGTGCCATGGGGACTACGTAAACTA
                                 I  F  P  D  P  S            W  I  G  S  L  W  C  H  G  D  Y  V  N
gnl|SRA|SRR1583262.101914349.2  ATTTTTCCAGACCCATCT----------TGGATTGGATCTCTGTGGTGCCATGGGGACTACGTAAACTA
                                 I  F  P  D  P  S            W  I  G  S  L  W  C  H  G  D  Y  V  N
gnl|SRA|SRR1583260.103887414.1  ATTTTTCCAGACCCATCT----------TGGATTGGATCTCTGTGGTGCCATGGGGACTACGTAAACTA
                                 I  F  P  D  P  S            W  I  G  S  L  W  C  H  G  D  Y  V  N
gnl|SRA|SRR1583252.152675465.2  ATTTTTCCAGACCCATCT----------TGGATTGGATCTCTGTGGTGCCATGGGGACTACGTAAACTA
                                 I  F  P  D  P  S            W  I  G  S  L  W  C  H  G  D  Y  V  N
gnl|SRA|SRR1583260.3313976.1_(  ATTTTTCCAGACCCATCT----------TGGATTGGATCTCTGTGGTGCCATGGGGACTACGTAAACTA
                                 I  F  P  D  P  S            W  I  G  S  L  W  C  H  G  D  Y  V  N
gnl|SRA|SRR1583252.10767610.1_  ATTTTTCCAGACCCATCT----------TGGATTGGATCTCTGTGGTGCCATGGGGACTACGTAAACTA
                                 I  F  P  D  P  S            W  I  G  S  L  W  C  H  G  D  Y  V  N
gnl|SRA|SRR1583262.92578007.2_  ATTTTTCCAGACCCATCT----------TGGATTGGATCTCTGTGGTGCCATGGGGACTACGTAAACTA
                                 I  F  P  D  P  S            W  I  G  S  L  W  C  H  G  D  Y  V  N
gnl|SRA|SRR8616884.45931017.2   ATTTTTCCAGACCCATCT----------TGGATTGGATCTCTGTGGTGCCATGGGGACTACGTAAACTA
                                 I  F  P  D  P  S            W  I  G  S  L  W  C  H  G  D  Y  V  N
gnl|SRA|SRR1583260.48785182.1   ATTTTTCCAGACCCATCT----------TGGATTGGATCTCTGTGGTGCCATGGGGACTACGTAAACTA
                                 I  F  P  D  P  S            W  I  G  S  L  W  C  H  G  D  Y  V  N
gnl|SRA|SRR1583260.45762962.1_  ATTTTTCCAGACCCATCT----------TGGATTGGATCTCTGTGGTGCCATGGGGACTACGTAAACTA
                                 I  F  P  D  P  S            W  I  G  S  L  W  C  H  G  D  Y  V  N
gnl|SRA|SRR1583260.61440587.1   ATTTTTCCAGACCCATCT----------TGGATTGGATCTCTGTGGTGCCATGGGGACTACGTAAACTA
                                 I  F  P  D  P  S            W  I  G  S  L  W  C  H  G  D  Y  V  N
gnl|SRA|SRR1583252.214075333.2  ATTTTTCCAGACCCATCT----------TGGATTGGATCTCTGTGGTGCCATGGGGACTACGTAAACTA
                                 I  F  P  D  P  S            W  I  G  S  L  W  C  H  G  D  Y  V  N
gnl|SRA|SRR1583260.50872389.1_  ATTTTTCCAGACCCATCT----------TGGATTGGATCTCTGTGGTGCCATGGGGACTACGTAAACTA
                                 I  F  P  D  P  S            W  I  G  S  L  W  C  H  G  D  Y  V  N
gnl|SRA|SRR1583252.227422489.2  ATTTTTCCAGACCCATCT----------TGGATTGGATCTCTGTGGTGCCATGGGGACTACGTAAACTA
                                 I  F  P  D  P  S            W  I  G  S  L  W  C  H  G  D  Y  V  N
gnl|SRA|SRR1583252.223572587.2  ATTTTTCCAGACCCATCT----------TGGATTGGATCTCTGTGGTGCCATGGGGACTACGTAAACTA
                                 I  F  P  D  P  S            W  I  G  S  L  W  C  H  G  D  Y  V  N
gnl|SRA|SRR1583262.33839901.1   ATTTTTCCAGACCCATCT----------TGGATTGGATCTCTGTGGTGCCATGGGGACTACGTAAACTA
                                 I  F  P  D  P  S            W  I  G  S  L  W  C  H  G  D  Y  V  N
gnl|SRA|SRR1583252.148365399.1  ATTTTTCCAGACCCATCT----------TGGATTGGATCTCTGTGGTGCCATGGTGACTACGTAAACTA
                                 I  F  P  D  P  S            W  I  G  S  L  W  C  H  G  D  Y  V  N
```

C

Project	Run	Submitted by	DATE	Sample	Genotype
SRX708169	SRR1583262	Baylor College of Medicine	2014-09-22	SAMN02894360 PVAM00 female	Deletion 10bp
SRX708167	SRR1583260	Baylor College of Medicine	2014-09-22	SAMN02894360 PVAM00 female	Deletion 10bp
SRX5415904	SRR8616885	Baylor College of Medicine	2019-02-24	SAMN10973750 0265 male	Deletion 10bp
SRX5415905	SRR8616884	Baylor College of Medicine	2019-02-24	SAMN10973750 0265 male	Deletion 10bp
SRX708167	SRR1583252	Baylor College of Medicine	2014-09-22	SAMN02894360 PVAM00 female	Deletion 10bp

Figure S30: **A**-Schematic representation of the identified mutations during gene annotation of GBA3 in *Pteropus vampyrus* using the available genome assembly in NCBI (Scaffold NW_011888786.1). Each group of grey squares represents an exon, number indicated above, yellow indicates loss of canonical splice site (AG-GT), green indicates insertion, blue indicates deletion and read indicates premature stop codon. Numbers in the squares indicate how many nucleotides were inserted or deleted or how many stop codons were identified. **B**-Validation of the identified mutation through multiple sequence alignment of reads obtained from SRA projects. Red box highlights the location of the 10bp deletions. **C.** SRA projects consulted.

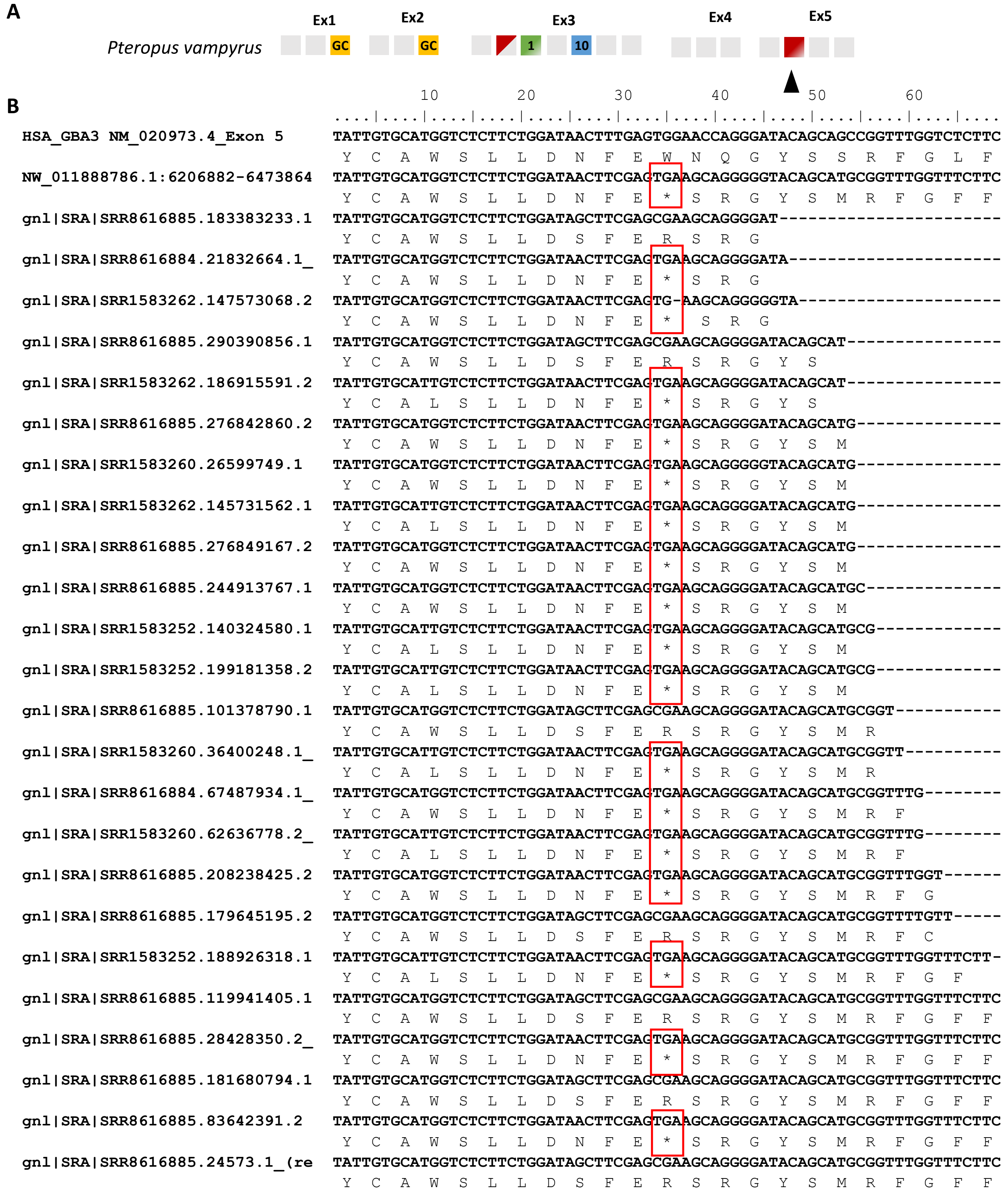

C

Project	Run	Submitted by	DATE	Sample	Genotype
SRX708169	SRR1583262	Baylor College of Medicine	2014-09-22	SAMN02894360 PVAM00 female	Stop codon
SRX708167	SRR1583260	Baylor College of Medicine	2014-09-22	SAMN02894360 PVAM00 female	Stop codon
SRX5415904	SRR8616885	Baylor College of Medicine	2019-02-24	SAMN10973750 0265 male	Heterozygous
SRX5415905	SRR8616884	Baylor College of Medicine	2019-02-24	SAMN10973750 0265 male	Stop codon
SRX708167	SRR1583252	Baylor College of Medicine	2014-09-22	SAMN02894360 PVAM00 female	Stop codon

Figure S31: **A**-Schematic representation of the identified mutations during gene annotation of GBA3 in *Pteropus vampyrus* using the available genome assembly in NCBI (Scaffold NW_011888786.1). Each group of grey squares represents an exon, number indicated above, yellow indicates loss of canonical splice site (AG-GT), green indicates insertion, blue indicates deletion and read indicates premature stop codon. Numbers in the squares indicate how many nucleotides were inserted or deleted or how many stop codons were identified. **B**-Validation of the identified mutation through multiple sequence alignment of reads obtained from SRA projects. Red box highlights the location of the premature stop codon. **C.** SRA projects consulted.

A

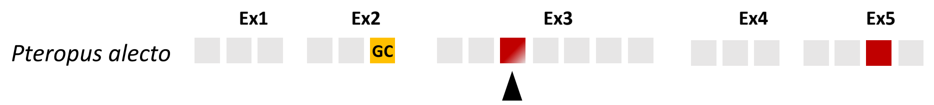

B

```
                                           10        20        30        40        50
                                  ....|....|....|....|....|....|....|....|....|....|....
HSA_GBA3_TV1_NM_020973.4 Exon 3   ACTGGAGGTTATCAGGCAGCTCATAATTTGATTAAGGCTCATGCCAGATCCTGG
                                   T  G  G  Y  Q  A  A  H  N  L  I  K  A  H  A  R  S  W
NW_006441953.1:c22607545-19415    ACGGGAGGCTATCAGGCCGCCCACAATTAGATTAAGGCCCATGCCAGATCCTGG
                                   T  G  G  Y  Q  A  A  H  N  *  I  K  A  H  A  R  S  W
gnl|SRA|SRR534483.94828246.2_e    ACGGGAGGCTATCAGGCCGCCCACAATTAGATTAAGGCCCATGCCAGATCCTGG
                                   T  G  G  Y  Q  A  A  H  N  *  I  K  A  H  A  R  S  W
gnl|SRA|SRR534483.60644302.2_e    ACGGGAGGCTATCAGGCCGCCCACAATTTGATTAAGGCCCATGCCAGATCCTGG
                                   T  G  G  Y  Q  A  A  H  N  L  I  K  A  H  A  R  S  W
gnl|SRA|SRR534540.100566160.2_    ACGGGAGGCTATCAGGCCGCCCACAATTAGATTAAGGCCCATGCCAGATCCTGG
                                   T  G  G  Y  Q  A  A  H  N  *  I  K  A  H  A  R  S  W
gnl|SRA|SRR534485.103681296.2_    ACGGGAGGCTATCAGGCCGCCCACAATTAGATTAAGGCCCATGCCAGATCCTGG
                                   T  G  G  Y  Q  A  A  H  N  *  I  K  A  H  A  R  S  W
gnl|SRA|SRR534483.138062223.1_    ACGGGAGGCTATCAGGCCGCCCACAATTTGATTAAGGCCCATGCCAGATCCTGG
                                   T  G  G  Y  Q  A  A  H  N  L  I  K  A  H  A  R  S  W
gnl|SRA|SRR534483.45559976.2_e    ACGGGAGGCTATCAGGCCGCCCACAATTTGATTAAGGCCCATGCCAGATCCTGG
                                   T  G  G  Y  Q  A  A  H  N  L  I  K  A  H  A  R  S  W
gnl|SRA|SRR534485.44643026.1_e    ACGGGAGGCTATCAGGCCGCCCACAATTAGATTAAGGCCCATGCCAGATCCTGG
                                   T  G  G  Y  Q  A  A  H  N  *  I  K  A  H  A  R  S  W
gnl|SRA|SRR534540.28693177.1_e    ACGGGAGGCTATCAGGCCGCCCACAATTAGATTAAGGCCCATGCCAGATCCTGG
                                   T  G  G  Y  Q  A  A  H  N  *  I  K  A  H  A  R  S  W
gnl|SRA|SRR534540.121093833.2_    ACGGGAGGCTATCAGGCCGCCCACAATTTGATTAAGGCCCATGCCAGATCCTGG
                                   T  G  G  Y  Q  A  A  H  N  L  I  K  A  H  A  R  S  W
gnl|SRA|SRR534483.24859747.1_e    ACGGGAGGCTATCAGGCCGCCCACAATTAGATTAAGGCCCATGCCAGATCCTGG
                                   T  G  G  Y  Q  A  A  H  N  *  I  K  A  H  A  R  S  W
gnl|SRA|SRR534483.133782317.1_    ACGGGAGGCTATCAGGCCGCCCACAATTAGATTAAGGCCCATGCCAGATCCTGG
                                   T  G  G  Y  Q  A  A  H  N  *  I  K  A  H  A  R  S  W
gnl|SRA|SRR534483.68807818.1_e    ACGGGAGGCTATCAGGCCGCCCACAATTTGATTAAGGCCCATGCCAGATCCTGG
                                   T  G  G  Y  Q  A  A  H  N  L  I  K  A  H  A  R  S  W
gnl|SRA|SRR534483.16711757.2_e    ACGGGAGGCTATCAGGCCGCCCACAATTAGATTAAGGCCCATGCCAGATCCTGG
                                   T  G  G  Y  Q  A  A  H  N  *  I  K  A  H  A  R  S  W
gnl|SRA|SRR534540.140210021.1_    ACGGGAGGCTATCAGGCCGCCCACAATTTGATTAAGGCCCATGCCAGATCCTGG
                                   T  G  G  Y  Q  A  A  H  N  L  I  K  A  H  A  R  S  W
gnl|SRA|SRR534485.155612114.1_    ACGGGAGGCTATCAGGCCGCCCACAATTAGATTAAGGCCCATGCCAGATCCTGG
                                   T  G  G  Y  Q  A  A  H  N  *  I  K  A  H  A  R  S  W
gnl|SRA|SRR534485.123133938.1_    TCGGGAGGCTATCAGGCCGCCCACAATTAGATTAAGGCCCATGCCAGATCCTGG
                                   S  G  G  Y  Q  A  A  H  N  *  I  K  A  H  A  R  S  W
gnl|SRA|SRR534540.31477883.2_e    ACGGGAGGCTATCAGGCCGCCCACAATTTGATTAAGGCCCATGCCAGATCCTGG
                                   T  G  G  Y  Q  A  A  H  N  L  I  K  A  H  A  R  S  W
gnl|SRA|SRR534483.145130297.1_    ACGGGAGGCTATCAGGCCGCCCACAATTTGATTAAGGCCCATGCCAGATCCTGG
                                   T  G  G  Y  Q  A  A  H  N  L  I  K  A  H  A  R  S  W
gnl|SRA|SRR534483.142027765.2_    ACGGGAGGCTATCAGGCCGCCCACAATTAGATTAAGGCCCATGCCAGATCCTGG
                                   T  G  G  Y  Q  A  A  H  N  *  I  K  A  H  A  R  S  W
gnl|SRA|SRR534485.113102105.2_    ACGGGAGGCTATCAGGCCGCCCACAATTTGATTAAGGCCCATGCCAGATCCTGG
                                   T  G  G  Y  Q  A  A  H  N  L  I  K  A  H  A  R  S  W
gnl|SRA|SRR534540.54084719.2_e    ACGGGAGCCTATCAGGCCGCCCACAATTTGATTAAGGCCCATGCCAGATCCTGG
                                   T  G  A  Y  Q  A  A  H  N  L  I  K  A  H  A  R  S  W
gnl|SRA|SRR534485.24370364.1_e    ACGGGAGGCTATCAGGCCGCCCACAATTAGATTAAGGCCCATGCCAGATCCTGG
                                   T  G  G  Y  Q  A  A  H  N  *  I  K  A  H  A  R  S  W
gnl|SRA|SRR534540.44597494.2_e    ACGGGAGGCTATCAGGCCGCCCACAATTAGATTAAGGCCCATGCCAGATCCTGG
                                   T  G  G  Y  Q  A  A  H  N  *  I  K  A  H  A  R  S  W
```

C

Project	Run	Submitted By	DATE	Sample
SRX174444	SRR534483	Beijing Genome Institute (BGI)	2015-07-22	SAMN01103203
SRX174446	SRR534485	Beijing Genome Institute (BGI)	2015-07-22	SAMN01103203
SRX174448	SRR534540	Beijing Genome Institute (BGI)	2015-07-22	SAMN01103203

Figure S32: A-Schematic representation of the identified mutations during gene annotation of GBA3 in *Pteropus alecto* using the available genome assemblies in NCBI (NW_006441953.1). Each group of grey squares represents an exon, number indicated above, yellow indicates loss of canonical splice site (AG-GT), green indicates insertion, blue indicates deletion and read indicates premature stop codon. Numbers in the squares indicate how many nucleotides were inserted or deleted or how many stop codons were identified. B- Validation of the identified mutation through multiple sequence alignment of reads obtained from SRA projects. Red box highlights the location of the premature stop codon. C. SRA projects consulted.

A

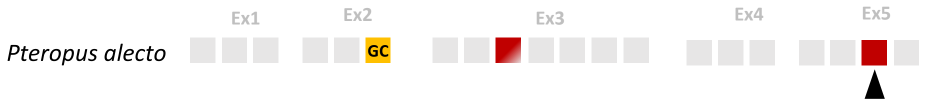

B

```
                                           10        20        30        40        50        60
                                   ....|....|....|....|....|....|....|....|....|....|....|....|....
HSA_GBA3_TV1_NM_020973.4 Exon 5   TATTGTGCATGGTCTCTTCTGGATAACTTTGAGTGGAACCAGGGATACAGCAGCCGGTTTGGTCTCTTC
                                     Y  C  A  W  S  L  L  D  N  F  E  W  N  Q  G  Y  S  S  R  F  G  L  F
NW_006441953.1:c22607545-19415    TATTGTGCATGGTCTCTTCTGGATAACTTCGAGTGAAGCAGGGGATACAGCATGCGGTTTGGTTTCTTC
                                     Y  C  A  W  S  L  L  D  N  F  E  *  S  R  G  Y  S  M  R  F  G  F  F
gnl|SRA|SRR534483.117908390.1_    TATTGTGCATGGTCTCTTCTGGATAACTTCGAGTGAAGCAGGGGATACAGCATGCGGTTTGGTTTCTTC
                                     Y  C  A  W  S  L  L  D  N  F  E  *  S  R  G  Y  S  M  R  F  G  F  F
gnl|SRA|SRR534540.82150004.1_e    TATTGTGCATGGTCTCTTCTGGATAACTTCGAGTGAAGCAGGGGATACAGAATGCGGTTTGTTTTCTTC
                                     Y  C  A  W  S  L  L  D  N  F  E  *  S  R  G  Y  R  M  R  F  V  F  F
gnl|SRA|SRR534485.170125593.1_    TATTGTGCATGGTCTCTTCTGGATAACTTCGAGTGAAGCAGGGGATACAGCATGCGGTTTGGTTTCTTC
                                     Y  C  A  W  S  L  L  D  N  F  E  *  S  R  G  Y  S  M  R  F  G  F  F
gnl|SRA|SRR534540.28790275.2_e    TATTGTGCATGGTCTCTTCTGGATAACTTCGAGTGAAGCAGGGGATACAGCATGCGGTTTGGTTTCTTC
                                     Y  C  A  W  S  L  L  D  N  F  E  *  S  R  G  Y  S  M  R  F  G  F  F
gnl|SRA|SRR534540.46630364.1_e    TATTGTGCATGGTCTCTTCTGGATAACTTCGAGTGAAGCAGGGGATACAGCATGCGGTTTGGTTTCTTC
                                     Y  C  A  W  S  L  L  D  N  F  E  *  S  R  G  Y  S  M  R  F  G  F  F
gnl|SRA|SRR534485.67386504.1_e    TATTGTGCATGGTCTCTTCTGGATAACTTCGAGTGAAGCAGGGGATACAGCATGCGGTTTGGTTTCTTC
                                     Y  C  A  W  S  L  L  D  N  F  E  *  S  R  G  Y  S  M  R  F  G  F  F
gnl|SRA|SRR534483.144726678.1_    TATTGTGCATGGTCTCTTCTGGATAACTTCGAGTGAAGCAGGGGATACAGCATGCGGTTTGGTTTCTTC
                                     Y  C  A  W  S  L  L  D  N  F  E  *  S  R  G  Y  S  M  R  F  G  F  F
gnl|SRA|SRR534485.125054347.2_    TATTGTGCATGGTCTCTTCTGGATAACTTCGAGTGAAGCAGGGGATACAGCATGCGGTTTGGTTTCTTC
                                     Y  C  A  W  S  L  L  D  N  F  E  *  S  R  G  Y  S  M  R  F  G  F  F
gnl|SRA|SRR534540.134520678.1_    TATTGTGCATGGTCTCTTCTGGATAACTTCGAGTGAAGAAGGGGATACAGCATGCGGTTTGGTTTCTTC
                                     Y  C  A  W  S  L  L  D  N  F  E  *  R  R  G  Y  S  M  R  F  G  F  F
gnl|SRA|SRR534540.51096177.1_e    TATTGTGCATGGTCTCTTCTGGATAACTTCGAGTGAAGCAGGGGATACAGCATGCGGTTTGGTTTCTTC
                                     Y  C  A  W  S  L  L  D  N  F  E  *  S  R  G  Y  S  M  R  F  G  F  F
gnl|SRA|SRR534485.17290152.2_e    TATTGTGCATGGTCTCTTCTGGATAACTTCGAGTGAAGCAGGGGATACAGCATGCGGTTTGGTTTCTTC
                                     Y  C  A  W  S  L  L  D  N  F  E  *  S  R  G  Y  S  M  R  F  G  F  F
gnl|SRA|SRR534485.34132399.1_e    TATTGTGCATGGTCTCTTCTGGATAACTTCGAGTGAAGCAGGGGATACAGCATGCGGTTTGGTTTCTTC
                                     Y  C  A  W  S  L  L  D  N  F  E  *  S  R  G  Y  S  M  R  F  G  F  F
gnl|SRA|SRR534485.100287928.2_    TATTGTGCATGGTCTCTTCTGGATAACTTCGAGTGAAGCAGGGGATACAGCATGCGGTTTGGTTTCTTC
                                     Y  C  A  W  S  L  L  D  N  F  E  *  S  R  G  Y  S  M  R  F  G  F  F
gnl|SRA|SRR534483.101479260.2_    TATTGTGCATGGTCTCTTCTGGATAACTTCGAGTGAAGCAGGGGATACAGCATGCGGTTTGGTTTCTTC
                                     Y  C  A  W  S  L  L  D  N  F  E  *  S  R  G  Y  S  M  R  F  G  F  F
gnl|SRA|SRR534485.130135968.1_    TATTGTGCATGGTCTCTTCTGGATAACTTCGAGTGAAGCAGGGGATACAGCATGCGGTTTGGTTTCTTC
                                     Y  C  A  W  S  L  L  D  N  F  E  *  S  R  G  Y  S  M  R  F  G  F  F
gnl|SRA|SRR534483.3620923.1_ex    TATTGTGCATGGTCTCTTCTGGATAACTTCGAGTGAAGCAGGGGATACAGCATGCGGTTTGGTTTCTTC
                                     Y  C  A  W  S  L  L  D  N  F  E  *  S  R  G  Y  S  M  R  F  G  F  F
gnl|SRA|SRR534485.34818661.2_e    TATTGTGCATGGTCTCTTCTGGATAACTTCGAGTGAAGCAGGGGATACAGCATGCGGTTTGGTTTCTTC
                                     Y  C  A  W  S  L  L  D  N  F  E  *  S  R  G  Y  S  M  R  F  G  F  F
gnl|SRA|SRR534483.130633479.1_    TATTGTGCATGGTCTCTTCTGGATAACTTCGAGTGAAGCAGGGGATACAGCATGCGGTTTGGTTTCTTC
                                     Y  C  A  W  S  L  L  D  N  F  E  *  S  R  G  Y  S  M  R  F  G  F  F
```

C

Project	Run	Submitted By	DATE	Sample
SRX174444	SRR534483	Beijing Genome Institute (BGI)	2015-07-22	SAMN01103203
SRX174446	SRR534485	Beijing Genome Institute (BGI)	2015-07-22	SAMN01103203
SRX174448	SRR534540	Beijing Genome Institute (BGI)	2015-07-22	SAMN01103203

Figure S33: A-Schematic representation of the identified mutations during gene annotation of GBA3 in *Pteropus alecto* using the available genome assemblies in NCBI (NW_006441953.1). Each group of grey squares represents an exon, number indicated above, yellow indicates loss of canonical splice site (AG-GT), green indicates insertion, blue indicates deletion and read indicates premature stop codon. Numbers in the squares indicate how many nucleotides were inserted or deleted or how many stop codons were identified. B- Validation of the identified mutation through multiple sequence alignment of reads obtained from SRA projects. Red box highlights the location of the premature stop codon. C. SRA projects consulted.

SUPPLEMENTARY MATERIAL- 7

Supplementary Material 7 -Validation of the identified mutations in Afrotheria

A

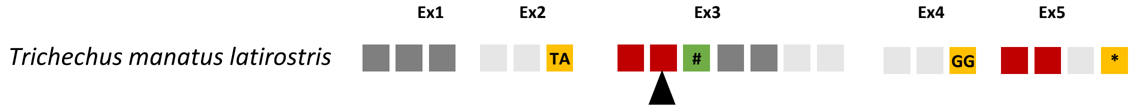

B

```
                                          10        20        30        40        50
                                 ....|....|....|....|....|....|....|....|....|....|....|...
HSA_GBA3_NM_020973.4 Exon 3      GCTCATAATTTGATTAAGGCTCATGCCAGATCCTGGCACAGCTATGATTCCTTATTTC
                                  A  H  N  L  I  K  A  H  A  R  S  W  H  S  Y  D  S  L  F
NW_004443970.1_extraction_(rev   GCTCATAATTTGATTAAGGCACATGCCTGATCCTGGCACAGCTATGATTCCTTATTCC
                                  A  H  N  L  I  K  A  H  A  *  S  W  H  S  Y  D  S  L  F
gnl|SRA|SRR353369.16372546.2_(   GCTCATAATTTGATTAAGGCACATGCCTGATCCTGGCACAGCTATGATTCCTTATTCC
                                  A  H  N  L  I  K  A  H  A  *  S  W  H  S  Y  D  S  L  F
gnl|SRA|SRR353369.16372638.2_(   GCTCATAATTTGATTAAGGCACATGCCTGATCCTGGCACAGCTATGATTCCTTATTCC
                                  A  H  N  L  I  K  A  H  A  *  S  W  H  S  Y  D  S  L  F
gnl|SRA|SRR353370.17264331.2_(   GCTCATAATTTGATTAAGGCACATGCCTGATCCTGGCACAGGTATGATTCCTTATTCC
                                  A  H  N  L  I  K  A  H  A  *  S  W  H  R  Y  D  S  L  F
gnl|SRA|SRR353370.17264331.2_(   GCTCATAATTTGATTAAGGCACATGCCTGATCCTGGCACAGGTATGATTCCTTATTCC
                                  A  H  N  L  I  K  A  H  A  *  S  W  H  R  Y  D  S  L  F
gnl|SRA|SRR353369.16372546.2_(   GCTCATAATTTGATTAAGGCACATGCCTGATCCTGGCACAGCTATGATTCCTTATTCC
                                  A  H  N  L  I  K  A  H  A  *  S  W  H  S  Y  D  S  L  F
gnl|SRA|SRR353369.16372638.2_(   GCTCATAATTTGATTAAGGCACATGCCTGATCCTGGCACAGCTATGATTCCTTATTCC
                                  A  H  N  L  I  K  A  H  A  *  S  W  H  S  Y  D  S  L  F
gnl|SRA|SRR353370.17264241.2_(   GCTCATAATTTGATTAAGGCACATGCCTGATCCTGGCACAGCTATGATTCCTTATTCC
                                  A  H  N  L  I  K  A  H  A  *  S  W  H  S  Y  D  S  L  F
gnl|SRA|SRR353370.17264241.2_(   GCTCATAATTTGATTAAGGCACATGCCTGATCCTGGCACAGCTATGATTCCTTATTCC
                                  A  H  N  L  I  K  A  H  A  *  S  W  H  S  Y  D  S  L  F
gnl|SRA|SRR353371.30954787.2_(   GCTCATAATTTGATTAAGGCAAATGCCTGATCCTGGCACAGCTATGATTCCTTATTCC
                                  A  H  N  L  I  K  A  N  A  *  S  W  H  S  Y  D  S  L  F
gnl|SRA|SRR353371.30954787.2_(   GCTCATAATTTGATTAAGGCAAATGCCTGATCCTGGCACAGCTATGATTCCTTATTCC
                                  A  H  N  L  I  K  A  N  A  *  S  W  H  S  Y  D  S  L  F
gnl|SRA|SRR331145.212800016.3_   GCTCATAATTTGATTAAGGCACATGCCTGATCCTGGCACAGCTATGATTCCTTATTCC
                                  A  H  N  L  I  K  A  H  A  *  S  W  H  S  Y  D  S  L  F
gnl|SRA|SRR331145.81425312.3_(   GCTCATAATTTGATTAAGGCACATGCCTGATCCTGGCACAGCTATGATTCCTTATTCC
                                  A  H  N  L  I  K  A  H  A  *  S  W  H  S  Y  D  S  L  F
gnl|SRA|SRR331145.228359526.3    GCTCATAATTTGATTAAGGCACATGCCTGATCCTGGCACAGCTATGATTCCTTATTCC
                                  A  H  N  L  I  K  A  H  A  *  S  W  H  S  Y  D  S  L  F
```

C

Project	Run	Submitted by	DATE	Sample
SRX091954	SRR331145	Broad Institute (BI)	2011-08-17	SAMN00632092 - Lorelei
SRX101108	SRR353371	Broad Institute (BI)	2011-10-16	SAMN00632092 - Lorelei
SRX101108	SRR353370	Broad Institute (BI)	2011-10-16	SAMN00632092 - Lorelei
SRX101108	SRR353369	Broad Institute (BI)	2011-10-16	SAMN00632092 - Lorelei

Figure S34: **A**-Schematic representation of the identified mutations during gene annotation of GBA3 in *Trichechus manatus latirostris* using the available genome assembly in NCBI (Scaffold NW_004443970.1). Each group of grey squares represents an exon, number indicated above, yellow indicates loss of canonical splice site (AG-GT), green indicates insertion, blue indicates deletion and read indicates premature stop codon. Numbers in the squares indicate how many nucleotides were inserted or deleted or how many stop codons were identified. **B**-Validation of the identified mutation through multiple sequence alignment of reads obtained from SRA projects. Red box highlights the location of the premature stop codon. **C.** SRA projects consulted.

A

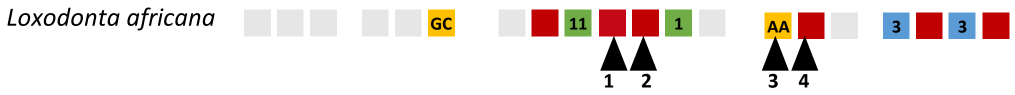

B

```
                                   10        20        30        40        50        60
                                ....|....|....|....|....|....|....|....|....|....|....|....|...
HSA_GBA3_TV1_NM_020973.4 Exon 3 TTTTGCTTCAGTACCTTTGGGGATCGTGTCAAGCAGTGGATCACCATAAATGAAGCTAATGTTCTTTC (...)
                                 F  C  F  S  T  F  G  D  R  V  K  Q  W  I  T  I  N  E  A  N  V  L
NW_003573438.1:22523601-227226  TTTTGCTTCAGTACCTTTGAGGATTGATTCAAACAGTGGATCACCATAAATGAGACCAACCTTTTTGC (...)
                                 F  C  F  S  T  F  E  D  *  F  K  Q  W  I  T  I  N  E  T  N  L  F
ti:1798782419_extraction        TTTTGCTTCAGTACCTTTGAGGATTGATTCAAACAGTGGATCACCATAAATGAGACCAACCTTTTTGC (...)
                                 F  C  F  S  T  F  E  D  *  F  K  Q  W  I  T  I  N  E  T  N  L  F
ti:1798782419_extraction        TTTTGCTTCAGTACCTTTGAGGATTGATTCAAACAGTGGATCACCATAAATGAGACCAACCTTTTTGC (...)
                                 F  C  F  S  T  F  E  D  *  F  K  Q  W  I  T  I  N  E  T  N  L  F
ti:1798782419_extraction        TTTTGCTTCAGTACCTTTGAGGATTGATTCAAACAGTGGATCACCATAAATGAGACCAACCTTTTTGC (...)
                                 F  C  F  S  T  F  E  D  *  F  K  Q  W  I  T  I  N  E  T  N  L  F
ti:2132314586_extraction        TTTTGCTTCAGTACCTTTGAGGATTGATTCAAACAGTGGATCACCATAAATGAGACCAACCTTTTTGC (...)
                                 F  C  F  S  T  F  E  D  *  F  K  Q  W  I  T  I  N  E  T  N  L  F
ti:2132314586_extraction        TTTTGCTTCAGTACCTTTGAGGATTGATTCAAACAGTGGATCACCATAAATGAGACCAACCTTTTTGC (...)
                                 F  C  F  S  T  F  E  D  *  F  K  Q  W  I  T  I  N  E  T  N  L  F
ti:2135135942_extraction        TTTTGCTTCAGTACCTTTGAGGATTGATTCAAACAGTGGATCACCATAAATGAGACCAACCTTTTTGC (...)
                                 F  C  F  S  T  F  E  D  *  F  K  Q  W  I  T  I  N  E  T  N  L  F
ti:2135135942_extraction        TTTTGCTTCAGTACCTTTGAGGATTGATTCAAACAGTGGATCACCATAAATGAGACCAACCTTTTTGC (...)
                                 F  C  F  S  T  F  E  D  *  F  K  Q  W  I  T  I  N  E  T  N  L  F
                                                         1
```

```
                                   10        20        30        40        50        60        70
                                ....|....|....|....|....|....|....|....|....|....|....|....|....|....|.
HSA_GBA3_TV1_NM_020973.4 Exon 3 GCCAGATCCTGGCACAGCTATGATTCCTTATTTCGAAAAAA-GCAGAAAGGTATGGTGTCTCTATCACTTTTTGCG
                                 A  R  S  W  H  S  Y  D  S  L  F  R  K  K   Q  K  G  M  V  S  L  S  L  F  A
NW_003573438.1:22523601-227226  GCCCGATCCTGGTACAGCTATGATTCCTTGATCTGAAAAAGTGCAGAAGGATATTGTGTCCTTAGCACTTTTTTAT
                                 A  R  S  W  Y  S  Y  D  S  L  I  *  K  S  A  E  G  Y  C  V  L  S  T  F  L
ti:1798782419_extraction        GCCCGATCCTGGTACAGCTATGATTCCTTGATCTGAAAAAGTGCAGAAGGATATTGTGTCCTTAGCACTTTTTTAT
                                 A  R  S  W  Y  S  Y  D  S  L  I  *  K  S  A  E  G  Y  C  V  L  S  T  F  L
ti:1798782419_extraction        GCCCGATCCTGGTACAGCTATGATTCCTTGATCTGAAAAAGTGCAGAAGGATATTGTGTCCTTAGCACTTTTTTAT
                                 A  R  S  W  Y  S  Y  D  S  L  I  *  K  S  A  E  G  Y  C  V  L  S  T  F  L
ti:1798782419_extraction        GCCCGATCCTGGTACAGCTATGATTCCTTGATCTGAAAAAGTGCAGAAGGATATTGTGTCCTTAGCACTTTTTTAT
                                 A  R  S  W  Y  S  Y  D  S  L  I  *  K  S  A  E  G  Y  C  V  L  S  T  F  L
ti:2132314586_extraction        GCCCGATCCTGGTACAGCTATGATTCCTTGATCTGAAAAAGTGCAGAAGGATATTGTGTCCTTAGCACTTTTTTAT
                                 A  R  S  W  Y  S  Y  D  S  L  I  *  K  S  A  E  G  Y  C  V  L  S  T  F  L
ti:2132314586_extraction        GCCCGATCCTGGTACAGCTATGATTCCTTGATCTGAAAAAGTGCAGAAGGATATTGTGTCCTTAGCACTTTTTTAT
                                 A  R  S  W  Y  S  Y  D  S  L  I  *  K  S  A  E  G  Y  C  V  L  S  T  F  L
ti:2135135942_extraction        GCCCGATCCTGGTACAGCTATGATTCCTTGATCTGAAAAAGTGCAGAAGGATATTGTGTCCTTAGCACTTTTTTAT
                                 A  R  S  W  Y  S  Y  D  S  L  I  *  K  S  A  E  G  Y  C  V  L  S  T  F  L
ti:2135135942_extraction        GCCCGATCCTGGTACAGCTATGATTCCTTGATCTGAAAAAGTGCAGAAGGATATTGTGTCCTTAGCACTTTTTTAT
                                 A  R  S  W  Y  S  Y  D  S  L  I  *  K  S  A  E  G  Y  C  V  L  S  T  F  L
                                                                   2
```

C

Trace name	ti	DATE	Sample
BI81cbe18d833c17f91198d81279c09233.R.5.T0	1798782419	2007-06-13	ISIS 603380
BIbeb19cfd5e94c3fb9b04f621a69a7cde.R.2.T0	2132314586	2008-10-02	Born Wild Namibia
BId30522fe185e77fdaf897b6df474387d.F.2.T0	2135135942	2008-10-13	Born Wild Namibia

Figure S35: A-Schematic representation of the identified mutations during gene annotation of GBA3 in *Loxodonta africana* using the available genome assemblies in NCBI (NW_003573438.1). Each group of grey squares represents an exon, number indicated above, yellow indicates loss of canonical splice site (AG-GT), green indicates insertion, blue indicates deletion and read indicates premature stop codon. Numbers in the squares indicate how many nucleotides were inserted or deleted or how many stop codons were identified. B- Validation of the identified mutations through multiple sequence alignment of reads obtained from Trace archives. Red box highlights the location of the premature stop codons. C. Trace Archives and reads consulted.

A

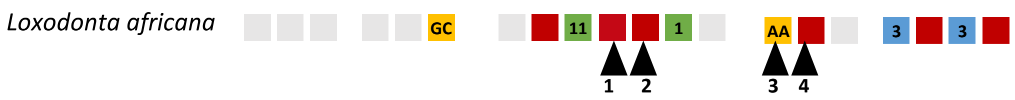

B

```
                                            10        20        30        40        50
                                    ....|....|....|....|....|....|....|....|....|....|...
HSA_GBA3_TV1_NM_020973.4 Exon 4  ------------GATACATATAATAACCCTGTAATTTACATCACTGAGAATGG
                                             D  T  Y  N  N  P  V  I  Y  I  T  E  N
NW_003573438.1:22523601-227226   CTCTTGTTCTAAGATACGTAAAATAACCCTGTAATTTACATCACTGAGAGTGG
                                             D  T  *  N  N  P  V  I  Y  I  T  E  S
ti:1741208847_extraction_(reve   CTCTTGTTCTAAGATACGTAAAATAACCCTGTAATTTACATCACTGAGAGTGG
                                             D  T  *  N  N  P  V  I  Y  I  T  E  S
ti:1707753808_extraction_(reve   CTCTTGTTCTAAGATACGTAAAATAACCCTGTAATTTACATCACTGAGAGTGG
                                             D  T  *  N  N  P  V  I  Y  I  T  E  S
ti:538690690_extraction_(rever   CTCTTGTTCTAAGATACGTAAAATAACCCTGTAATTTACATCACTGAGAGTGG
                                             D  T  *  N  N  P  V  I  Y  I  T  E  S
ti:1728514306_extraction BIf05   CTCTTGTTCTAAGATACGTAAAATAACCCTGTAATTTACATCACTGAGAGTGG
                                             D  T  *  N  N  P  V  I  Y  I  T  E  S
ti:2073211189_extraction_(reve   CTCTTGTTCTAAGATACGTAAAATAACCCTGTAATTTACATCACTGAGAGTGG
                                             D  T  *  N  N  P  V  I  Y  I  T  E  S
ti:529784319_extraction_(rever   CTCTTGTTCTAAGATACGTAAAATAACCCTGTAATTTACATCACTGAGAGTGG
                                             D  T  *  N  N  P  V  I  Y  I  T  E  S
ti:483678370_extraction G720P6   CTCTTGTTCTAAGATACGTAAAATAACCCTGTAATTTACATCACTGAGAGTGG
                                             D  T  *  N  N  P  V  I  Y  I  T  E  S
ti:510034393_extraction G720P6   CTCTTGTTCTAAGATACGTAAAATAACCCTGTAATTTACATCACTGAGAGTGG
                                             D  T  *  N  N  P  V  I  Y  I  T  E  S
                                             3        4
```

C

Trace name	ti	DATE	Sample
G720P613871RB10.T0	483678370	2004-07-19	LAF-12
G720P641812RB9.T0	538690690	2004-09-13	LAF-12
G720P638611FF7.T0	529784319	2004-09-07	LAF-12
G720P623062RD7.T0	510034393	2004-08-13	LAF-12
BI95c365c3a4744bba74f58abff52ee7e2.F.6.T0	2073211189	2008-05-02	ISIS 603380
BI81768ac186133c9ec81f369cce471370.R.6.T0	1707753808	2007-04-23	ISIS 603380
BIf055595bcc234ae349a40b65ac270540.F.6.T0	1728514306	2007-05-14	ISIS 603380
BIfb0464f154da5d3fe8dead95142a753b.F.6.T0	1741208847	2007-05-21	ISIS 603380

Figure S36: A-Schematic representation of the identified mutations during gene annotation of GBA3 in *Loxodonta africana* using the available genome assemblies in NCBI (NW_003573438.1). Each group of grey squares represents an exon, number indicated above, yellow indicates loss of canonical splice site (AG-GT), green indicates insertion, blue indicates deletion and read indicates premature stop codon. Numbers in the squares indicate how many nucleotides were inserted or deleted or how many stop codons were identified. B- Validation of the identified mutations through multiple sequence alignment of reads obtained from Trace archives. Red box highlights the location of the non- canonical AG-GT splice site and premature stop codon. C. Trace Archives and reads consulted.

SUPPLEMENTARY MATERIAL- 8

Supplementary Material 8- Validation of the identified mutations in *Neu2* Cetacea

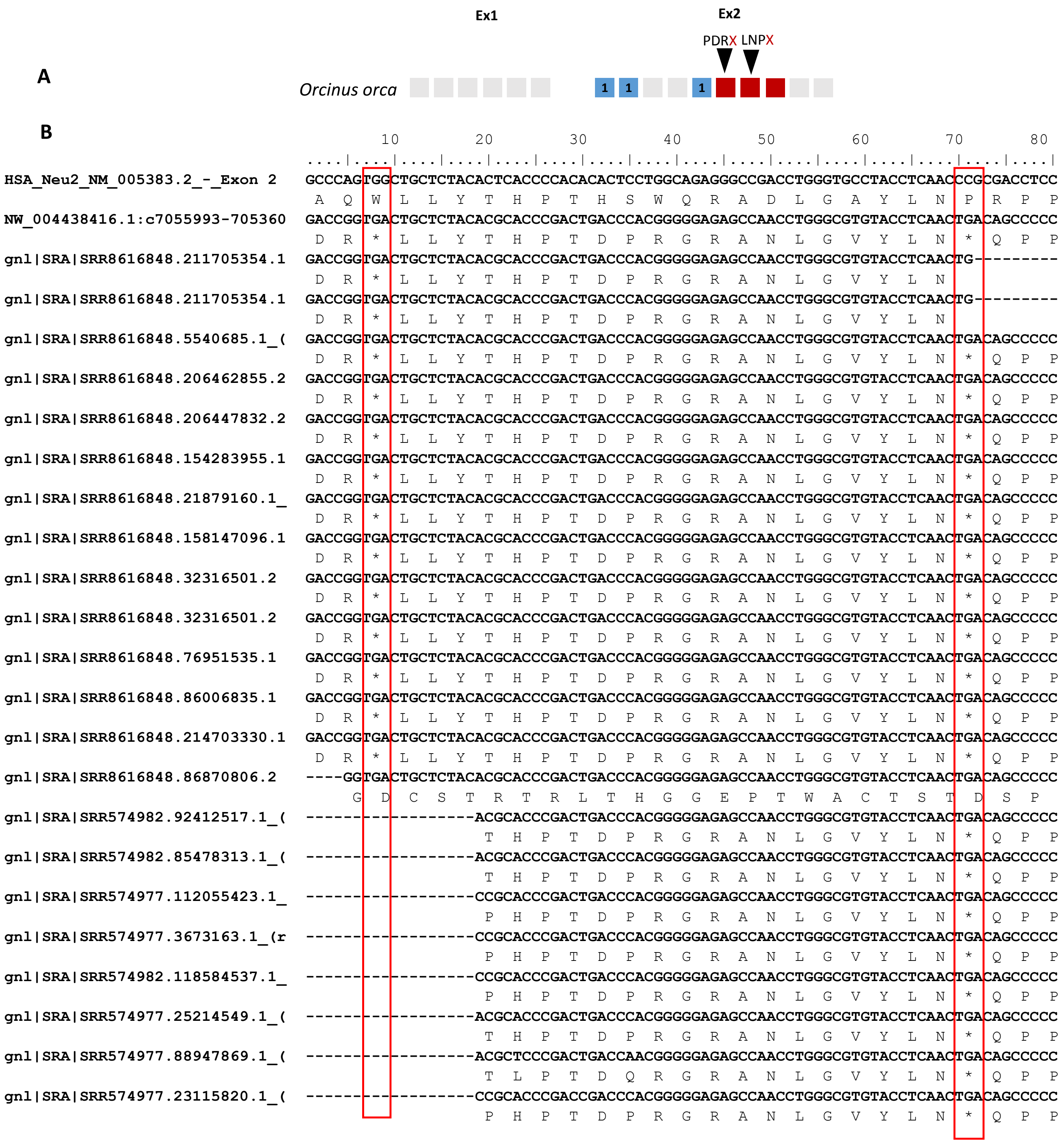

C

Project	Run	Submitted by	DATE	Sample
SRX188934	SRR574977	Baylor College of Medicine	2012-09-20	SAMN01180276 AForca1 female
SRX188934	SRR574982	Baylor College of Medicine	2012-09-20	SAMN01180276 AForca1 female
SRX5415941	SRR8616848	Baylor College of Medicine	2019-02-24	SAMN10973740 0193 female

Figure S37: **A**-Schematic representation of the identified mutations during gene annotation of *NEU2* in *Orcinus orca* using the available genome assembly in NCBI (Scaffold NW_004438416.1). Each group of grey squares represents an exon, number indicated above, yellow indicates loss of canonical splice site (AG-GT), green indicates insertion, blue indicates deletion and read indicates premature stop codon. Numbers in the squares indicate how many nucleotides were inserted or deleted or how many stop codons were identified. **B**-Validation of the identified mutation through multiple sequence alignment of reads obtained from SRA projects. Red box highlights the location of the premature stop codon. **C.** SRA projects consulted.

A

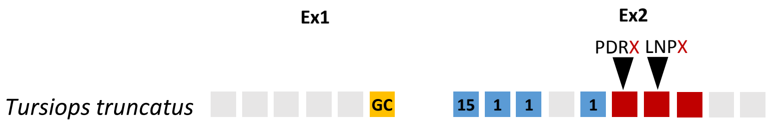

B

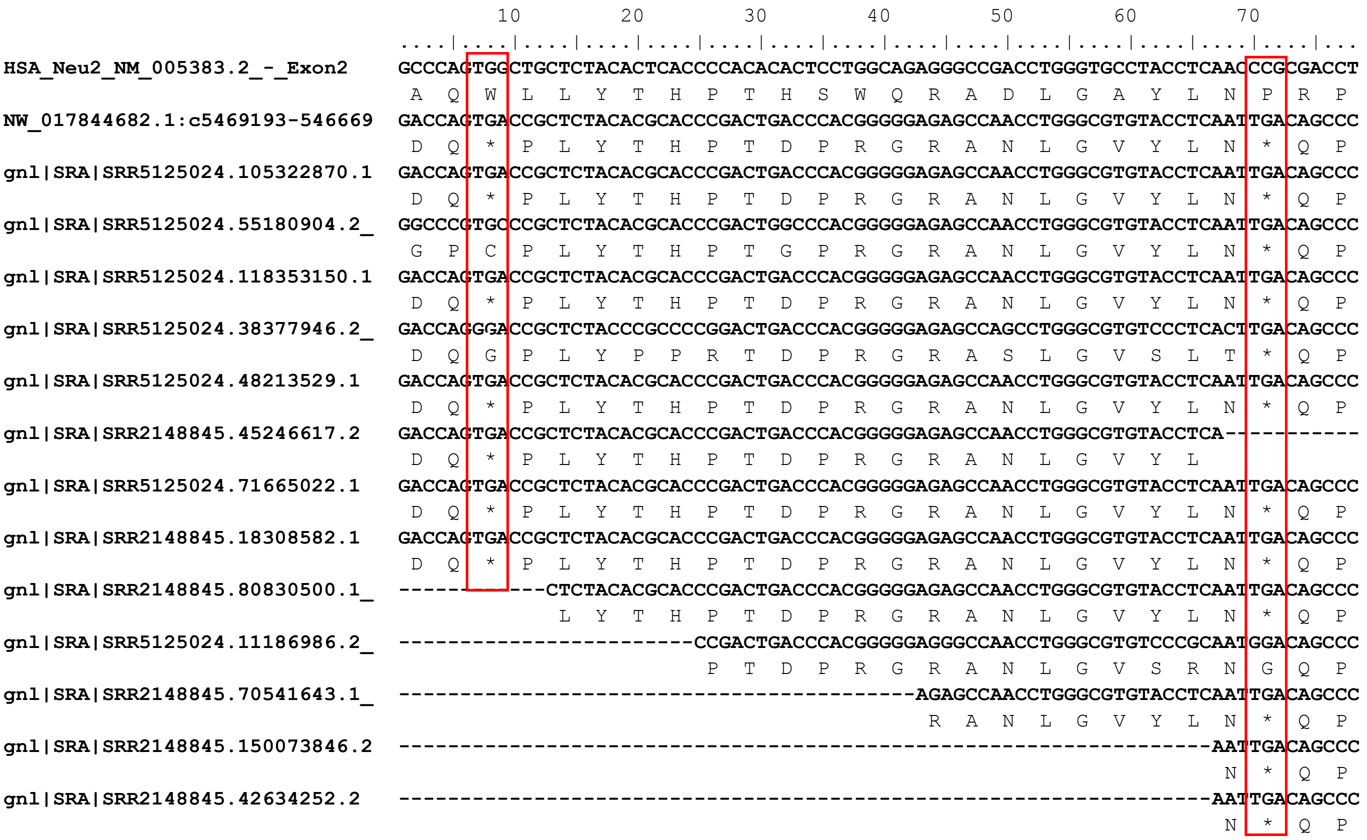

C

Project	Run	Submitted by	DATE	Sample
SRX1136400	SRR2148845	Beijing Genome Institute	2016-08-10	SAMN03968479
SRX2439826	SRR5125024	National Institute of Standards and Technology	2016-12-27	SAMN06114300 ACU285439A female

Figure S38: **A**-Schematic representation of the identified mutations during gene annotation of *NEU2* in *Tursiops truncatus* using the available genome assembly in NCBI (Scaffold NW_017844682.1). Each group of grey squares represents an exon, number indicated above, yellow indicates loss of canonical splice site (AG-GT), green indicates insertion, blue indicates deletion and read indicates premature stop codon. Numbers in the squares indicate how many nucleotides were inserted or deleted or how many stop codons were identified. **B**-Validation of the identified mutation through multiple sequence alignment of reads obtained from SRA projects. Red box highlights the location of the premature stop codon. **C.** SRA projects consulted.

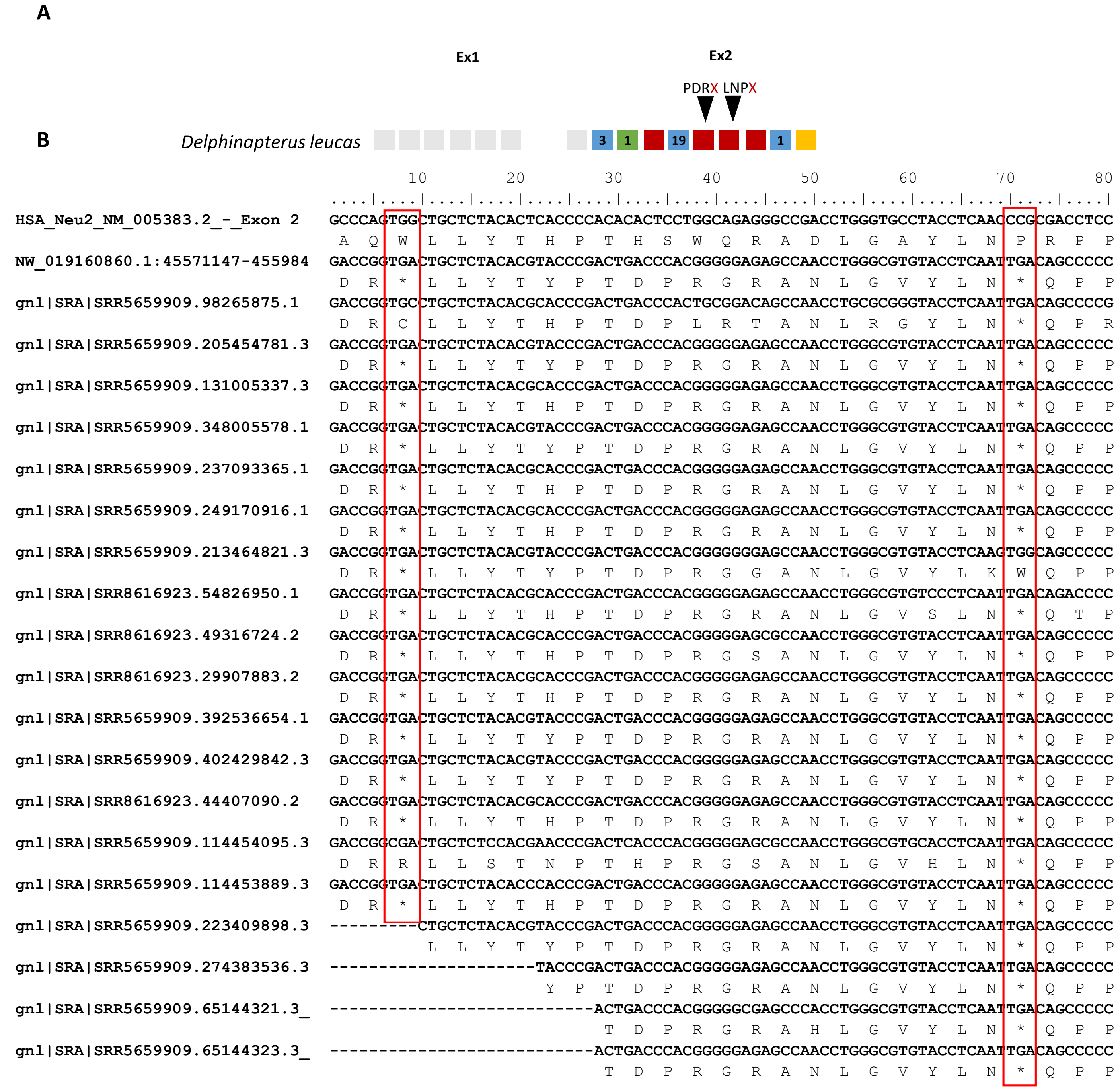

C

Project	Run	Submitted by	DATE	Sample
SRX2896241	SRR5659909	BC Cancer Agency Michael Smith Genome Sciences Centre	2017-06-27	SAMN06216270 SJ_33 female
SRX5415866	SRR8616923	Baylor College of Medicine	2019-02-24	SAMN10973720 0237 female

Figure S39: **A**-Schematic representation of the identified mutations during gene annotation of *NEU2* in *Delphinapterus leucas* using the available genome assembly in NCBI (Scaffold NW_019160860.1). Each group of grey squares represents an exon, number indicated above, yellow indicates loss of canonical splice site (AG-GT), green indicates insertion, blue indicates deletion and read indicates premature stop codon. Numbers in the squares indicate how many nucleotides were inserted or deleted or how many stop codons were identified. **B**-Validation of the identified mutation through multiple sequence alignment of reads obtained from SRA projects. Red box highlights the location of the premature stop codon. **C.** SRA projects consulted.

A

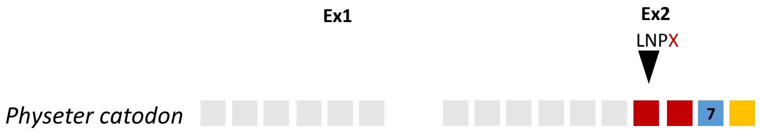

B

```
                                          10        20        30        40        50        60        70
                                 ....|....|....|....|....|....|....|....|....|....|....|....|....|....|....|...
HSA_Neu2_NM_005383.2_-_Exon2     TACCTCAACCCGCGACCTCCAGCCCCTGAGGCCTGGTCAGAGCCGGTACTGCTGGCCAAGGGCAGCTGTGCCTACTCA
                                  Y  L  N  P  R  P  P  A  P  E  A  W  S  E  P  V  L  L  A  K  G  S  C  A  Y  S
NC_041215.1:c132135948-1321213   TACCTCAATTGACAGCGCCCCGCGCCCTAGGCCTGGTCGGAGCCCACCGTGCCGGCCCCGGC-------GCTTACTCA
                                  Y  L  N  *  Q  R  P  A  P  *  A  W  S  E  P  T  V  P  A  P  A        L  T
gnl|SRA|ERR2586194.78402223.1_   TACCTCAATTGACAGCGCCCCGCGCCCTAGGCCTGGTCGGAGCCCACCGTGCCGGCC---------------------
                                  Y  L  N  *  Q  R  P  A  P  *  A  W  S  E  P  T  V  P  A
gnl|SRA|ERR2586194.328356478.1   TACCTCAATTGACAGCGCCCCGCGCCCTAGGCCTGGTCGGAGCCCACCGTGCCGGCCC--------------------
                                  Y  L  N  *  Q  R  P  A  P  *  A  W  S  E  P  T  V  P  A
gnl|SRA|ERR2586194.432512733.2   TACCTCAATTGACAGCGCCCCGCGCCCTAGGCCTGGTCGGAGCCCACCGTGCCGGCCCC-------------------
                                  Y  L  N  *  Q  R  P  A  P  *  A  W  S  E  P  T  V  P  A
gnl|SRA|SRR5146843.1522834.1 1   TACCTCAATTGACAGCGCCCCGCGCCCTAGGCCTGGTCGGAGCCCACCGTGCCGGCCCCGGC-------GCTTACTCA
                                  Y  L  N  *  Q  R  P  A  P  *  A  W  S  E  P  T  V  P  A  P  A        L  T
gnl|SRA|SRR5146847.18512874.1_   TACCTCAATTGACAGCGCCCCGCGCCCTAGGCCTGGTCGGAGCCCACCGTGCCGGCCCCGGC-------GCTTACTCA
                                  Y  L  N  *  Q  R  P  A  P  *  A  W  S  E  P  T  V  P  A  P  A        L  T
gnl|SRA|ERR2586194.437564494.1   TACCTCAATTGACAGCGCCCCGCGCCCTAGGCCTGGTCGGAGCCCACCGTGCCGGCCCCGGC-------GCTTACTCA
                                  Y  L  N  *  Q  R  P  A  P  *  A  W  S  E  P  T  V  P  A  P  A        L  T
gnl|SRA|ERR2586194.67030329.2_   TACCTCAACTGACAGCGCCCCGCGCCCTAGGCCTGGTCGGAGCCCACCGTGCCGGCCCCGGC-------GCTTACTCA
                                  Y  L  N  *  Q  R  P  A  P  *  A  W  S  E  P  T  V  P  A  P  A        L  T
gnl|SRA|SRR5146847.1094555.1 1    TACCTCAATTGACAGCGCCCCGCGCCCTAGGCCTGGTCGGAGCCCACCGTGCCGGCCCCGGC-------GCTTACTCA
                                  Y  L  N  *  Q  R  P  A  P  *  A  W  S  E  P  T  V  P  A  P  A        L  T
gnl|SRA|ERR2586194.396721921.1   TACCTCAATTGACAGCGCCCCGCGCCCTAGGCCTGGTCGGAGCCCACCGTGCCGGCCCCGGC-------GCTTACTCA
                                  Y  L  N  *  Q  R  P  A  P  *  A  W  S  E  P  T  V  P  A  P  A        L  T
gnl|SRA|SRR5146843.23902219.1    TACCTCAATTGACAGCGCCCCGCGCCCTAGGCCTGGTCGGAGCCCACCGTGCCGGCCCCGGC-------GCTTACTCA
                                  Y  L  N  *  Q  R  P  A  P  *  A  W  S  E  P  T  V  P  A  P  A        L  T
gnl|SRA|ERR2586194.31040256.2    TACCTCAATTGACAGCGCCCCGCGCCCTAGGCCTGGTCGGAGCCCACCGTGCCGGCCCCGGC-------TCTTACNCA
                                  Y  L  N  *  Q  R  P  A  P  *  A  W  S  E  P  T  V  P  A  P  A        L  X
gnl|SRA|ERR2586194.442601928.2   TACCTCAATTGACAGCGCCCCGCGCCCTAGGCCTGGTCGGAGCCCACCGTGCCGGCCCCGGC-------GCTTACTCA
                                  Y  L  N  *  Q  R  P  A  P  *  A  W  S  E  P  T  V  P  A  P  A        L  T
gnl|SRA|ERR2586194.328356478.2   TACCTCCATTGACAGCGCCCCGCGCCCTAGGCCTGGTCGGAGCCCACCGTGCCGGCCCCGGC-------GCTTACTCA
                                  Y  L  H  *  Q  R  P  A  P  *  A  W  S  E  P  T  V  P  A  P  A        L  T
gnl|SRA|ERR2586194.158304925.1   ---------TGACAGCGCCCCGCGCCCTAGGCCTGGTCGGAGCCCACCGTGCCGGCCCCGGC-------GCTTACTCA
                                           *  Q  R  P  A  P  *  A  W  S  E  P  T  V  P  A  P  A        L  T
gnl|SRA|ERR2586194.370370218.2   ------------CAGCGCCCCGCGCCCTAGGCCGGGTCGGAGCCCACCGTGCCGGCCCCGGC-------GCTTACTCA
                                              Q  R  P  A  P  *  A  G  S  E  P  T  V  P  A  P  A        L  T
gnl|SRA|ERR2586194.437564494.2   ------------CAGCGCCCCGCGCCCTAGGCCTGGTCGGAGCCCACCGTGCCGGCCCCGGC-------GCTTGCTCA
                                              Q  R  P  A  P  *  A  W  S  E  P  T  V  P  A  P  A        L  A
gnl|SRA|ERR2586194.287557592.2   ---------------CGCCCCGCGCCCTAGGCCTGGTCGGAGCCCACCGTGCCGGCCCCGGC-------GCTTACTCA
                                                 R  P  A  P  *  A  W  S  E  P  T  V  P  A  P  A        L  T
gnl|SRA|ERR2586194.369716066.2   ---------------CGCCCCGCGCCCTAGGCCTGGTCGGAGCCCACCGTGCCGGCCCCGGC-------GCTTACTCA
                                                 R  P  A  P  *  A  W  S  E  P  T  V  P  A  P  A        L  T
gnl|SRA|ERR2586194.477441152.2   ------------------CCCGCGCCCTAGGCCTGGTCGGAGCCCACCGTGCCGGCCCCGGC-------GCTTACTCA
                                                    P  A  P  *  A  W  S  E  P  T  V  P  A  P  A        L  T
gnl|SRA|SRR5146843.35275821.1    ------------------CCCGCGCCCTAGGCCTGGTCGGAGCCCACCGTGCCGGCCCCGGC-------GCTTACTCA
                                                    P  A  P  *  A  W  S  E  P  T  V  P  A  P  A        L  T
gnl|SRA|ERR2586194.115319411.1   ------------------------CCCTAGGCCTGGTCGGAGCCCACCGTGCCGGCCCCGGC-------GCTTACTCA
                                                          P  *  A  W  S  E  P  T  V  P  A  P  A        L  T
gnl|SRA|ERR2586194.260524428.1   ------------------------CCCTAGGCCTGGTCGGAGCCCACCGTGCCGGCCCCGGC-------GCTTACTCA
                                                          P  *  A  W  S  E  P  T  V  P  A  P  A        L  T
```

C

Project	Run	Submitted by	DATE	Sample
SRX2447269	SRR5146847	The Genome Center at Washington University School of Medicine in St. Louis	2017-01-05	SAMN06187412 - 892431
SRX2447270	SRR5146843	The Genome Center at Washington University School of Medicine in St. Louis	2017-01-05	SAMN06187412 - 892431
ERX2601917	ERR2586194	CNGB	2018-07-03	SAMEA1067860 - female

Figure S40: **A**-Schematic representation of the identified mutations during gene annotation of *NEU2* in *Physeter catodon*using the available genome assembly in NCBI (Scaffold NC_041215.1). Each group of grey squares represents an exon, number indicated above, yellow indicates loss of canonical splice site (AG-GT), green indicates insertion, blue indicates deletion and read indicates premature stop codon. Numbers in the squares indicate how many nucleotides were inserted or deleted or how many stop codons were identified. **B**-Validation of the identified mutation through multiple sequence alignment of reads obtained from SRA projects. Red box highlights the location of the premature stop codon. **C.** SRA projects consulted.

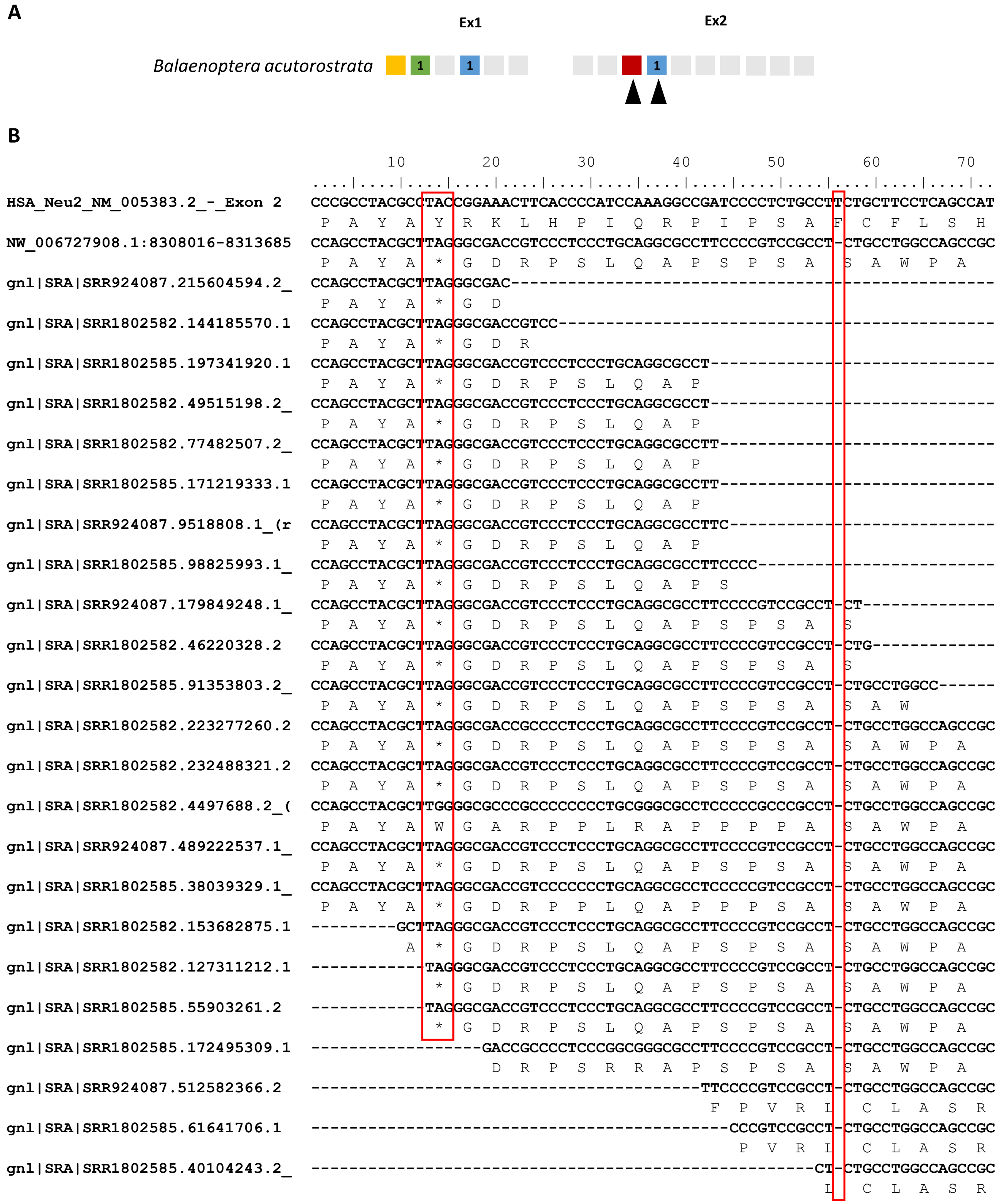

C

Project	Run	Submitted by	DATE	Sample
SRX316738	SRR924087	Korea Institute of Ocean Science and Technology	2013-10-31	SAMN02192644 – MinkeWhale-01
SRX872204	SRR1802582	Seoul National University	2016-02-11	SAMN03339797- MW30
SRX872217	SRR1802585	Seoul National University	2016-02-11	SAMN03339800- MW37

Figure S41: **A**-Schematic representation of the identified mutations during gene annotation of *NEU2* in *Balaenoptera acutorostrata* using the available genome assembly in NCBI (Scaffold NW_006727908.1). Each group of grey squares represents an exon, number indicated above, yellow indicates loss of canonical splice site (AG-GT), green indicates insertion, blue indicates deletion and read indicates premature stop codon. Numbers in the squares indicate how many nucleotides were inserted or deleted or how many stop codons were identified. **B**-Validation of the identified mutation through multiple sequence alignment of reads obtained from SRA projects. Red box highlights the location of the premature stop codon. **C.** SRA projects consulted.

A

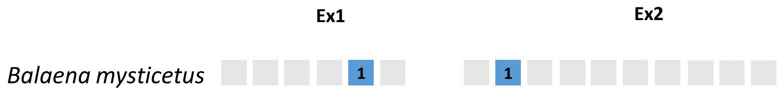

B

```
                                         10        20        30        40        50        60
                                ....|....|....|....|....|....|....|....|....|....|....|....|....
HSA_Neu2_NM_005383.2_-_Exon 2   GTGACGCGGCTGTGCCAAGTCACCAGCACTGACCACGGGAGGACCTGGAGCTCCCCCAGAGACCTCACT
                                 V  T  R  L  C  Q  V  T  S  T  D  H  G  R  T  W  S  S  P  R  D  L  T
gnl|BL_ORD_ID|1272 scaffold_16  GTGACACGGCTGTGCCAGGTCACCAGCAC-GACCACGGGAGGTCCTGGAGCCCCATCAGGGACCTCACG
                                 V  T  R  L  C  Q  V  T  S  T   T  T  G  G  P  G  A  P  S  G  T  S
gnl|SRA|SRR1685385.116793653.1  GTGACACGGCTGTGCCAGGTCACCATCAC-GACCACGGGAGGTCCTG----------------------
                                 V  T  R  L  C  Q  V  T  I  T   T  T  G  G  P
gnl|SRA|SRR1685385.214901507.1  GTGACACGGCTGTGCCAGGTCACCAGCAC-GACCACG--------------------------------
                                 V  T  R  L  C  Q  V  T  S  T   T  T
gnl|SRA|SRR1685385.227485501.1  GTGACACGGCTGTGCCAGGTCACCAGCAC-GACCACGGGAGGTCCTGGAGCCCCATCA-----------
                                 V  T  R  L  C  Q  V  T  S  T   T  T  G  G  P  G  A  P  S
gnl|SRA|SRR1685385.176537450.1  GTGACACGGCTGTGCCAGGTCACCAGCAC-GACCACGGGAGGTCCTGGAGCCCCATCA-----------
                                 V  T  R  L  C  Q  V  T  S  T   T  T  G  G  P  G  A  P  S
gnl|SRA|SRR1685385.318170661.1  GTGACACGGCTGTGCCAGGTCACCAGCAC-GACCACGGGAGGTCCTGGAGCCCCATCAGGGACCTCACG
                                 V  T  R  L  C  Q  V  T  S  T   T  T  G  G  P  G  A  P  S  G  T  S
gnl|SRA|SRR1685385.318705056.1  GTGACACGGCTGTGCCAGGTCACGAGCAC-GACCACGGGAGGTCCTGGAGCCCCATCAGGGACCTCACG
                                 V  T  R  L  C  Q  V  T  S  T   T  T  G  G  P  G  A  P  S  G  T  S
gnl|SRA|SRR1685385.192870377.1  GTGACACGGCTGTGCCAGGTCACCAGCAC-GACCACGGGAGGTCCTGGAGCCCCATCAGGGACCTCACG
                                 V  T  R  L  C  Q  V  T  S  T   T  T  G  G  P  G  A  P  S  G  T  S
gnl|SRA|SRR1685385.242758677.2  ------CGGCTGTGCCAGGTCACCAGCAC-GACCACGGGAGGTCCTGGAGCCCCATCAGGGACCTCACG
                                       R  L  C  Q  V  T  S  T   T  T  G  G  P  G  A  P  S  G  T  S
gnl|SRA|SRR1685385.111402216.2  ------------TGCCAGGTCACCAGCAC-GACCACGGGAGGTCCTGGAGCCCCATCAGGGACCTCACG
                                             C  Q  V  T  S  T   T  T  G  G  P  G  A  P  S  G  T  S
```

C

Project	Run	Submitted by	DATE	Sample
SRX790317	SRR1685385	University of Liverpool	2015-01-06	SAMN03225705 –ID325 female

Figure S42: **A**-Schematic representation of the identified mutations during gene annotation of *NEU2* in *Balaena mysticetus* using the available genome assembly in NCBI (Scaffold 1670). Each group of grey squares represents an exon, number indicated above, yellow indicates loss of canonical splice site (AG-GT), green indicates insertion, blue indicates deletion and read indicates premature stop codon. Numbers in the squares indicate how many nucleotides were inserted or deleted or how many stop codons were identified. **B**-Validation of the identified mutation through multiple sequence alignment of reads obtained from SRA projects. Red box highlights the location of the premature stop codon. **C.** SRA projects consulted.

SUPPLEMENTARY MATERIAL- 9

A

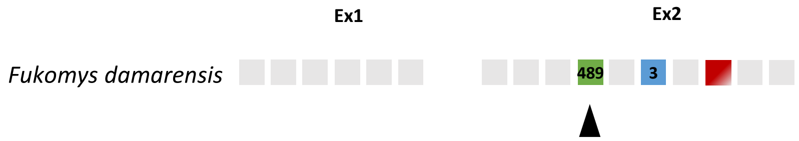

B

```
                                           10        20        30        40        50        60        70        80
                                   ....|....|....|....|....|....|....|....|....|....|....|....|....|....|....|....|....
HSA_Neu2_NM_005383.2_Exon 2        TTCCTCAGCCATGACCATGGGCGCACGTGGGCG---------------------CGAGGGCACTTTGTGGCCCAGGACACCCTG
                                    F  L  S  H  D  H  G  R  T  W  A                       R  G  H  F  V  A  Q  D  T  L
NW_011045114.1:1186015-1189000     TTCGTCAGCCATGACCGAGGGCGCACGTGGGCGTGGAGCAGC...ACATGGGCGCGGAGTAGCTTCGTGGGCCAGGGCGCCGTG
                                    F  V  S  H  D  R  G  R  T  W  A  W  S  S ...  T  W  A  R  S  S  F  V  G  Q  G  A  V
NW_022900928.1:130265313-13026     TTCGTCAGCCATGACCGAGGGCGCACGTGGGCGTGGAGCAGC...ACATGGGCGCGGAGTAGCTTCGTGGGCCAGGGCGCCGTG
                                    F  V  S  H  D  R  G  R  T  W  A  W  S  S ...  T  W  A  R  S  S  F  V  G  Q  G  A  V
gnl|SRA|SRR8616929.59263027.2_     TTCGTCAGCCACGACCGAGGGCGCACATGGGCG---------------------CGGAGTA-----------------------
                                    F  V  S  H  D  R  G  R  T  W  A                       R  S
gnl|SRA|SRR960028.138142210.1_     TTCGTCAGCCATGACCGAGGGCGCACGTGGGCG---------------------TGGAGCAGCT--------------------
                                    F  V  S  H  D  R  G  R  T  W  A                       W  S  S
gnl|SRA|SRR960028.43425477.1_e     TTCGTCAGCCATGACCGAGGGCGCACGTGGGCG---------------------TGGAGCAGCT--------------------
                                    F  V  S  H  D  R  G  R  T  W  A                       W  S  S
gnl|SRA|SRR8616929.1163205.2_e     TTCGTCAGCCCTGACCGAGGGCGCACGTGGGCG---------------------TGGCGCAGCTTCGTGGGCC-----------
                                    F  V  S  P  D  R  G  R  T  W  A                       W  R  S  F  V  G
gnl|SRA|SRR8616929.10165954.1_     TTCGTCAGCCACGACCGAGGGCGCACATGGGCG---------------------CGGAGTAGCTTCGTGGGCC-----------
                                    F  V  S  H  D  R  G  R  T  W  A                       R  S  S  F  V  G
gnl|SRA|SRR960028.69662354.1_e     TTCGTCAGCCATGACCGAGGGCGCACGTGGGCG---------------------TGGAGCAGCTTCGTGGGCCAGGGCGCCGTG
                                    F  V  S  H  D  R  G  R  T  W  A                       W  S  S  F  V  G  Q  G  A  V
gnl|SRA|SRR8616929.39392343.1_     TTCGTCAGCCACGACCGAGGGCGCACATGGGCG---------------------CGGAGTAGCTTCGTGGGCCAGGGCGCCGTG
                                    F  V  S  H  D  R  G  R  T  W  A                       R  S  S  F  V  G  Q  G  A  V
gnl|SRA|SRR960028.72598218.1_e     TTCGTCAGCCATGACCGAGGGCGCACGTGGGCG---------------------TGGAGCAGCTTCGTGGGCCAGGGCGCCGTG
                                    F  V  S  H  D  R  G  R  T  W  A                       W  S  S  F  V  G  Q  G  A  V
gnl|SRA|SRR960028.123451679.1_     TTCGTCAGCCACGACCGAGGGCGCACATGGGCG---------------------CGGAGTAGCTTCGTGGGCCAGGGCGCCGTG
                                    F  V  S  H  D  R  G  R  T  W  A                       R  S  S  F  V  G  Q  G  A  V
gnl|SRA|SRR960028.50632861.1_e     TTCGTCAGCCATGACCGAGGGCGCACGTGGGCG---------------------TGGAGCAGCTTCGTGGGCCAGGGCGCCGTG
                                    F  V  S  H  D  R  G  R  T  W  A                       W  S  S  F  V  G  Q  G  A  V
gnl|SRA|SRR960030.43442164.1_e     TTCGTCAGCCATGACCGAGGGCGCACGTGGGCG---------------------TGGAGCAGCTTCGTGGGCCAGGGCGCCGTG
                                    F  V  S  H  D  R  G  R  T  W  A                       W  S  S  F  V  G  Q  G  A  V
gnl|SRA|SRR960028.41272950.1_e     TTCGTCAGCCATGACCGAGGGCGCACGTGGGCG---------------------TGGAGCAGCTTCGTGGGCCAGGGCGCCGTG
                                    F  V  S  H  D  R  G  R  T  W  A                       W  S  S  F  V  G  Q  G  A  V
gnl|SRA|SRR960030.61718831.2_e     TTCGTCAGCCATGACCGAGGGCGCACGTGGGCG---------------------TGGAGCAGCTTCGTGGGCCAGGGCGCCGTG
                                    F  V  S  H  D  R  G  R  T  W  A                       W  S  S  F  V  G  Q  G  A  V
gnl|SRA|SRR960028.117754214.1_     TTCGTCAGCCATGACCGAGGGCGCACGTGGGCG---------------------TGGAGCAGCTTCGTGGGCCAGGGCGCCGTG
                                    F  V  S  H  D  R  G  R  T  W  A                       W  S  S  F  V  G  Q  G  A  V
gnl|SRA|SRR960030.84484952.1_e     TTCGTCAGCCATGACCGAGGGCGCACGTGGGCG---------------------TGGAGCAGCTTCGTGGGCCAGGGCGCCGTG
                                    F  V  S  H  D  R  G  R  T  W  A                       W  S  S  F  V  G  Q  G  A  V
gnl|SRA|SRR960028.69662354.2_e     ------AGCCATGACCGAGGGCGCACGTGGGCG---------------------TGGAGCAGCTTCGTGGGCCAGGGCGCCGTG
                                          S  H  D  R  G  R  T  W  A                       W  S  S  F  V  G  Q  G  A  V
gnl|SRA|SRR960028.87420536.2_e     ---------CATGACCGAGGGCGCACGTGGGCG---------------------TGGAGCAGCTTCGTGGGCCAGGGCGCCGTG
                                             H  D  R  G  R  T  W  A                       W  S  S  F  V  G  Q  G  A  V
gnl|SRA|SRR960028.58367977.2_e     ------------------------------------------------------------TTCGTGGGCCAGGGCGCCGTG
                                                                                                   F  V  G  Q  G  A  V
```

C

Project	Run	Submitted By	DATE	Sample
SRX341884	SRR960030<br>SRR960028	Beijing Genome Institute (BGI)	2015-07-22	SAMN02339281
SRX5415860	SRR8616929	Baylor College of Medicine	2019-02-24	SAMN10973724

Figure S43: A-Schematic representation of the identified mutations during gene annotation of NEU2 in *Fukomys damarensis* using the available genome assemblies in NCBI (NW_011045114.1 & NW_022900928.1). Each group of grey squares represents an exon, number indicated above, yellow indicates loss of canonical splice site (AG-GT), green indicates insertion, blue indicates deletion and read indicates premature stop codon. Numbers in the squares indicate how many nucleotides were inserted or deleted or how many stop codons were identified. B- Non-validation of the identified mutation through multiple sequence alignment of reads obtained from SRA projects. Red box highlights the location of the premature stop codon. C. SRA projects consulted.

A

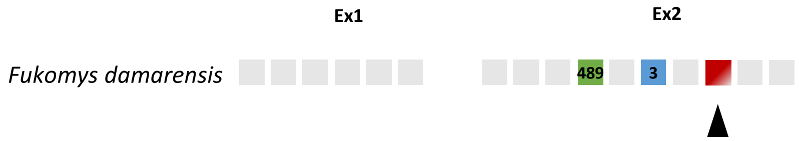

B

```
                                                10        20        30        40        50        60
                                        ....|....|....|....|....|....|....|....|....|....|....|....|
HSA_Neu2_NM_005383.2 Exon 2             GATGGGTCCCCCTTGTTTGGGTGTCTGTACGAAGCCAATGATTACGAGGAGATTGTCTTT
                                         D  G  S  P  L  F  G  C  L  Y  E  A  N  D  Y  E  E  I  V  F
NW_011045114.1:1186015-1189000          GACGGCTCCCCTCAGTTCGGCTGCCTGTAAGAGGCGAAAAGCTATGGAGAGATGGTCTTC
                                         D  G  S  P  Q  F  G  C  L  *  E  A  K  S  Y  G  E  M  V  F
NW_022900928.1:130265313-13026          GACGGCTCCCCTCAGTTCGGCTGCCTGTAAGAGGCGAAAAGCTATGGAGAGATGGTCTTC
                                         D  G  S  P  Q  F  G  C  L  *  E  A  K  S  Y  G  E  M  V  F
gnl|SRA|SRR960028.36985362.2_e          GACGCCTCCCCTCAGTGCGGCTGCCTGTAAGAGGCGAAAAGCTATGGAGAGATGGTCTTC
                                         D  A  S  P  Q  C  G  C  L  *  E  A  K  S  Y  G  E  M  V  F
gnl|SRA|SRR960030.88910761.1_e          GACGGCTCCCCTCAGTTCGGCTGCCTGTAAGAGGCGAAAAGCTATGGAGAGATGGTCTTC
                                         D  G  S  P  Q  F  G  C  L  *  E  A  K  S  Y  G  E  M  V  F
gnl|SRA|SRR960028.97526855.1_e          GACGGCTCCCCTCAGTTCGGCTGCCTGTAAGAGGCGAAAAGCTATGGAGAGATGGTCTTC
                                         D  G  S  P  Q  F  G  C  L  *  E  A  K  S  Y  G  E  M  V  F
gnl|SRA|SRR8616943.31186514.2_          GACGGCTCCCCTCAGTTCGGCTGCCTGTAAGAGGCGAAAAGCTATGGAGAGATGGTCTTC
                                         D  G  S  P  Q  F  G  C  L  *  E  A  K  S  Y  G  E  M  V  F
gnl|SRA|SRR960030.160392222.1_          GACGGCTCCCCTCAGTTCGGCTGCCTGTACGAGGCGAAAAGCTATGGAGAGATGGTCTTC
                                         D  G  S  P  Q  F  G  C  L  Y  E  A  K  S  Y  G  E  M  V  F
gnl|SRA|SRR960028.57484091.1_e          GACGGCTCCCCTCAGTTCGGCTGCCTGTAAGAGGCGAAAAGCTATGGAGAGATGGTCTTC
                                         D  G  S  P  Q  F  G  C  L  *  E  A  K  S  Y  G  E  M  V  F
gnl|SRA|SRR960030.8797189.2_ex          GACGGCTCCCCTCAGTTCGGCTGCCTGTAAGAGGCGAAAAGCTATGGAGAGATGGTCTTC
                                         D  G  S  P  Q  F  G  C  L  *  E  A  K  S  Y  G  E  M  V  F
gnl|SRA|SRR960030.132800200.2_          GACGGCTCCCCTCAGTTCGGCTGCCTGTAAGAGGCGAAAAGCTATGGAGAGATGGTCTTC
                                         D  G  S  P  Q  F  G  C  L  *  E  A  K  S  Y  G  E  M  V  F
gnl|SRA|SRR8616929.87558957.1_          GACGGCTCCCCTCAGTTCGGCTGCCTGTACGAGGCGAAAAGCTATGGAGAGATGGTCTTC
                                         D  G  S  P  Q  F  G  C  L  Y  E  A  K  S  Y  G  E  M  V  F
gnl|SRA|SRR960030.87862415.2_e          GACGGCTCCCCTCAGTTCGGCTGCCTGTAAGAGGCGAAAAGCTATGGAGAGATGGTCTTC
                                         D  G  S  P  Q  F  G  C  L  *  E  A  K  S  Y  G  E  M  V  F
gnl|SRA|SRR960030.154008665.1_          GACGGCTCCCCTCAGTTCGGCTGCCTGTAAGAGGCGAAAAGCTATGGAGAGATGGTCTTC
                                         D  G  S  P  Q  F  G  C  L  *  E  A  K  S  Y  G  E  M  V  F
gnl|SRA|SRR960030.115854205.1_          GACGGCTCCCCTCAGTTCGGCTGCCTGTAAGAGGCGAAAAGCTATGGAGAGATGGTCTTC
                                         D  G  S  P  Q  F  G  C  L  *  E  A  K  S  Y  G  E  M  V  F
gnl|SRA|SRR8616929.47637505.1_          GACGGCTCCCCTCAGTTCGGCTGCCTGTAAGAGGCGAAAAGCTATGGAGAGATGGTCTTC
                                         D  G  S  P  Q  F  G  C  L  *  E  A  K  S  Y  G  E  M  V  F
gnl|SRA|SRR8616929.8046437.1_e          GACGGCTCCCCTCAGTTCGGCTGCCTGTAAGAGGCGAAAAGCTATGGAGAGATGGTCTTC
                                         D  G  S  P  Q  F  G  C  L  *  E  A  K  S  Y  G  E  M  V  F
gnl|SRA|SRR8616929.19141132.2_          GACGGCTCCCCTCAGTTCGGCTGCCTGTACGAGGCGAAAAGCTATGGAGAGATGGTCTTC
                                         D  G  S  P  Q  F  G  C  L  Y  E  A  K  S  Y  G  E  M  V  F
gnl|SRA|SRR8616943.53750733.1_          GACGGCTCCCCTCAGTTCGGCTGCCTGTACGAGGCGAAAAGCTATGGAGAGATGGTCTTC
                                         D  G  S  P  Q  F  G  C  L  Y  E  A  K  S  Y  G  E  M  V  F
gnl|SRA|SRR960028.105704669.1_          GACGGCTCCCCTCAGTTCGGCTGCCTGTACGAGGCGAAAAGCTATGGAGAGATGGTCTTC
                                         D  G  S  P  Q  F  G  C  L  Y  E  A  K  S  Y  G  E  M  V  F
gnl|SRA|SRR960030.66365336.2_e          GACGGCTCCCCTCAGTTCGGCTGCCTGTACGAGGCGAAAAGCTATGGAGAGATGGTCTTC
                                         D  G  S  P  Q  F  G  C  L  Y  E  A  K  S  Y  G  E  M  V  F
gnl|SRA|SRR960028.134980096.1_          GACGGCTCCCCTCAGTTCGGCTGCCTGTAAGAGGCGAAAAGCTATGGAGAGATGGTCTTC
                                         D  G  S  P  Q  F  G  C  L  *  E  A  K  S  Y  G  E  M  V  F
gnl|SRA|SRR960030.80939454.1_e          GACGGCTCCCCTCAGTTCGGCTGCCTGTAAGAGGCGAAAAGCTATGGAGAGATGGTCTTC
                                         D  G  S  P  Q  F  G  C  L  *  E  A  K  S  Y  G  E  M  V  F
gnl|SRA|SRR960030.42977489.1_e          GACGGCTCCCCTCAGTTCGGCTGCCTGTACGAGGCGAAAAGCTATGGAGAGATGGTCTTC
                                         D  G  S  P  Q  F  G  C  L  Y  E  A  K  S  Y  G  E  M  V  F
gnl|SRA|SRR8616943.30459959.1_          GACGGCTCCCCTCAGTTCGGCTGCCTGTAAGAGGCGAAAAGCTATGGAGAGATGGTCTTC
                                         D  G  S  P  Q  F  G  C  L  *  E  A  K  S  Y  G  E  M  V  F
gnl|SRA|SRR960030.98874245.1_e          GACGGCTCCCCTCAGTTCGGCTGCCTGTACGAGGCGAAAAGCTATGGAGAGATGGTCTTC
                                         D  G  S  P  Q  F  G  C  L  Y  E  A  K  S  Y  G  E  M  V  F
gnl|SRA|SRR960030.100315530.1_          GACGGCTCCCCTCAGTTCGGCTGCCTGTAAGAGGCGAAAAGCTATGGAGAGGTGGTCTTC
                                         D  G  S  P  Q  F  G  C  L  *  E  A  K  S  Y  G  E  V  V  F
gnl|SRA|SRR8616929.18421299.1_          GACGGCTCCCCTCAGTTCGGCTGCCTGTAAGAGGCGAAAAGCTATGGAGAGATGGTCTTC
                                         D  G  S  P  Q  F  G  C  L  *  E  A  K  S  Y  G  E  M  V  F
gnl|SRA|SRR960028.12097508.2_e          GACGCCTCCCCTCAGTTCGGCTGCCTGTACGAGGCGAAAAGCTATGGAGAGATGGTCTTC
                                         D  A  S  P  Q  F  G  C  L  Y  E  A  K  S  Y  G  E  M  V  F
gnl|SRA|SRR960028.99706385.1_e          GACGGCTCCCCTCAGTTCGGCTGCCTGTACGAGGCGAAAAGCTATGGAGAGATGGTCTTC
                                         D  G  S  P  Q  F  G  C  L  Y  E  A  K  S  Y  G  E  M  V  F
```

C

Project	Run	Submitted By	DATE	Sample
SRX341884	SRR960030<br>SRR960028	Beijing Genome Institute (BGI)	2015-07-22	SAMN02339281 Heterozygous
SRX5415860	SRR8616929	Baylor College of Medicine	2019-02-24	SAMN10973724 Heterozygous

Figure S44: A-Schematic representation of the identified mutations during gene annotation of NEU2 in *Fukomys damarensis* using the available genome assemblies in NCBI (NW_011045114.1 & NW_022900928.1). Each group of grey squares represents an exon, number indicated above, yellow indicates loss of canonical splice site (AG-GT), green indicates insertion, blue indicates deletion and read indicates premature stop codon. Numbers in the squares indicate how many nucleotides were inserted or deleted or how many stop codons were identified. B- Validation of the identified mutation through multiple sequence alignment of reads obtained from SRA projects. Red box highlights the location of the premature stop codon. C. SRA projects consulted.
